# A comparison of self-reported and device measured sedentary behaviour in adults: a systematic review and meta-analysis

**DOI:** 10.1186/s12966-020-00938-3

**Published:** 2020-03-04

**Authors:** Stephanie A. Prince, Luca Cardilli, Jennifer L. Reed, Travis J. Saunders, Chris Kite, Kevin Douillette, Karine Fournier, John P. Buckley

**Affiliations:** 1 Division of Cardiac Prevention and Rehabilitation, University of Ottawa Heart Institute, Ottawa, Canada; 2 Centre for Surveillance and Applied Research, Public Health Agency of Canada, 785 Carling Avenue, Ottawa, K1A 0K9 Canada; 3 Birmingham Community Healthcare NHS Foundation Trust, Community Cardiac Services, Birmingham, United Kingdom; 4 Centre for Active Living, University Centre Shrewsbury, University of Chester, Guildhall, Frankwell Quay, Shrewsbury, United Kingdom; 5 School of Human Kinetics, Faculty of Health Sciences, University of Ottawa, Ottawa, Canada; 6 School of Epidemiology and Public Health, Faculty of Medicine, University of Ottawa, Ottawa, Canada; 7 Department of Applied Human Sciences, University of Prince Edward Island, Charlottetown, Canada; 8 School of Life and Health Sciences, Aston University, Birmingham, United Kingdom; 9 Health Sciences Library, University of Ottawa, Ottawa, Canada

**Keywords:** Self-report, device, sedentary behaviour, systematic review

## Abstract

**Background:**

Sedentary behaviour (SB) is a risk factor for chronic disease and premature mortality. While many individual studies have examined the reliability and validity of various self-report measures for assessing SB, it is not clear, in general, how self-reported SB (e.g., questionnaires, logs, ecological momentary assessments (EMAs)) compares to device measures (e.g., accelerometers, inclinometers).

**Objective:**

The primary objective of this systematic review was to compare self-report versus device measures of SB in adults.

**Methods:**

Six bibliographic databases were searched to identify all studies which included a comparable self-report and device measure of SB in adults. Risk of bias within and across studies was assessed. Results were synthesized using meta-analyses.

**Results:**

The review included 185 unique studies. A total of 123 studies comprising 173 comparisons and data from 55,199 participants were used to examine general criterion validity. The average mean difference was -105.19 minutes/day (95% CI: -127.21, -83.17); self-report underestimated sedentary time by ~1.74 hours/day compared to device measures. Self-reported time spent sedentary at work was ~40 minutes higher than when assessed by devices. Single item measures performed more poorly than multi-item questionnaires, EMAs and logs/diaries. On average, when compared to inclinometers, multi-item questionnaires, EMAs and logs/diaries were not significantly different, but had substantial amount of variability (up to 6 hours/day within individual studies) with approximately half over-reporting and half under-reporting. A total of 54 studies provided an assessment of reliability of a self-report measure, on average the reliability was good (ICC = 0.66).

**Conclusions:**

Evidence from this review suggests that single-item self-report measures generally underestimate sedentary time when compared to device measures. For accuracy, multi-item questionnaires, EMAs and logs/diaries with a shorter recall period should be encouraged above single item questions and longer recall periods if sedentary time is a primary outcome of study. Users should also be aware of the high degree of variability between and within tools. Studies should exert caution when comparing associations between different self-report and device measures with health outcomes.

**Systematic review registration:**

PROSPERO CRD42019118755

## Introduction

Sedentary behaviour (SB) is considered a risk factor for chronic disease and premature mortality [1]. This area of enquiry has, therefore, gained much attention in the past decade with a dramatic increase in the amount of research and surveillance being performed [2]. Often the term sedentary is assumed as a general description for classifying people with low levels of physical activity (PA), but it is a distinct term referring to any wakeful state of sitting, lying or reclining where energy expenditure is ≤ 1.5 metabolic equivalents (METs) [3]. It is, therefore, possible for people to be regularly physically active and yet have high levels of SB.

Ekelund et al. (2016) performed a large observational study to assess the interactions between SB and PA, and reported that the associated risk of prolonged sitting was nullified once individuals performed 60-75 minutes per day of moderate-to-vigorous intensity physical activity (MVPA) [4]. Current PA guidelines promote a threshold of 150 minutes of MVPA per week [5] which is far less than the aforementioned 60-75 minutes/day. Very few adults today (especially in high income countries) meet these MVPA guidelines and most spend the majority of their days sedentary [6–8]. This suggests that large proportions of adults, even those who exercise regularly and within the PA guidelines, could still be exposed to the health risks of prolonged sitting.

As with PA [9], the largest challenge to the validity of the link between SB and health is the measures. While direct measurement of SB using accelerometers and inclinometers has grown increasingly popular, the cost-to-utility ratio has remained fairly high with often prohibitive device and resource costs and a required proximity to respondents [10, 11]. Monitoring of SB by self-report remains the most practical means for most national surveillance systems [12] and research studies; contextual information that is often missing from device-assessed SB such as domain (i.e., occupational, transportation, household) and type (e.g., watching television, sitting and playing the piano). It is widely recognized that self-reported PA estimates are often higher than when directly measured (e.g., accelerometer, pedometer, doubly-labeled water, heart rate monitors) [9, 13]. Previous works have described various measures of SB and their properties [10, 11, 14]. Dall et al. developed a framework (Taxonomy of Self-reported Sedentary behaviour Tools [TASST]) for identifying the optimal self-report tool for population surveillance of SB [14]. Their work found that in the majority of cases, there is a large underestimation of SB from self-report and that composite questionnaires appear to perform better than single questions [14]. To our knowledge there has been no attempt to synthesize the literature to determine how self-report measures compare to device-assessed measures of SB (criterion validity), nor to assess their reliability in adult populations. Therefore, the primary objective of this systematic review was to compare self-report versus device measures of SB in adult populations. The secondary objective was to assess the reliability of self-report measures.

## Methods

The review was prospectively registered with PROSPERO (#CRD42019118755).

### Study inclusion criteria

#### Population

Adults with a mean age ≥ 18 years.

#### Self-report measures

Self-report measures may include: diaries or logs (daily reporting of the current/past day’s activities possibly in segments of time e.g., between 8:00 am and 10:00 am, 10:01 am to 12:00 pm, etc.); questionnaires (measures designed to collect information about SB either exclusively (e.g., Sedentary Behaviour Questionnaire [SBQ]) or along with other activity intensities (e.g., Global Physical Activity Questionnaire [GPAQ], International Physical Activity Questionnaire [IPAQ]); surveys (self-report measure which may include one or more questions on SB); ecological momentary assessments (EMAs; real-time reporting of activities often done with the use of a web- or phone-based app), and, recall interviews (an individual is asked to verbally recall past events). Following the TASST framework proposed by Dall et al. [14], self-report measures may consist of a single item (generates all information about SB using a single question, e.g., total sitting or single behaviour related to SB) or be composite (multiple questions about several aspects of SB used to generate a total amount/sum of SB over the same recall period).

#### Device measures

Accelerometers (devices that capture “time-varying changes in force” where pre-established cut-points such as < 100 counts-per-minute are used to classify sedentary time based on energy expenditure [11], e.g., ActiGraph, SenseWear), inclinometers (devices (thigh worn) that capture inclination/postural information used to define sitting, standing and lying [15, 16], e.g., activPAL, ActiGraph GT3X+, ActiReg), pedometers (step counters where SB is defined as time spent at less than 100 steps/minute or with no light activity), heart rate monitors (often combined with accelerometers are able to estimate energy expenditure which can be used to identify sedentary time [17, 18], e.g., ActiGraph ActiTrainer), television monitors (records the amount of time in which a television is turned on – may capture non-sedentary time or time when another person in the household is watching television), car monitors (devices that can record driving activity including driving time [19], e.g. CarChip E/X), and wearable cameras (a camera is worn at chest level and takes regular photos providing a snapshot of activities throughout wear time, they may also be combined with accelerometers [20], e.g., Autographer). Some devices include both an accelerometer and an inclinometer and can capture both movement intensity and postural positions (e.g., activPAL, ActiGraph GT3X+). If a study provided device-assessed SB using output from an accelerometer and an inclinometer, the inclinometer data was chosen by default as it is considered the gold standard for field-based measurement of SB and a more accurate criterion measure [21, 22]. No limitation was placed on the location in which the body-worn devices were placed (e.g., waist, hip, wrist or arm), but this data was extracted for a sensitivity analysis.

#### Outcomes

The primary outcome was minutes per day spent in SB. SB could include total sedentary time, total sitting time, lying time (not sleeping), reading, television/video watching, passive video games, screen time, computer time, passive transportation (e.g., driving/sitting in a car, bus train, plane), etc. Measures needed to be comparable (e.g., self-reported sitting vs. device-assessed sitting or sedentary time). Units were standardized where possible (e.g., conversion of minutes/week to minutes/day). Medians and interquartile range were included by equating the median to the mean and dividing the interquartile range by 1.35 (SD = Q3 – Q1/1.35) [23]. Measures of comparison included correlation coefficients, mean differences and 95% confidence intervals (CIs), and Bland-Altman analyses.

#### Study designs

Observational (e.g., cross-sectional, prospective cohort, retrospective cohort) and experimental (e.g., baseline data from randomized controlled trials or quasi-experimental trials) studies that compared the amount of time spent in SB between a self-report and device measure were eligible.

#### Publication status and language

Both published (peer-reviewed) and unpublished grey literature (e.g., abstracts) were eligible. No language restrictions were imposed on the search, but only papers published in English or French were included due to translation limitations.

### Exclusion criteria

Studies in which the mean age was < 18 years, included animals, or which did not compare two measures or in which outcome data from the two measures could not be extracted, and those from which the types of SB were not comparable (e.g., activPAL total sitting vs. self-reported television watching is not comparable whereas self-reported television watching vs. a television monitoring device or wearable camera would be) were excluded from the review.

### Search strategy

The search strategy was created by an information specialist (KF) in discussion with members from the authorship team (SAP, LC, JLR, TJS, JPB). The search was first created in MEDLINE using a combination of index terms/unique subject headings and keywords related to the self-report and device measures and SB. Main concepts searched were “sedentary behaviours”, “self-report measures”, and “device measures”. Only adult and human populations were captured, and a comparison of the two types of measures (self-reported or device) or data to compare the two measures had to be performed for the study to be retained. Once the search was finalized (see Supplemental Table 1 for MEDLINE search strategy), it was translated to the other bibliographic databases.

Searches were conducted from database inception to January 9, 2019 in MEDLINE(R) ALL (Ovid), EMBASE (Embase Classic + Embase, Ovid), PsycINFO (Ovid), CINAHL (EBSCO), SPORTDiscus (EBSCO), and Dissertations & Theses Global (Proquest). Search results were exported to EndNote X8 (Thompson Reuters, San Francisco, CA, USA) and duplicates removed through manual inspection using the EndNote duplicate identification function.

### Selection of studies

Following the initial search, titles and abstracts were exported from EndNote X8 into Covidence (Veritas Health Innovation, Melbourne, Australia) software where further duplicates were removed. Two reviewers independently reviewed all titles and abstracts (SAP, LC, JLR, CK, TJS, JPB) and full texts (SAP, LC, JLR, CK, TJS, JPB). A third reviewer was consulted if disagreements occurred. Reviewers were not blinded to the authors of the studies.

### Data extraction and analysis

Data extraction was completed by one reviewer (SAP) and verified by a second (LC, JLR, CK, TJS, KD, JPB) using standardized data extraction forms in Google Sheets. Information extracted included: publication details; participant characteristics (e.g., age, sex, population), country of study; sample size analyzed (total, male, female); study design; behaviour examined (e.g., sedentary time, sitting time, television time, screen, occupation, passive travel, etc.); self-report measure type (e.g., questionnaire [single or multicomponent], survey, recall interview, diary/log, EMA) and name (e.g., Sedentary Behaviour Questionnaire [SBQ], International Physical Activity Questionnaire [IPAQ]); device measure type (e.g., accelerometer) and name and model (e.g., ActiGraph GT3X, activPAL3); quantity of behaviour within each measure and measure of variance (e.g., minutes and standard deviation [SD], median and interquartile range); units of measurement (e.g., minutes/day, minutes/week); analytical techniques used to compare measures (e.g., correlation, mean difference, t-test, Bland-Altman); result of comparison (e.g., test statistics); and, reliability assessment details (e.g., time between measures, analytical technique and result).

A narrative synthesis, including summary tables, was used to examine all study results. Forest plots and meta-analyses were created using Review Manager Version 5.3.5 (Copenhagen: The Nordic Cochrane Centre) to compare mean differences (minutes/day) and 95% CIs in time spent sedentary between self-report and device-assessed measures. Studies that reported units that could not be converted to minutes per day, that did not provide measures of variance (e.g., SD, 95% CIs), or did not provide results for comparison were not included in the meta-analyses. If a study reported on weekday, weekend, leisure or occupational-specific time they were included in separate meta-analyses. A minimum of two studies were required to conduct a meta-analysis. Several studies [24–30] included multiple self-report measures compared to one device-assessed outcome (e.g., IPAQ and SBQ compared to activPAL), in this case, the applicable ‘arms’ were included in the appropriate meta-analysis and the device measure group was split to include half in each comparison as per the Cochrane handbook [23]. If a study reported device-assessed results from both an accelerometer and an inclinometer, the inclinometer data was chosen. A random-effects meta-analysis was used to provide an overall summary measure of effect (mean difference in minutes/day of sedentary time) and 95% CIs for each meta-analysis. Self-report estimates that on average were within 30 minutes of the device measure were considered to have appropriate agreement. Thirty minutes has been identified as clinically meaningful for having an effect on cardiometabolic health [31–33]. *A priori* determined subgroup analyses were carried out to test differences for: males vs. females, multi vs. single item questionnaires, week/workday vs. weekend/non-workday recall, work vs. non-work time, questionnaire comparisons (e.g., IPAQ vs. GPAQ vs. SBQ vs. log vs. diary vs. EMA, etc.), healthy vs. chronic (e.g., diabetes, cardiovascular disease, pulmonary disease, cancer, osteoporosis, arthritis, lupus, fibromyalgia, intellectual disabilities, mental illness, depression, people who are deaf or blind, back pain) populations, and validation against accelerometer vs. inclinometer. If a study only provided sex-stratified results, the individual male and female data were included in the meta-analyses separately, but if a total combined estimate was provided this was used rather than sex-specific estimates (except in the sex-stratified sub-group analysis). Publication bias was conducted using examination of funnel plots for symmetry. Post-hoc sensitivity analyses also examined comparisons in overweight/obese and pregnant populations, by wear location (e.g., hip/waist, wrist, arm, lower back), and accelerometer cut-point.

### Risk of bias

We assessed the quality of the individual studies used in the meta-analyses using a modified version of the QUADAS2 tool used to examine bias in studies of diagnostic accuracy [34]. In the context of this review, the device measure was considered the ‘gold standard’. Studies were assessed for potential biases including: selection bias (sampling methods); index test bias [conduct or interpretation of the self-report measure including blinding of device output, continuous vs. categorical measures (continuous being better with an upper threshold), clear definition/question]; reference standard bias (conduct or interpretation of the device measure including the use of validated intensity cut-points if applicable and interpretation without a self-report measure), and flow and timing (measures capture the same time period, all participants received same devices). Individual risk of bias information was combined to provide an assessment of overall quality of the evidence. Risk of bias assessments were carried out by one assessor (SAP) and verified by a second (LC, JLR, TJS, CK, KD, or JPB).

## Results

### Study characteristics

**Fig. 1:**
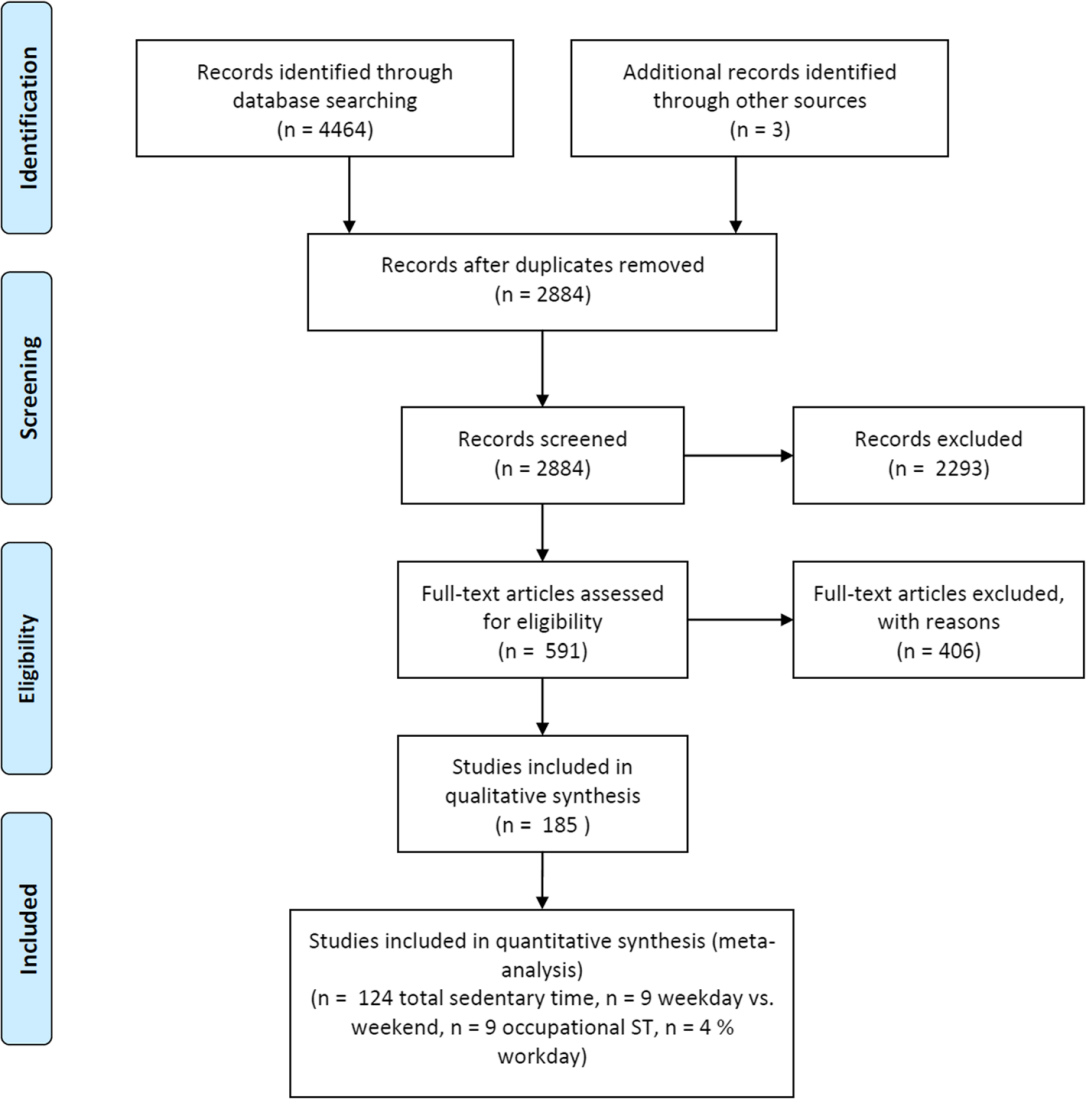
PRISMA flow diagram

Figure 1 provides a detailed flow diagram of the literature search and screening process. The preliminary search of the electronic databases identified 4,464 potentially relevant papers. Of these, 1,089 were identified in MEDLINE, 1,313 in EMBASE, 271 in PsycINFO, 266 in SPORTDiscus, 1,240 in CINAHL, and 285 in Dissertations and Theses Global. After de-duplication, 2,881 relevant papers remained. A preliminary title and abstract review resulted in the retrieval of 591 full text papers for a detailed assessment. Author’s knowledge and bibliographies identified a further three papers. Of these, 185 unique studies met the criteria for study inclusion [19, 20, 24–30, 35–210]. Individual study characteristics can be seen in Supplemental table 2. Common reasons for excluding studies included: no measure of SB (n = 114); no comparisons of measures (n = 80); not adult population (n = 5); no self-report measure (n = 66); no device measure (n = 46); measures not comparable (n = 23); no measure of variance (n = 6); duplicate study (n = 31); review paper (n = 15); not English or French (n = 14); study protocol with no results (n = 6); and, unable to obtain full text (n = 3).

The included studies were published from 2004 to 2019; the majority from the past five years. A total of 36 countries were represented by at least one study. The countries with the most studies included the United States (29%) and Australia (15%). A greater number of studies reported on female-specific data/populations compared to male-only data. The IPAQ-SF (37 studies), IPAQ-LF (32 studies), GPAQ (17 studies), SBQ (13 studies), and the Occupational Sitting and Physical Activity Questionnaire (OSPAQ; 8 studies) were the most common self-report measures and the ActiGraph GT3X the most used device measure followed by the activPAL. All but six of the studies [42, 91, 101, 102, 122, 205] that employed inclinometers used activPALs. Accelerometers were largely worn on the hip (some reported wear at the wrist, arm or thigh) whereas inclinometers were worn exclusively on the thigh. Devices used to objectively measure sedentary time included: accelerometers, inclinometers, combined camera + accelerometers, combined heart rate + activity monitors, television monitors, and car monitors.

### Risk of bias

**Fig. 2:**
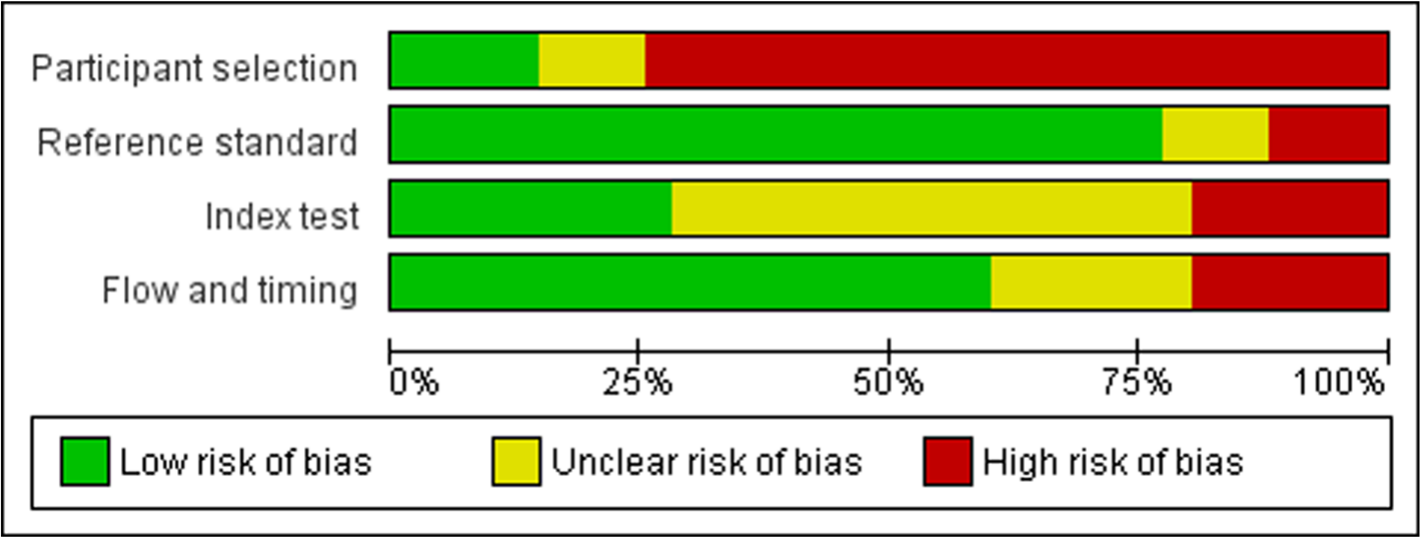
Summary risk of bias graph

Risk of bias results are summarized in Fig. 2, individual study risk of bias results can be seen in the respective forest plots. Seventy-five percent of the studies had a high risk of selection bias largely due to the use of convenience samples or including populations that were specialized (thereby limiting the generalizability of their findings to the general population). The majority (80%) of studies had a low risk of bias related to the reference standard (device) as most used validated thresholds to define SB or used inclinometry. Approximately 50% had inadequate information to assess the quality of the index test as most failed to mention whether an upper threshold was instituted on continuous reporting outcomes, while approximately 30% had a low risk of bias related to the self-report measure. While the majority (60%) of studies had a low risk of bias related to the flow and timing of measures, ~20% had a mismatch between the time period of data collection by the self-report and device measures. In the conduct of this review, flow and timing was considered the most critical form of bias. There was, however, no clear trend in the degree of agreement between the two measures based on this bias.

#### Validity

Supplemental table 3 provides study findings for all studies comparing a self-report and device measure of SB. A total of 147 studies reported a correlation coefficient between a self-report and device measure of sedentary time. Supplemental figure 1 is a plot of all the extracted correlations and shows, that on average, the correlations between self-report and device measures was low-to-moderate at r = 0.32 (SD = 0.21) and ranged from -0.19 to 0.87. Negative correlations would imply that as self-report values are higher while device-based measures are lower.

A total of 123 studies comprising 173 comparisons and data from 55,199 participants were used to examine the criterion validity of self-reported average sedentary or sitting time by comparison to a device measure of sitting or sedentary time [24–30, 35–40, 42, 43, 47, 48, 50–56, 58, 62–64, 66–68, 73, 74, 76–82, 84–86, 88–90, 92, 94–100, 102–104, 107–109, 111–113, 116, 117, 120, 121, 124–126, 128, 129, 131–133, 135, 138–140, 142–145, 147, 148, 151, 153, 156, 157, 160, 161, 163–165, 168, 170, 171, 173–177, 180, 182–184, 186–189, 191, 194–196, 198–200, 202, 203, 206, 208–210]. The average mean difference (MD) between measures was -105.19 minutes/day (95% CI: -127.21, -83.17) indicating that self-report tools underestimated sedentary time by approximately 1.74 hours/day compared to device measures (Supplemental figure 2). Heterogeneity was high (I^2^ = 99%) indicating a great deal of variation between studies with as much as 6 hours/day discrepancy within individual studies. There was no clear pattern in under- or over-reporting based on the risk of bias associated with timing and flow. The funnel plot was largely symmetrical and there was no apparent publication bias.

Additional studies examined and compared the criterion validity of self-report measures under the following conditions: nine studies (19 comparisons) on weekday/workday (includes time at work and outside of work) vs. weekend/non-workday sedentary time [24, 47, 51, 58, 69, 70, 86, 156, 198]; 2 studies (5 comparisons) on minutes per day of self-reported television time [141, 155]; nine studies (11 comparisons) on minutes per day of occupational sedentary time [61, 65, 75, 99, 101, 105, 122, 136, 181, 188, 193]; and, four studies (five comparisons) on the proportion of a workday spent sedentary [91, 105, 158, 205]. In all of these studies a comparable criterion was required (e.g., self-reported occupational sitting was compared to accelerometer sedentary time during work time only). Self-reported weekday/workday (MD = -77.60, 95% CI: -121.36, -33.84, I^2^ = 93%) and weekend/non-workday (MD = -87.92, 95% CI: -149.15, -26.69, I^2^ = 95%) sedentary time were both significantly different than device-assessed sedentary time, but were not significantly different from each other (Supplemental figure 3, χ ^2^ = 0.07, df = 1, p = 0.79). Self-reported television time was significantly lower than television time derived from a combination of logs and accelerometers or television monitoring devices (Supplemental figure 4, MD = -31.82 minutes/day, 95% CI: -49.36, -14.29, I^2^ = 0%). On average, self-reported time spent sedentary at work was significantly greater than that assessed by device (regardless of device) (Supplemental figure 5, MD = +37.81 minutes/day, 95% CI: 22.41, 53.21, I^2^ = 50%). Self-reported proportion of a workday spent sedentary was not significantly different from the inclinometer-assessed proportion (Supplemental figure 6, MD = +2.25%, 95% CI: -2.42%, 6.93%, I^2^ = 66%).

Subgroup analyses found that males (MD = -130.38, 95% CI: -182.34, -78.41, I^2^ = 98%) and females (MD = -143.75, 95% CI: -189.93, -97.57, I^2^ = 99%) under-reported their sedentary time by a similar amount when compared to device measures (Supplemental figure 7, χ^2^ = 0.14, df = 1, p = 0.71). Examining only those studies which provided both male- and female-specific data did not change the findings (χ^2^ = 0.01, df = 1, p = 0.91).

No significant between group differences in the discrepancy between self-report and device measures were observed for participants from apparently healthy populations, chronic populations, pregnant women or those who were considered overweight or obese (Supplemental figure 8, χ^2^ = 3.18, df = 3, p = 0.36, I^2^ = 5.6%). Those from apparently healthy populations (MD = -89.96, 95% CI: -114.45, -65.46, I^2^ = 99%), chronic populations (MD = -154.01, 95% CI: -223.98, -84.04, I^2^ = 99%), and those who were considered overweight or obese (MD = -104.59, 95% CI: -197.97, -11.20, I^2^ = 99%) all under-reported their sedentary time compared to the device measure. Among the three studies that included pregnant women, self-reports were not significantly different than device measures, though they trended toward under-reporting (MD = -74.16, 95% CI: -156.09, 7.77, I^2^ = 90%). Chronic populations appear to under-report to a greater degree than apparently healthy populations (χ^2^ = 2.87, df = 1, p = 0.09, I^2^ = 65.1%).

**Fig. 3:**
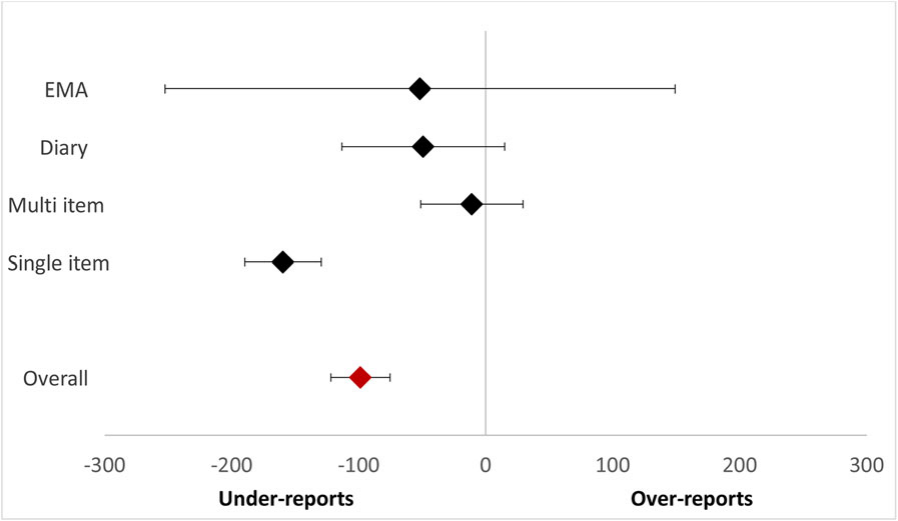
Summary forest plot comparing self-report and device measures of total sedentary or sitting time between single vs. multi-item vs. EMAs vs. diaries/logs, minutes/day

**Fig. 4:**
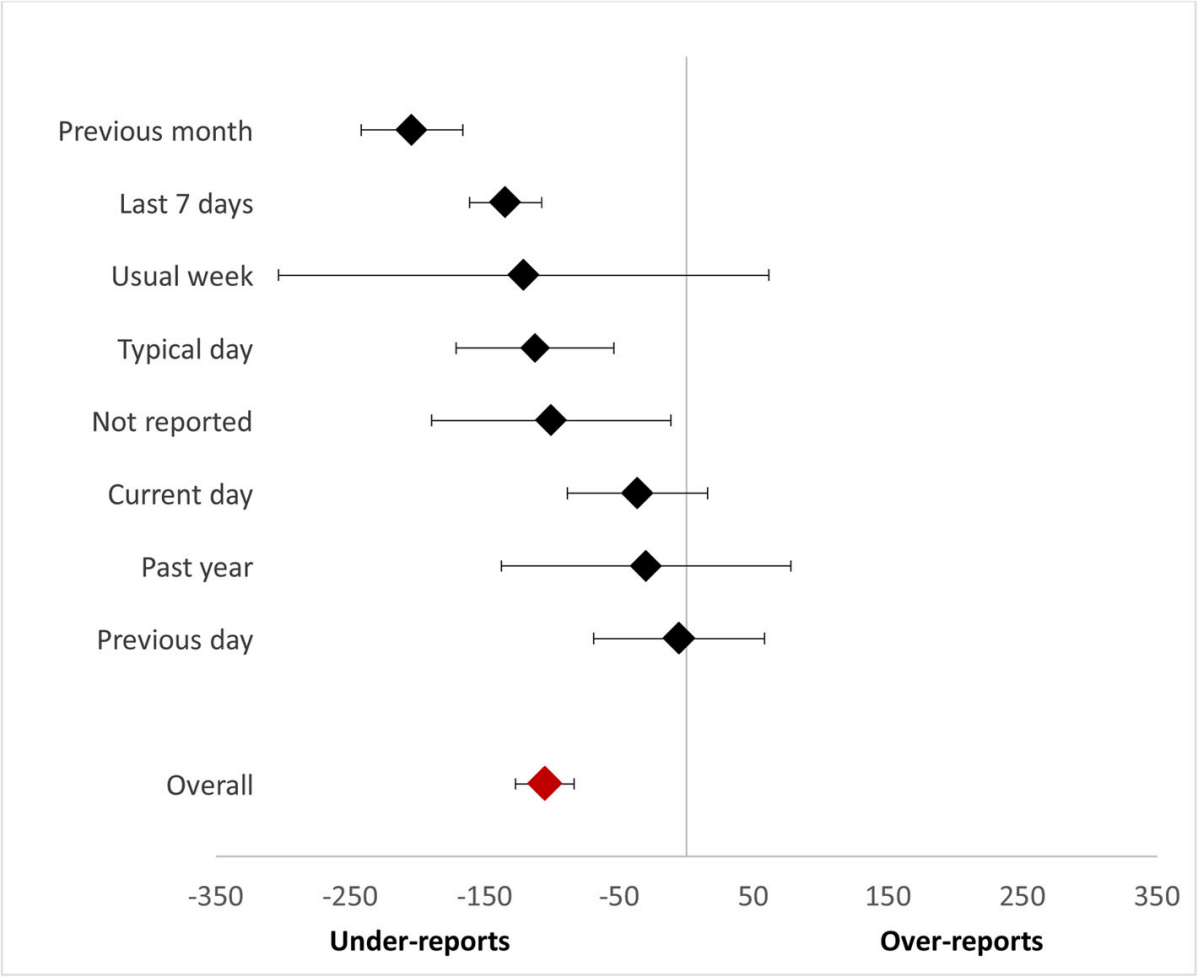
Summary forest plot comparing self-report and device measures of total sedentary time or sitting time across self-report recall periods

Subgroup comparisons were used to test the performance of single sedentary questions (e.g., IPAQ-LF [only sitting questions], IPAQ-SF, GPAQ) compared to multi-item/component questionnaires (excluding IPAQ + motorized travel), EMAs, and logs/diaries. A significant between group difference was found (Fig. 3, χ^2^ = 36.51, df = 3, p<.00001, I^2^ = 91.8%) whereby single item measures, on average, significantly under-reported sedentary time compared to device measures (MD = -159.56, 95% CI: -189.69, -129.44, I^2^ = 99%), whereas multi-item questionnaires (MD = -10.93, 95% CI: -51.13, 29.28, I^2^ = 99%), EMAs (MD = -51.56, 95% CI: -252.33, 149.21, I^2^ = 98%) and log/diaries (MD = -49.10, 95% CI: -113.08, 14.88, I^2^ = 92%) did not. Almost all (82%) of the single item studies significantly under-reported compared to 37% of multi-item questionnaires and 22% of logs/diaries. A sub-group analysis was also performed to examine the effect of recall duration on self-report measure performance. Current day (MD = -36.14, 95% CI: -88.30, 16.02) and previous day (-5.22, 95% CI: -68.76, 58.31) recalls performed the best, while recalls over the previous seven days (MD = -134.39, 95% CI: -161.14, -107.64), previous month (MD = -204.04, 95% CI: -241.64, -166.43), usual day (MD = -112.49, 95% CI: -171.09, -53.89), and usual week (MD = -121.01, 95% CI: -303.36, 61.34) performed the worst (Fig. 4, Supplemental figure 9). Almost all previous week recalls underestimated sedentary time, while previous day was more evenly split between under- and over-estimation and current day had the smallest mean differences within the individual studies.

Within questionnaires there was a significant between group difference (Supplemental figure 10, χ^2^ = 121.98, df = 12, p < 0.00001, I^2^ = 90.2%). The IPAQ-Short Form (MD = -161.67, 95% CI: -226.38, -96.95, I^2^ = 99%), IPAQ-Long Form sitting questions (MD = -150.76, 95% CI: -193.68, -107.83, I^2^ = 99%), IPAQ-Long Form sitting + motorized travel (MD = -271.67 (-397.68, -145.36, I^2^ = 99%), and GPAQ (MD = -219.85, 95% CI: -288.68, -151.02, I^2^ = 98%) had the greatest discrepancy between self-reported sitting time and device-assessed sedentary time. The SBQ performed the best with an average mean difference of ~6 minutes/day (MD = -5.80, 95% CI: -73.48, 61.87, I^2^ = 97%). However, the SBQ studies had a high level of heterogeneity and were split with 4/9 significantly under-reporting and 3/9 significantly over-reporting, whereas the IPAQ and GPAQ consistently under-reported sitting time. On average, the Multimedia Activity Recall for Children and Adolescents (MARCA) questionnaire (MD = 56.56, 95% CI: 22.61, 90.52, I^2^ = 13%) and the sitting time questionnaire developed by Marshall [134] (MD = 83.85, 95% CI: -1.00, 168.71, I^2^ = 89%) over-reported sedentary time compared to device measures.

A sub-group analysis identified that the difference between self-report and device measures varied depending on the type of device used (Supplemental figure 11, χ^2^ = 25.99, df = 2, p < 0.00001, I^2^ = 92.3%). A significant difference between accelerometer (MD = -125.48, 95% CI: -151.58, -99.39, I^2^ = 99%) and a combined accelerometer + heart rate monitor (MD = -157.68, 95% CI: -209.35, -106.01, I^2^ = 97%) and self-report measures was observed. An analysis of different models of accelerometers (i.e., ActiGraph 7164, ActiGraph GT1M, ActiGraph GT3X, SenseWear) revealed no between group differences (χ^2^ = 3.64, df = 3, p = 0.30, I^2^ = 17.6%). Further, when comparing accelerometer cut-points (e.g., 50 cpm x-axis, 100 cpm x-axis, 100 cpm vector magnitude, 150 cpm vector magnitude) against a single-item measure (e.g., IPAQ, GPAQ) there were no between group differences (Supplemental figure 12, χ^2^ = 5.23, df = 3, p = 0.16, I^2^ = 42.7%). The majority of accelerometers with wear location reported were worn on the dominant hip or waist. A sensitivity analysis of wear location found that the self-report measures were significantly lower when compared to wrist vs. waist/hip locations (Supplemental figure 13, χ^2^ = 21.35, df = 3, p < 0.00001, I^2^ = 85.9%). On average, self-report measures when compared to monitors worn on the upper arm (e.g. SenseWear Armband) were not significantly different (MD = -81.74, 95% CI: -174.53, 11.04, I^2^ = 99%), though there was a high degree of variation between and within studies (up to almost 4 hours/day), and most self-report measures were multi-component or diaries/logs. Contrary to the accelerometer results, no significant differences were observed when comparing self-report measures to an inclinometer (MD = -10.55, 95% CI: -52.30, 31.19, I^2^ = 98%). However, when only studies that included both an accelerometer and inclinometer (e.g., Actigraph + activPAL) outcome compared to a self-report measure were used, no significant difference between the self-report and device measure were observed (χ^2^ = 0.30, df = 1, p = 0.58, I^2^ = 0%) when comparing activPAL (MD = 6.86, 95% CI: -46.78, 60.49) and accelerometers (MD = -13.06, 95% CI: -60.26, 34.15). A sensitivity analysis found that self-reported sedentary time using single-item questionnaires (including IPAQ-LF without motorized transport) was significantly lower when compared to both inclinometers (MD = -127.29, 95% CI: -213.85, -40.72) and accelerometers (MD = -156.84, 95% CI: -190.04, -123.64) (χ^2^ = 0.39, df = 1, p = 0.53, I^2^ = 0%). However, when multi-item questionnaires (including IPAQ-LF + motorized transport), logs/diaries and EMAs were compared to device measures, they performed better when compared to inclinometers (MD = 22.59, 95% CI: -23.18, 68.35) versus accelerometers (MD = -62.18, 95% CI: -105.77, -18.59) (χ^2^ = 6.91, df = 1, p = 0.009, I^2^ = 85.5%).

#### Reliability

**Fig. 5:**
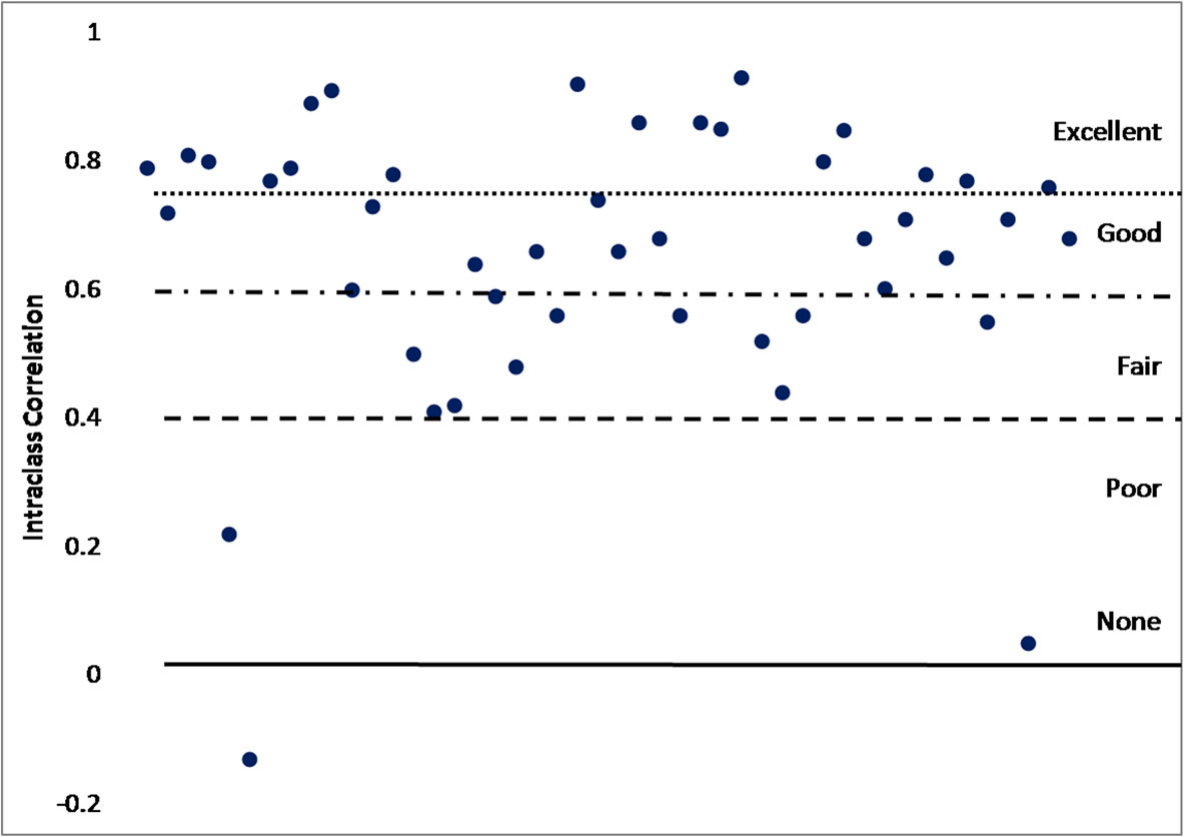
Intraclass correlation coefficients between repeated self-report measures

A total of 54 studies provided an assessment of reliability of a self-report measure (Supplemental table 4). The intraclass correlation coefficient (ICC) was the most reported reliability statistic (46 studies) and is a measure of test-retest reliability used to assess whether similar estimates of the outcome (i.e., SB) were obtained across multiple assessments. ICC values range from 0 to 1; where 1 indicates perfect reliability and 0 represents a lack of reliability. In this review we considered an ICC over 0.75 to be excellent, between 0.60 and 0.74 to be good, between 0.40 and 0.59 to be fair, and < 0.40 to be poor [211]. Figure 5 is a plot of all the extracted ICCs and shows that on average, the reliability of the self-report measures was good with ICC = 0.66 (SD = 0.22) and ranged from -0.13 to 0.91.

## Discussion

To our knowledge, this review represents the most comprehensive comparison to date of self-report and device-assessed SB measures in adults. Contrary to what was previously found in the PA literature [9, 13], the majority of the studies included in this review reported on units that were comparable between the two measures and reported measures of variance around estimates. This allowed us to conduct large meta-analyses to compare self-report and device measures and to perform a series of sub-group analyses.

On average, we found that self-report measures under-reported sedentary/sitting time when compared to device measures by 1.74 hours/day (95% CI: -2.11, -1.38 hours/day) with 72% of comparisons identifying that self-reports under-reported compared to device measures. Single question measures (e.g., IPAQ, GPAQ) resulted in significant under-reporting of sedentary time compared to all device measures, whereas multi-item questionnaires, EMAs, and logs/diaries did not, and performed relatively well, especially when compared to activPALs (the gold standard of field-based sitting). Therefore, it appears that the use of multi-item questionnaires, logs/diaries and EMAs may provide more comparable results to device measures versus single-item questions. There was, however, a large and significant degree of variability within (up to 4.5 hours/day) and between studies, and this was present in most of the meta-analyses performed. In the case of diaries/logs and EMAs where fewer studies were used in the meta-analysis, this variability further limits our confidence in the reproducibility of the results. While most led to an under-reporting (though to different degrees), some provided similar estimates and some over-reported and the 95% CIs around these estimates were often quite large with the majority greater than 30 minutes. This variability has great consequences for the interpretation and use of measures and is something that users should consider when deciding on which tool to use. For example, while multi-item questionnaires, EMAs and logs/diaries had more acceptable mean differences, the variability was quite large and not generally acceptable (up to 1.8 hours/day). It is also important to recognize that most studies that employed a single item did so using the global sitting question from the IPAQ or the GPAQ (or based on these questions). The sitting time question is part of a larger questionnaire on PA and was developed for surveillance purposes, whereas multi-item SB questionnaires such as the SBQ and WSQ were designed as standalone surveys to obtain more detailed estimates of SB.

Self-reported work-time spent sedentary appeared to be better recalled compared to total day sedentary time. Interestingly, questionnaires that asked participants to recall the proportion of their workday spent sedentary did not significantly differ from inclinometer-assessed sedentary time ranging from an average over-reporting of 9% to under-reporting by 4%. Chastin et al., compared 18 self-report measures of sedentary time against an activPAL and found tools that used a scale to assess the proportion of daily time spent sitting had the lowest amount of missing data and best agreement with device-assessed measures [25]. Matsuo et al. also found that when Japanese workers were asked to indicate the percentage of time spent sitting at work it improved the reliability and validity of the Workers’ Sitting and Walking-time Questionnaire (WSWQ) compared to asking them to recall length of time [136]. Asking individuals to recall the proportion of their day spent sitting rather than recalling absolute time appears to provide more accurate measures of sitting especially in the context of workplace sitting. Future studies would benefit from assessing this method using a total-day approach.

It is important to remember that self-report and device measures provide different and complimentary information [10, 212]. As described by Colley et al. (2018), self-report measures provide a measure of the behaviour as perceived and thereby estimated by the respondent and can provide more contextual information such as the type of SB being performed (e.g., television watching, passive transport, screen time) whereas device measures continuously capture bodily movement at specific thresholds [213] or in the case of inclinometers time spent in specific postures (i.e. sitting/lying), but often lack contextual information except in the case of wearable cameras or user monitor devices (e.g., television, computer and car use monitors).

EMAs, logs/diaries and previous day recalls generally performed better than single item questionnaires which ask participants to recall total sitting in the past week/month/year or on a usual day as they remove or attenuate potential recall and response biases by capturing self-report closer to ‘real-time’ and often assess details about body position and different types of behaviours. They also provide an upper threshold for reporting by asking respondents to reflect upon smaller portions of time. Similarly to what was observed by Chastin et al., multi-item questionnaires performed better than single-item questions for accuracy, but with both having poor precision (large 95% CIs) [25]. This may be attributed to asking respondents to recall specific sedentary activities/behaviours (i.e., television watching, time spent commuting) thereby providing cues to recall behaviours which may not be otherwise considered or which may occur over smaller periods of time rather than estimating how much time one spends sitting throughout a day. Self-reported occupational sedentary time also tended to be over-reported, but performed better than total sitting measures. It may be easier for respondents to recall time spent sitting at work or during a task than recalling time over the course of the day.

Contrary to what has been observed in the literature for PA [9], there does not appear to be gender differences in how men and women report their sedentary time compared to device measures. Further, findings were similar between general/apparently healthy and chronic (e.g., presence of disease or condition) populations improving the generalizability of the results. Interestingly, Chastin et al. [25] compared 18 different combinations of self-report measures in the same population and identified large differences in the performance of the measures. This evidence also supports that differences between studies are not necessarily attributed to differences in populations. Findings were, however, different depending on the device used for comparisons. Comparisons made with accelerometers or combined accelerometer + heart rate monitors showed lower accuracy compared to inclinometers (e.g., activPALs), but all had a great degree of variability within and between studies (up to 6 hours/day compared to accelerometers and up to 4.6 hours/day compared to inclinometers). This was especially true when looking at differences in multi-item questionnaires, EMAs and logs/diaries. Inclinometers (especially activPALs) are considered a “gold standard for the objective measurement of sedentary/sitting time” [25] and are recommended for assessing SB in the field [22]. Inclinometers assess time spent in specific postures (i.e., sitting/lying) by using acceleration to measure the inclination of the thigh (thigh wear is the most valid for posture) relative to gravity, the combination of movement intensity and posture align with the definition of SB [3]. Accelerometers generally apply a movement threshold (most notably <100 cpm) to define time spent sedentary. It is therefore, possible that these thresholds identify other behaviours (i.e. standing stationary) that would not be considered sedentary activities and may explain the greater discordance between self-report and accelerometer measures. Further, although our comparison of accelerometer cut-points against single-item questions showed no significant differences, different thresholds have been shown to capture different amounts of SB [22, 214] with the potential to affect the comparability between measures.

Research has shown that self-report and device-based measures of SB associate differently with health outcomes [53, 163, 215, 216]; device-assessed SB has been shown to associate more strongly with mortality compared to self-reported sitting [216, 217]. It also appears that health risks associate differently or have a stronger relationship with different types and domains of self-reported SB [4, 218]. For example, greater self-reported television has been shown to be more strongly associated with mortality than self-reported total sitting [4]. This may be due in part to television’s association with unhealthy food and beverage consumption and sleep [219, 220] or to our ability to more accurately recall this specific behaviour, but future work is needed to better understand why different types of SB and why self-report and device measures associate differently with health outcomes.

Although correlation between self-report and device measures was low-to-moderate, self-report measures had an acceptable level of reliability. Reliability is important when assessing SB over time whether to assess changes from an intervention or to monitor prevalence in a population [10].

### Limitations

This review has limitations that should be considered when extrapolating results. First, the review was limited to studies that included directly comparable SB data between the self-report and device measures. This greatly reduced the number of studies examining self-reported activities such as television and passive transit which are difficult to compare to device measures of the same behaviour. However, it does provide an assessment of questionnaires that included multiple SBs and many that examine self-reported sitting. It should be noted that almost all the meta-analyses demonstrated a high degree of heterogeneity between and within (up to 6 hours/day) studies, limiting our confidence in the estimates. However, given the large number of included studies, we feel that the high degree of heterogeneity is likely, at least in part, explained by the potential variability in recall and response bias within study populations. While we did not identify any difference in healthy vs. chronic populations, there may be other respondent-level factors at play. Within studies that assessed television viewing, we cannot be certain that individuals were undertaking non-sedentary behaviours while the television was on.

## Conclusions

This review presents a comprehensive examination of self-report measures of SB and how they compare to device measures. The field of SB has experienced a rapid rate in growth over the last 5-10 years and with this growth has come an evolution of measurement methods. While devices have become increasingly used for assessing these behaviours, they provide limited contextual information to explain where and how individuals are sedentary. Self-report measures continue to be the most widely used methods to assess these behaviours. Evidence from this review suggests that self-report single-item measures generally underestimate sedentary time when compared to device measures. Multi-items questionnaires, EMAs and logs/diaries and tools that employ a shorter recall period perform well with respect to accuracy, especially when compared to inclinometers, but present with a high degree of variability (many over- and under- reporting) within and between studies leading to poor precision. This variability, which in individual studies could be as much as six hours/day, has great consequences for the interpretation and use of measures. It is apparent that there is an abundance of self-report tools available for researchers, making it difficult to compare findings across studies. Researchers should exert caution when comparing associations between different self-report and device measures with health outcomes.

## Supplementary information

**Additional file 1: Supplemental figure 1.** Correlation coefficients between self-report and device measures.

**Additional file 2: Supplemental figure 2.** Forest plot comparing self- 841 report and device measures of total sedentary or sitting time, minutes/day.

**Additional file 3: Supplemental figure 3.** Forest plot comparing self- 843 report and device measures of total sedentary or sitting time between 844 weekday/work days and weekend/non-work days, minutes/day.

**Additional file 4: Supplemental figure 4.** Forest plot comparing self- 846 report and device measures of television viewing time, minutes/day.

**Additional file 5: Supplemental figure 5.** Forest plot comparing self-report and device measures of total occupational sedentary time, minutes/day.

**Additional file 6: Supplemental figure 6.** Forest plot comparing self-report and device measures of workday spent sedentary, % of day.

**Additional file 7: Supplemental figure 7.** Forest plot comparing self-report and device measures of total sedentary or sitting time between men and women, minutes/day.

**Additional file 8: Supplemental figure 9.** Forest plot comparing self-report and device measures of total sedentary or sitting time between recall periods, minutes/day.

**Additional file 9: Supplemental figure 8.** Forest plot comparing self-report and device measures of total sedentary or sitting time between population subgroups, minutes/day.

**Additional file 10: Supplemental figure 10.** Forest plot comparing self-report and device measures of total sedentary or sitting time across questionnaires, minutes/day.

**Additional file 11: Supplemental figure 11.** Forest plot comparing self-report and device measures of total sedentary or sitting time between devices, minutes/day.

**Additional file 12: Supplemental figure 12.** Forest plot comparing self-report and accelerometer measures of total sedentary or sitting time across cut-points, minutes/day.

**Additional file 13: Supplemental figure 13.** Forest plot comparing self-report and device measures of total sedentary or sitting time across wear locations, minutes/day.

**Additional file 14: Supplemental table 1.** Ovid MEDLINE search strategy.

**Additional file 15: Supplemental table 2.** Study characteristics.

**Additional file 16: Supplemental table 3.** Validity characteristics.

**Additional file 17: Supplemental table 4.** Reliability characteristics.

## Data Availability

All data generated or analysed during this study are included in this published article [and its additional files].
