# Supplementary figures and images for: A comparison of self-reported and device measured sedentary behaviour in adults: a systematic review and meta-analysis
Source: Int J Behav Nutr Phys Act. 2020 Mar 4;17:31. doi: 10.1186/s12966-020-00938-3 (PMC7055033; doi:10.1186/s12966-020-00938-3)

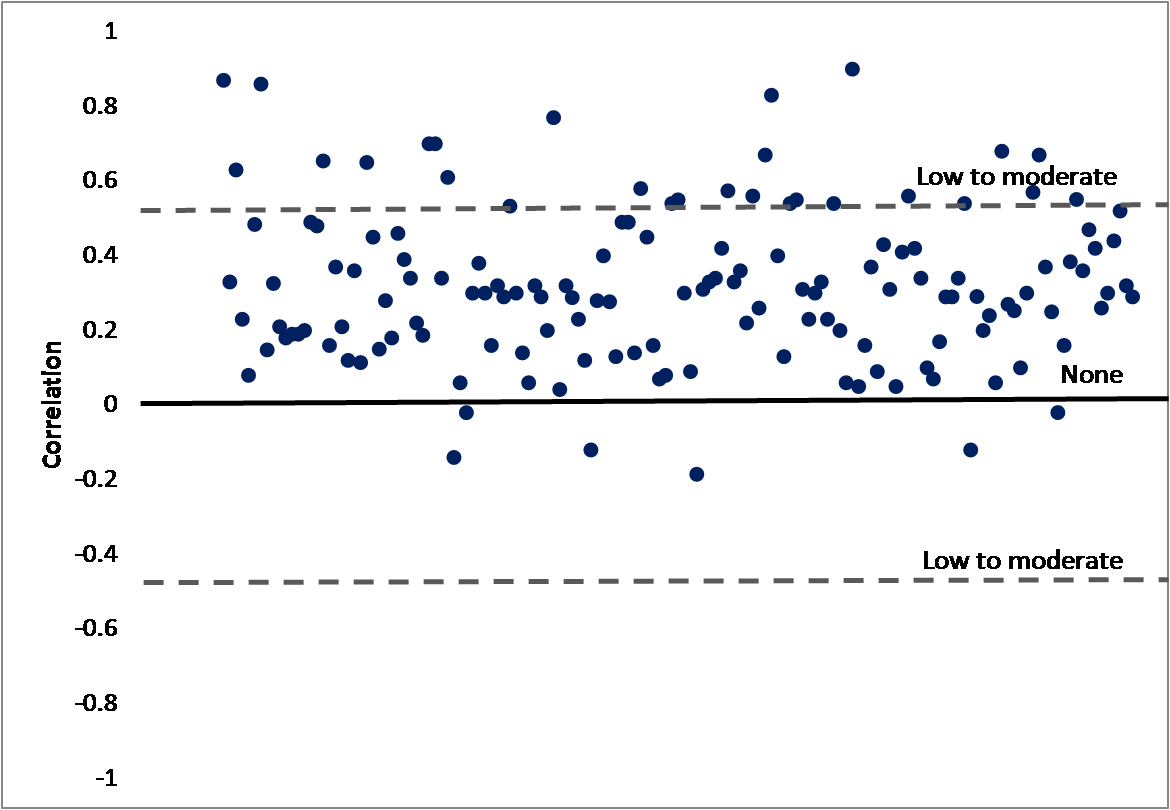

Supplement: Supplementary file 1 — Additional file 1: Supplemental figure 1. Correlation coefficients between self-report and device measures. [file 12966_2020_938_MOESM1_ESM.png]

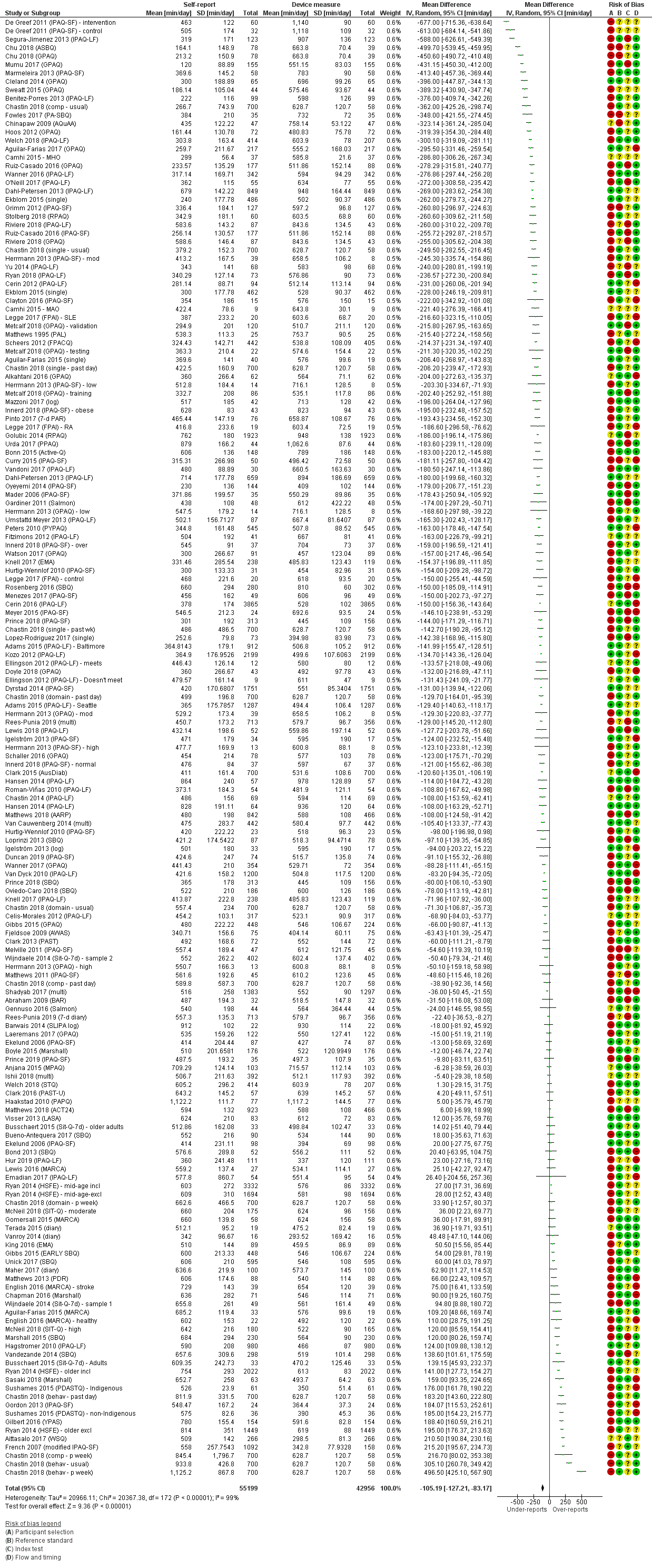

Supplement: Supplementary file 2 — Additional file 2: Supplemental figure 2. Forest plot comparing self- 841 report and device measures of total sedentary or sitting time, minutes/day. [file 12966_2020_938_MOESM2_ESM.png]

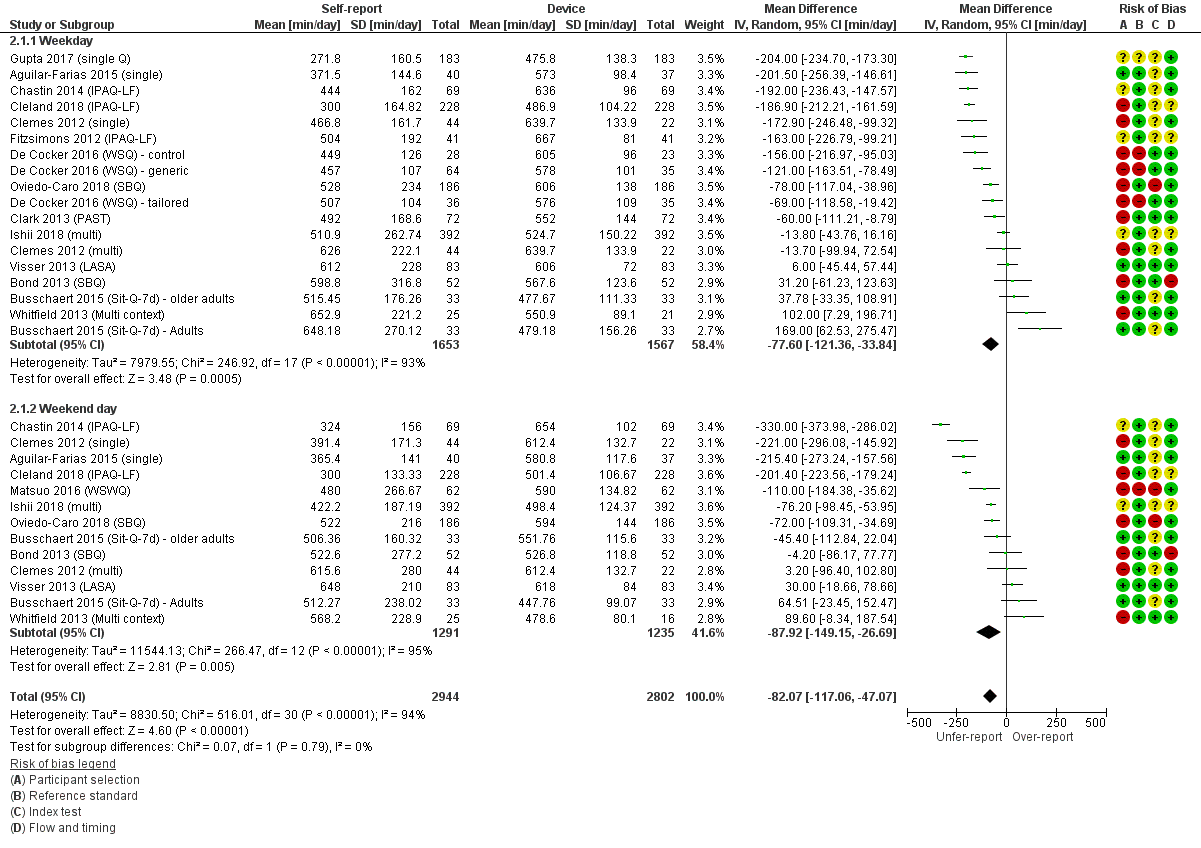

Supplement: Supplementary file 3 — Additional file 3: Supplemental figure 3. Forest plot comparing self- 843 report and device measures of total sedentary or sitting time between 844 weekday/work days and weekend/non-work days, minutes/day. [file 12966_2020_938_MOESM3_ESM.png]

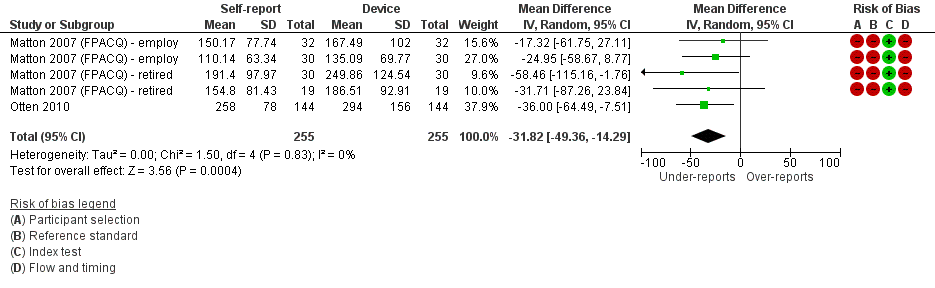

Supplement: Supplementary file 4 — Additional file 4: Supplemental figure 4. Forest plot comparing self- 846 report and device measures of television viewing time, minutes/day. [file 12966_2020_938_MOESM4_ESM.png]

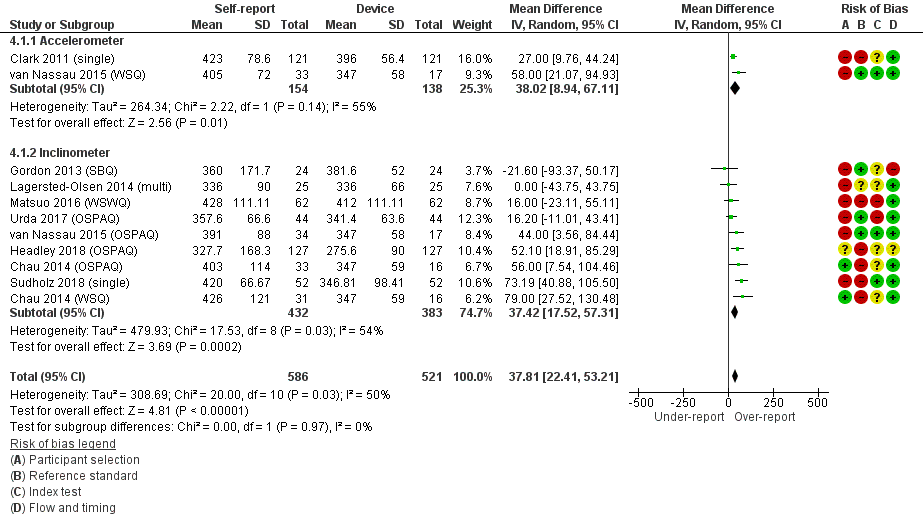

Supplement: Supplementary file 5 — Additional file 5: Supplemental figure 5. Forest plot comparing self-report and device measures of total occupational sedentary time, minutes/day. [file 12966_2020_938_MOESM5_ESM.png]

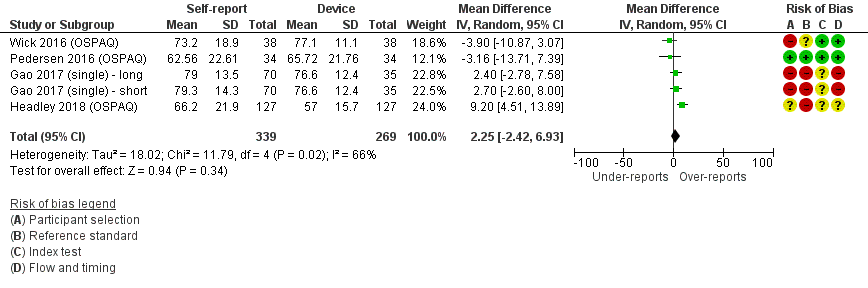

Supplement: Supplementary file 6 — Additional file 6: Supplemental figure 6. Forest plot comparing self-report and device measures of workday spent sedentary, % of day. [file 12966_2020_938_MOESM6_ESM.png]

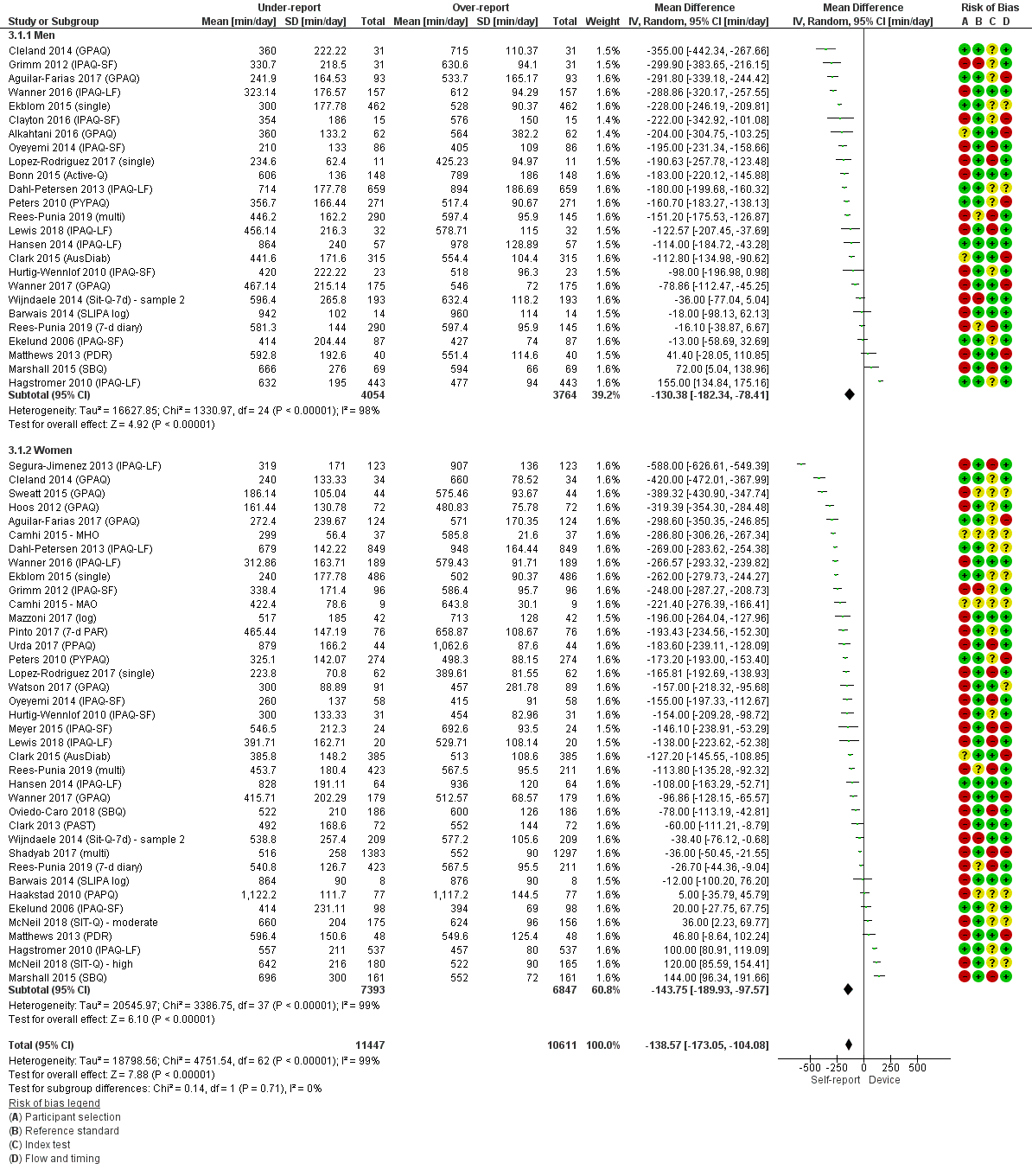

Supplement: Supplementary file 7 — Additional file 7: Supplemental figure 7. Forest plot comparing self-report and device measures of total sedentary or sitting time between men and women, minutes/day. [file 12966_2020_938_MOESM7_ESM.png]

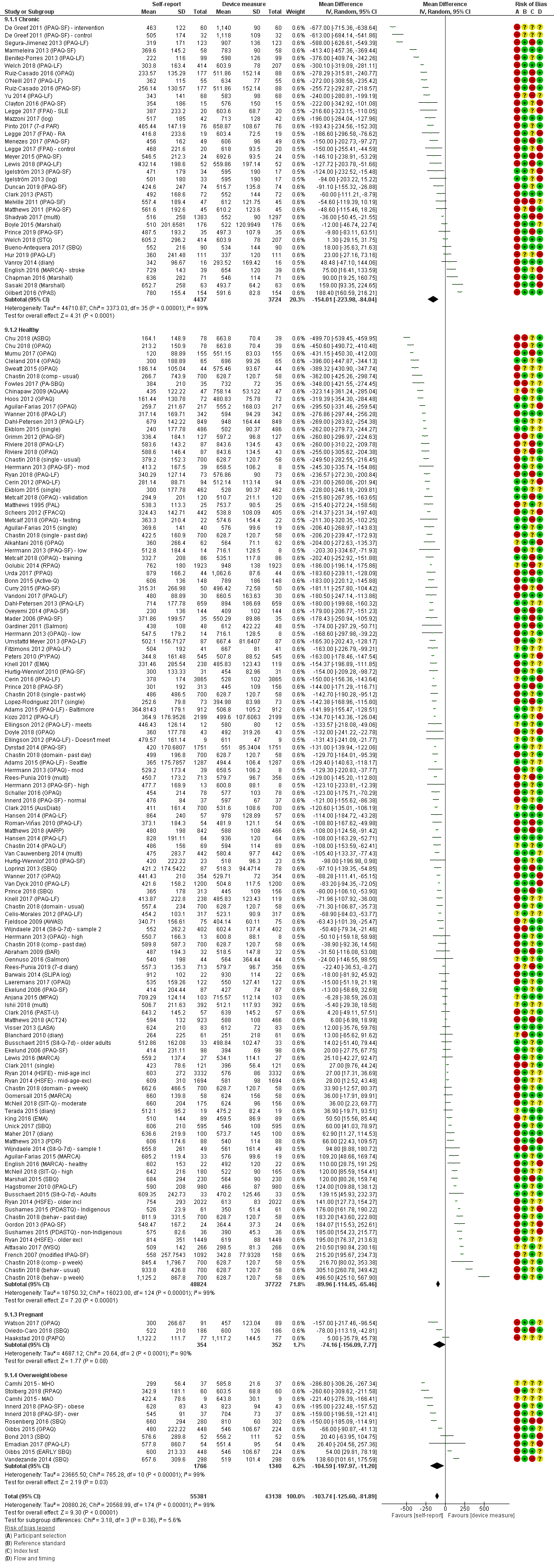

Supplement: Supplementary file 8 — Additional file 8: Supplemental figure 9. Forest plot comparing self-report and device measures of total sedentary or sitting time between recall periods, minutes/day. [file 12966_2020_938_MOESM8_ESM.png]

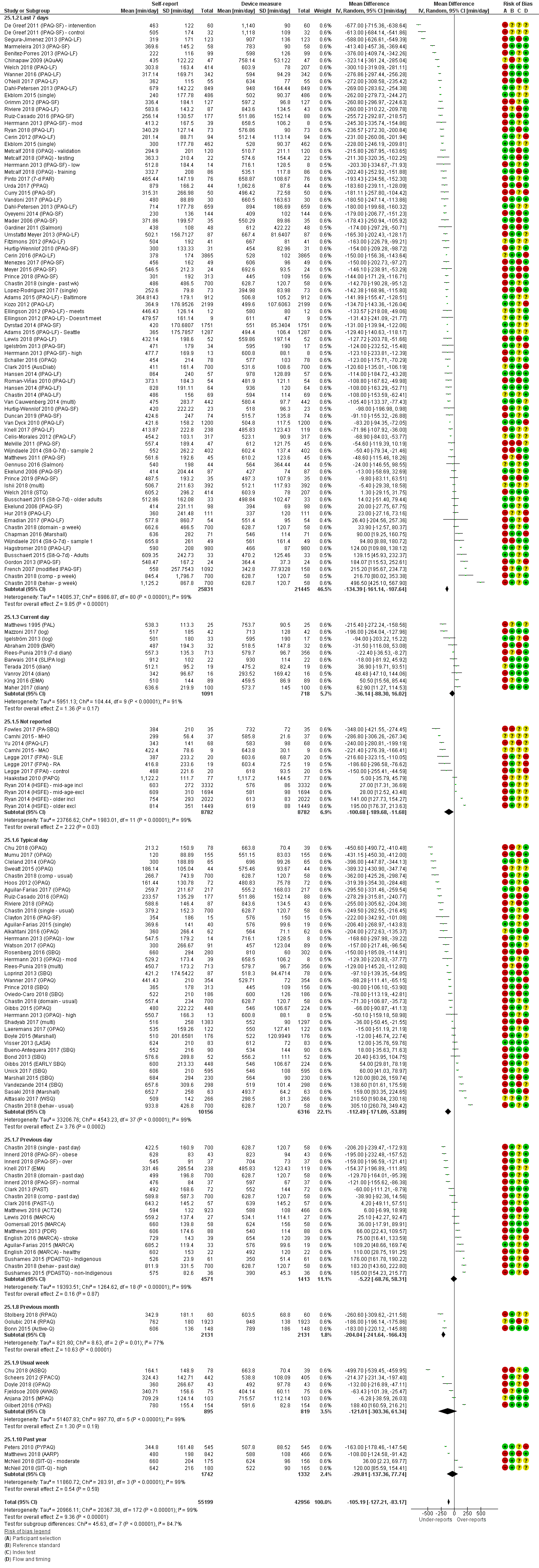

Supplement: Supplementary file 9 — Additional file 9: Supplemental figure 8. Forest plot comparing self-report and device measures of total sedentary or sitting time between population subgroups, minutes/day. [file 12966_2020_938_MOESM9_ESM.png]

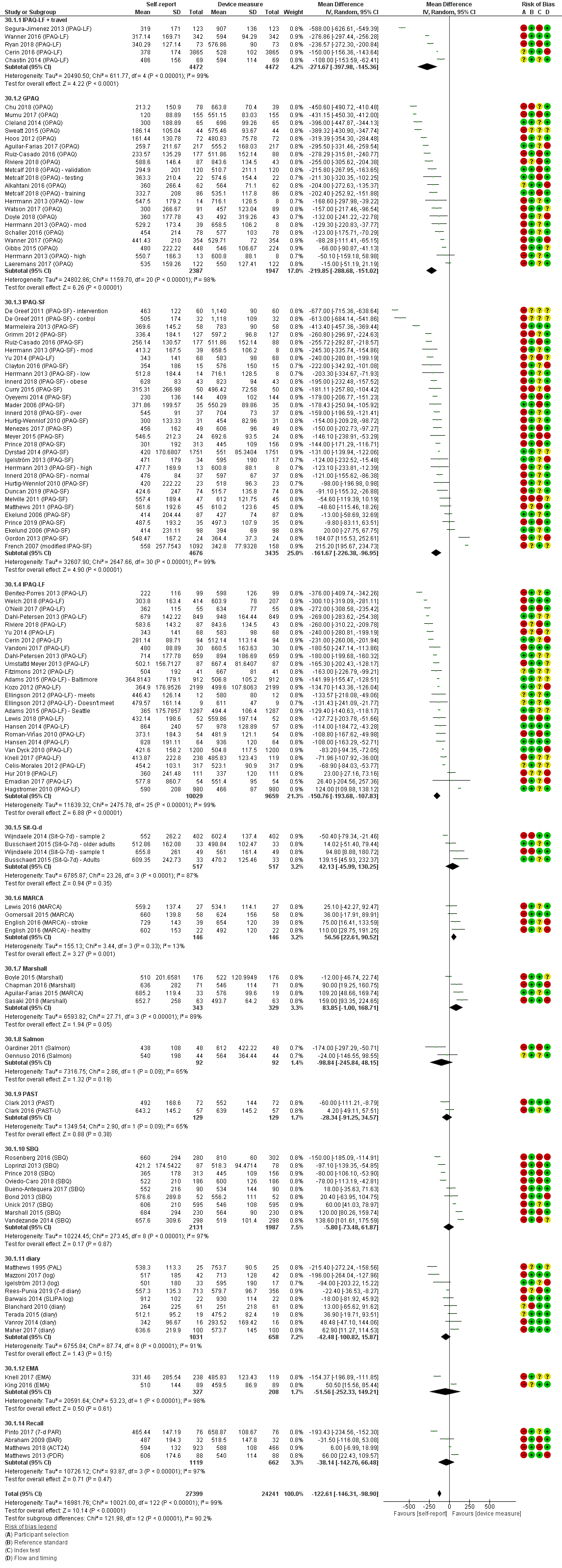

Supplement: Supplementary file 10 — Additional file 10: Supplemental figure 10. Forest plot comparing self-report and device measures of total sedentary or sitting time across questionnaires, minutes/day. [file 12966_2020_938_MOESM10_ESM.png]

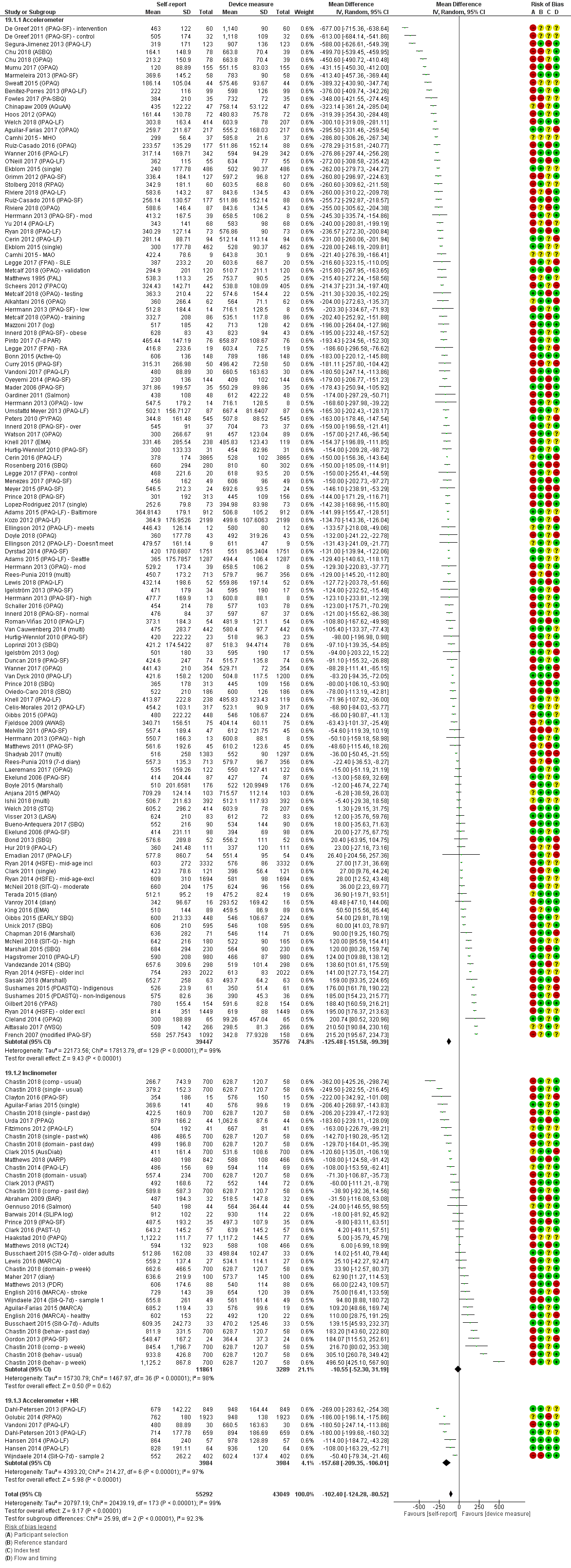

Supplement: Supplementary file 11 — Additional file 11: Supplemental figure 11. Forest plot comparing self-report and device measures of total sedentary or sitting time between devices, minutes/day. [file 12966_2020_938_MOESM11_ESM.png]

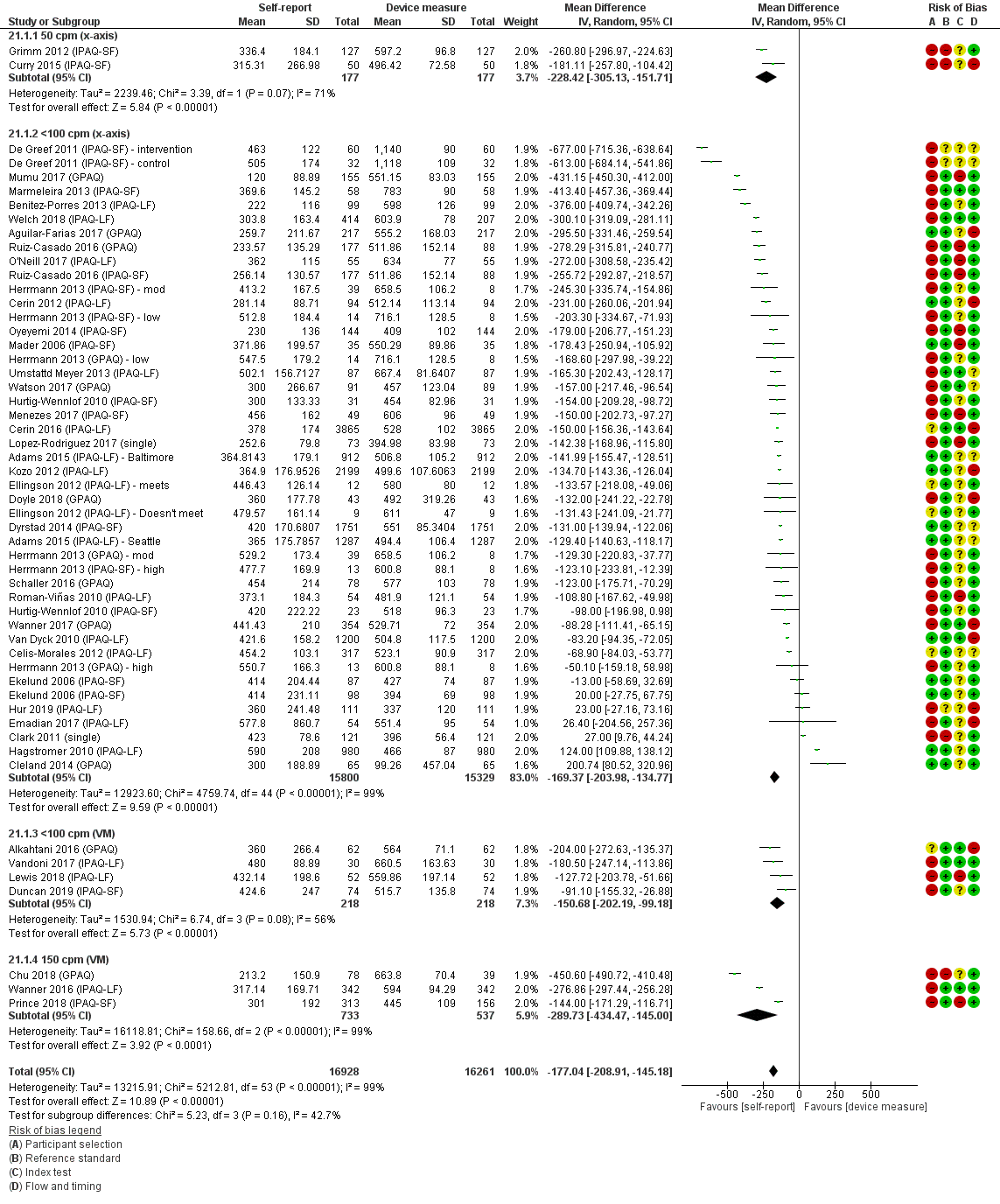

Supplement: Supplementary file 12 — Additional file 12: Supplemental figure 12. Forest plot comparing self-report and accelerometer measures of total sedentary or sitting time across cut-points, minutes/day. [file 12966_2020_938_MOESM12_ESM.png]

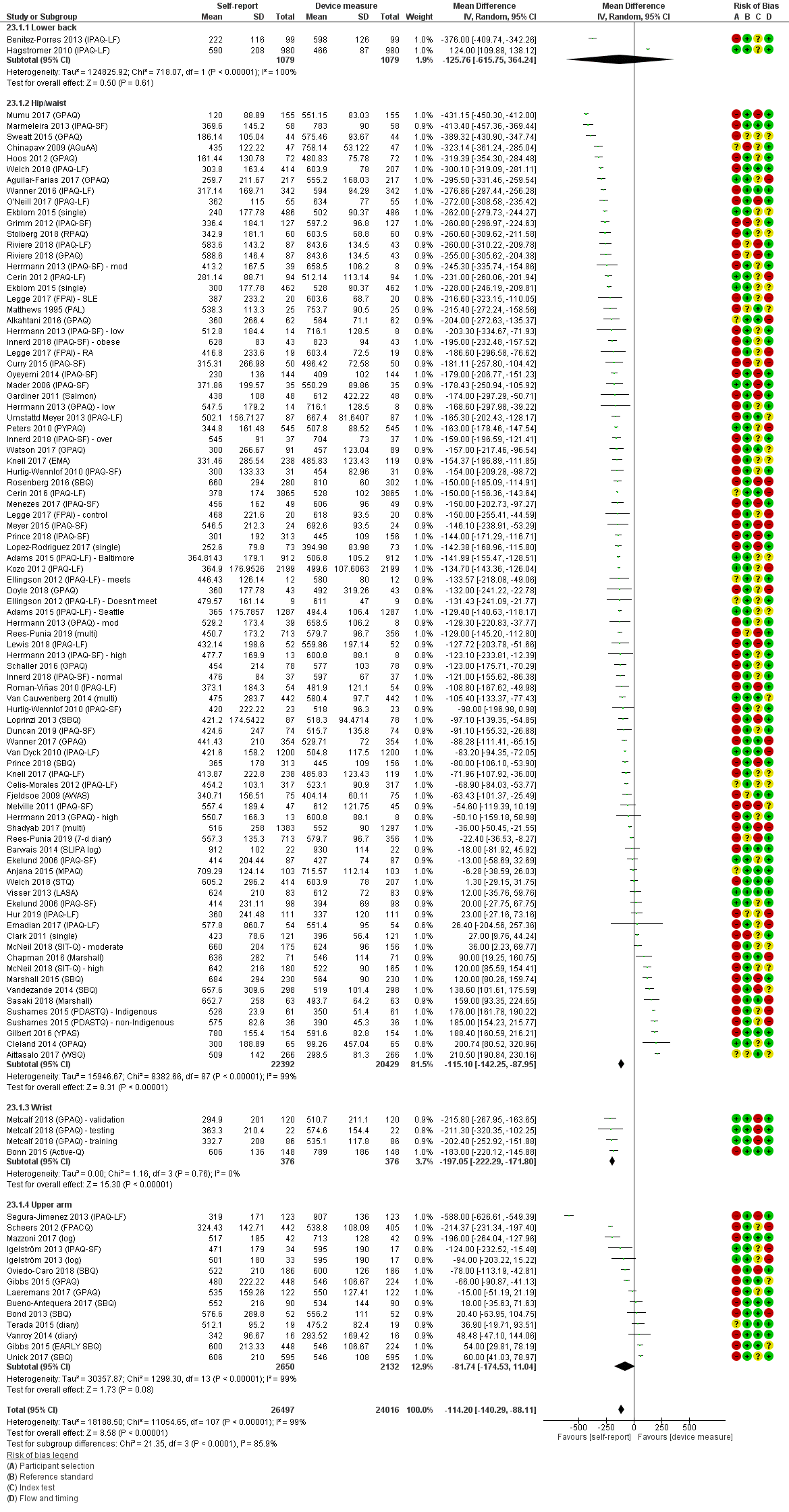

Supplement: Supplementary file 13 — Additional file 13: Supplemental figure 13. Forest plot comparing self-report and device measures of total sedentary or sitting time across wear locations, minutes/day. [file 12966_2020_938_MOESM13_ESM.png]
