# Supplementary material for: A comparison of self-reported and device measured sedentary behaviour in adults: a systematic review and meta-analysis
Source: Int J Behav Nutr Phys Act. 2020 Mar 4;17:31. doi: 10.1186/s12966-020-00938-3 (PMC7055033; doi:10.1186/s12966-020-00938-3)
Supplement: Supplementary file 14 — Additional file 14: Supplemental table 1. Ovid MEDLINE search strategy. [file 12966_2020_938_MOESM14_ESM.docx]

**Supplemental table 1. Ovid MEDLINE search strategy.**

1. Sedentary Lifestyle/

2. Sedentar*.ti,ab,kw.

3. (Physical* adj2 inactiv*).ti,ab,kw.

4. ((watch* or view* or time) adj3 (television? or TV)).ti,ab,kw.

5. ((watch* or view*) adj3 (video? or YouTube)).ti,ab,kw.

6. ((social media or blogging or Facebook or Youtube or Twitter or Snapchat or Instagram or Pinterest or Skype or Vine) adj3 ("use" or using or time)).ti,ab,kw.

7. ((reclining or driving or commut*) adj3 time).ti,ab,kw.

8. (screen? adj3 (time or view*)).ti,ab,kw.

9. sitting.ti,ab,kw.

10. (computer? adj3 (time or "use" or using)).ti,ab,kw.

11. (play* adj3 (video game* or computer game* or electronic game*)).ti,ab,kw.

12. Read*.ti,ab,kw.

13. Video Games/

14. Reading/

15. 1 or 2 or 3 or 4 or 5 or 6 or 7 or 8 or 9 or 10 or 11 or 12 or 13 or 14

16. Self Report/

17. "Surveys and Questionnaires"/

18. interview/

19. Mental Recall/

20. Self report*.ti,ab,kw.

21. Questionnaire?.ti,ab,kw.

22. (Diary or diaries).ti,ab,kw.

23. (Log? or logged or logging).ti,ab,kw

24. Survey*.ti,ab,kw.

25. Interview*.ti,ab,kw.

26. Recall*.ti,ab,kw.

27. Active-Q.ti,ab,kw.

28. AQuAA.ti,ab,kw.

29. Community Health Activities Model Program for Seniors.ti,ab,kw.

30. SIT-Q-7d.ti,ab,kw.

31. Measure of Older Adults* Sedentary Time.ti,ab,kw.

32. "Multimedia Activity Recall for Children and Adults".ti,ab,kw.

33. Past-day Adults* Sedentary Time-University.ti,ab,kw.

34. Past-day Adults* Sedentary Time.ti,ab,kw.

35. Previous Day Recall.ti,ab,kw.

36. Rapid Assessment Disuse Index.ti,ab,kw.

37. SIT-Q.ti,ab,kw.

38. 16 or 17 or 18 or 19 or 20 or 21 or 22 or 23 or 24 or 25 or 26 or 27 or 28 or 29 or 30 or 31 or 32 or 33 or 34 or 35 or 36 or 37

39. accelerometry/ or actigraphy/

40. Wearable Electronic Devices/

41. monitoring, physiologic/ or monitoring, ambulatory/

42. (objective* measure* or (objectively adj2 measur*)).ti,ab,kw.

43. ((Activit* or inactivit*) adj3 monitor*).ti,ab,kw.

44. (Acceleromet* or Inclinomet* or Actigraph* or Activpal? or Sensewear* or Actical? or Actitrainer? or pedometer?).ti,ab,kw.

45. ((Heart rate or physiolog*) adj2 monitor*).ti,ab,kw.

46. (wearable adj3 (technolog* or device*)).ti,ab,kw.

47. ((screen-based or Smart watch* or smartphone* or smart device* or cell phone* or cellular phone*or texting or text messag* or app or apps or iphone? or ipad? or ipod? or tablet? or laptop?) adj4 (monitor* or measur*)).ti,ab,kw.

48. 39 or 40 or 41 or 42 or 43 or 44 or 45 or 46 or 47

49. comparative study/

50. (comparison? or comparative or comparing or compare?).ti,ab,kw.

51. validity.ti,ab,kw.

52. 49 or 50 or 51

53. 15 and 38 and 48 and 52

54. (child/ or Adolescent/) not exp adult/

55. 53 not 54

56. exp animals/ not humans/

57. 55 not 56

58. (addresses or autobiography or bibliography or biography or comment or dataset or dictionary or directory or duplicate publication or editorial or government publications or guideline or interactive tutorial or lectures or legal cases or legislation or news or newspaper article or patient education handout or periodical index or practice guideline or technical report or video-audio media or webcasts).pt.

59. 57 not 58
