# Supplementary material for: A comparison of self-reported and device measured sedentary behaviour in adults: a systematic review and meta-analysis
Source: Int J Behav Nutr Phys Act. 2020 Mar 4;17:31. doi: 10.1186/s12966-020-00938-3 (PMC7055033; doi:10.1186/s12966-020-00938-3)
Supplement: Supplementary file 15 — Additional file 15: Supplemental table 2. Study characteristics. [file 12966_2020_938_MOESM15_ESM.pdf]

Supplemental table 2. Study characteristics

First Author, year	Age range or mean (SD)	N analyzed (%)			Country, Study name	Population	Self-report measure					Device measure					
		Total	Men	Women			Name	Multi-item or single	Measure type	Recall period*	Domain or type	Name	Domain or type	Wear time	Cut-point	Axis	Wear location
Abraham, 2009	Men: 29.1 (7.9), women: 30.2 (9.5)	32	16 (50%)	16 (50%)	Australia, NR	Apparently healthy individuals who run	Bouchard Activity Record (BAR)	Multi	Daily recall	24-hours	Sitting (category 1 and 2)	ActiGraph GT1M	ST	24 hours (wake ful time)	< 100 cpm	Verti cal	Right hip
												activPAL Prof.	Sitting/ lying		N/A	N/A	Right thigh
Adams, 2015	20-65, Seattle: 44.0 (11.0), Baltimore 46.6 (10.7)	2199, Seattle: 1287, Baltimore: 912	51.8%, Seattle: 54.8%, Baltimore: 47.7%	48.2%, Seattle: 45.2%, Baltimore: 52.3%	USA, NQLS	Apparently healthy	IPAQ-LF	Single (weekd ay + weeken d)	Quest.	Last 7 days	Sitting	ActiGraph 7164 or 71256	ST	7 days	≤ 100 cpm	Verti cal	Right hip
Aguilar-Farias, 2015	74.5 (7.6)	41: 37 activPAL; 40 SB question; 33 MARCA	14	27	Australia, The Time of Your Life study	Community-dwelling older adults	Sitting question (weekday, weekend day)	Single	Quest.	Usual weekday, weekend day, yesterday	Sitting	activPAL	ST	7 days	NA	N/A	Right thigh
							MARCA	Multi		Past 2 days	ST						
Aguilar-Farias, 2017	43.8 (15.75)	217	93 (43)	124 (57)	Chile, National Health Survey	General population	GPAQ	Single	Quest.	Usual sitting on normal day	Sitting	ActiGraph GT3X	ST	7 days	< 100 cpm	Verti cal	Right hip
Aittasalo, 2017	42.6 (10.9)	266	100%	64%	Finland, Moving to Business	Employees from small to medium workplaces	WSQ	Multi	Quest.	Typical working day, typical non-working day	Sitting (work, home, travel)	Hookie AM13	ST	7 days	NR	Tri-axial	Right hip
Alkahtani, 2016	20.0 (1.1)	62	62 (100)	0	Saudia Arabia, NR	Male university students	GPAQ	Single	Quest.	Usual sitting on typical day	Sitting	ActiGraph GT3X	ST	7 days	< 100 cpm (VM)	VM	Right hip
Anjana, 2015	Urban: 44.4 (14.2), rural: 41.7 (13.4)	103	NR	NR	India, NR	Urban and rural populations	MPAQ	Multi	Quest.	Typical week	TV, sitting	ActiGraph GT3X	ST	7 days	< 100 cpm	VM	Dominan t hip (mostly right)

First Author, year	Age range or mean (SD)	N analyzed (%)			Country, Study name	Population	Self-report measure					Device measure					
		Total	Men	Women			Name	Multi-item or single	Measure type	Recall period*	Domain or type	Name	Domain or type	Wear time	Cut-point	Axis	Wear location
Barone Gibbs, 2017	68 (7)	38	11	27	USA, NR	Older adults	CHAMPS	Multi	Quest.	Unclear	TV, computer (work or recreation), riding in car	SenseWear Pro	ST	7 days	N/A	N/A	Arm
Barwais, 2014	27 (4.5)	22	14	8	Australia, NR	Sedentary adults	7-Day SLIPA log	N/A	Log	7 day log	Sum of SBs	ActiGraph GT3X	ST, sitting	7 days	< 100 cpm & inclinometer	NR	Right hip
Benitez-Porres, 2013	18-75	99	5	94	Spain, Al-Andalus Study	Fibromyalgia patients	IPAQ-LF (sitting questions)	Single (week day & weekend)	Quest.	Last 7 days	Sitting	ActiGraph GT1M	ST	9 days	< 100 cpm	Vertical	Lower back
Biddle, 2012	32.5 (5.6)	IPAQ: 131, Marshall: 128	36%	64%	NR, Project STAND	Young adults at risk for diabetes	IPAQ-SF (sitting only)	Single	Quest.	Last 7 days	Sitting	ActiGraph GT3X	ST	10 days	< 100 cpm	NR	Right hip
							Marshall Quest.	Multi		Not defined	ST						
Biswas, 2018	66.9 (10.5)	30 (with both)	13 (43%)	17 (57%)	Canada, NR	Cardiac rehab patients	SBQ	Multi	Quest.	Typical weekday and weekend day	SBs	ActiGraph GT3X	ST	7 days	< 150 cpm	NR	Hip
Blanchard, 2010	80.4 (5.5), 67-92	61	41%	59%	Canada, NR	Older adults	Diary	N/A	Diary	1 week	Driving	CarChip E/X	Driving time	1 week	N/A	N/A	N/A
Blikman, 2014	NR	19	NR	NR	Netherlands, NR	Patients after total hip Arthroplasty	IPAQ-SF & LF (NR)	Single	Quest.	Last 7 days	Sitting	NR	ST	NR	NR	NR	NR
Bond, 2013	46.2 (9.1), 21-65	52	14%	86%	USA, NR	Bariatric surgery patients	SBQ	Multi	Quest.	Typical weekday and weekend day	SBs	SenseWear Pro2	ST	7 days	N/A	Tri-axial	Arm
Bonn, 2015	65.4 (8.7)33-86	148	148	0	Sweden, VALidation against	Men who underwent Prostate Specific	Active-Q	Multi	Quest.	Past month	SBs	GENEA	ST	7 days	1.5 MET	VM	Left wrist

First Author, year	Age range or mean (SD)	N analyzed (%)			Country, Study name	Population	Self-report measure					Device measure					
		Total	Men	Women			Name	Multi-item or single	Measure type	Recall period*	Domain or type	Name	Domain or type	Wear time	Cut-point	Axis	Wear location
					Accelerome TER	Antigen testing											
Boyle, 2015	27-82	176	54%	46%	Australia, Canada, VALidation against Accelerome TER	Colon cancer survivors	Marshall Domain-Specific Sitting Quest.	Multi	Quest.	Usual weekday and weekend	SBs	ActiGraph GT3X	ST	7 days	< 100 cpm	NR	NR
Bruening, 2016	18.72 (0.50)	41	11 (27%)	30 (73%)	USA, SPARC study	College students	devilSPARC mEMA	N/A	mobile-based EMA	4 days	SBs	ActiGraph GT3X	ST	4 days	< 100 cpm	NR	NR
Bueno-Antequera, 2017	41.6 (9.2)	90	80%	18 (20%)	Spain, The PsychiActive project	Outpatients with severe mental illness	SBQ	Multi	Quest.	Usual weekday and weekend	SBs	SenseWear	ST	9 days	N/A	Bi-axial	Left arm
Busschaert, 2015	Adults: 47.73 (10.51), older adults: 72.16 (4.35)	33 adults, 33 older adults	Adults: 36.4%, older adults: 60.6%	Adults: 63.6%, older adults: 39.4%	Belgium, NR	General population	Last-7-day SB Quest. (SIT-Q-7d)	Multi	Quest.	Past 7 days	Sitting/lying down	activPAL	Sitting	7 days	N/A	N/A	Thigh
Camhi, 2015	26.7(4.7); 19-35	MHO = 37, MAO = 9	0	100%	NR, NR	Metabolically healthy obese (MHO) and metabolically abnormal obese (MAO), in young black and white women	NR	NR	Quest.	NR	Sitting	NR	ST	7 days	< 100 cpm	NR	NR
Celis-Morales, 2012	37.5 (12.8)	317	140	177	Scotland, Chile, NR	Chilean population (general pop)	IPAQ-LF (Spanish version – sitting questions)	Single (weekday + weekend)	Quest.	Previous 7 days	Sitting	Actitrainer	ST	7 days	< 100 cpm	NR	Left hip

First Author, year	Age range or mean (SD)	N analyzed (%)			Country, Study name	Population	Self-report measure					Device measure					
		Total	Men	Women			Name	Multi-item or single	Measure type	Recall period*	Domain or type	Name	Domain or type	Wear time	Cut-point	Axis	Wear location
Cerin, 2012	>65	94	42%	58%	Hong Kong, NR	Urban older adults	IPAQ-LF (Chinese – sitting questions)	Single (weekday + weekend)	Quest.	Last 7 days	Sitting	ActiGraph GT1M	ST	7 days	< 100 cpm	Vertical	Right hip
Cerin, 2016	43.1 (12.4)	3865	48.10%	51.9%	Belgium, Czech Republic, Denmark, Spain, UK, US, International Physical Activity and the Environment Network Adult study	General population	IPAQ-LF (sitting + motorized)	Multi (weekday + weekend)	Quest.	Last 7 days	Sitting including motorized transport	ActiGraph GT1M and GT3X	ST	7 days	< 101 cpm	Vertical	Right hip
Chapman, 2016	40.3 (11.4)	71	NR	NR	Australia, NR	Non-institutionalised adults with mental illness	Marshall Domain-Specific Sitting Quest.	Multi	Quest.	Previous week	Sitting: travel; at work; TV; computer use; leisure time (not including TV)	ActiGraph GT3X	ST	7 days	≤ 100 cpm	Vertical	Right hip
Chasan-Taber, 2004	16-40	54	0	54	USA, NR	Pregnant women	Pregnancy physical activity Quest. (PPAQ)	Multi	Quest.	Current trimester	SBs: household /care giving, occup., sports/ exercise, travel, inactivity	ActiGraph MTI	ST	7 days	NR	Vertical	Right hip
Chastin, 2018	Cohort ages were 64 (T07 1950s cohort, n = 310), 79 (LBC1936 , n = 271),	700	339 (48%)	361 (52%)	Scotland, Seniors USP project	Seniors	Two direct measures of ST	Single	Quest.	Past day, usual day or past week	Sitting, TV, multi-SBs	activPAL	Sitting/ lying	7 days	N/A	N/A	Thigh (dominant leg)
							Direct question about total time sitting	Single	Quest.		Sitting						

First Author, year	Age range or mean (SD)	N analyzed (%)			Country, Study name	Population	Self-report measure					Device measure					
		Total	Men	Women			Name	Multi-item or single	Measure type	Recall period*	Domain or type	Name	Domain or type	Wear time	Cut-point	Axis	Wear location
	and 83 (T07 1930s cohort, n = 119)						Visual analogue scale of the proportion of day sitting	Single	Quest.		Sitting						
							Single item proxy measure (TV time)	Single	Quest.		TV						
							Composite pattern: number of bouts of sitting and their average duration (multiplied to get total duration)	Multi	Quest.		Multiple SBs						
							Composite sum: TV, work, computer/ screen for leisure, reading, listening or playing music, engaging in seated hobbies, talking, eating, self-care, performing activities of daily living, napping, sitting in transport and sitting during leisure activities	Multi	Quest.								

First Author, year	Age range or mean (SD)	N analyzed (%)			Country, Study name	Population	Self-report measure					Device measure					
		Total	Men	Women			Name	Multi-item or single	Measure type	Recall period*	Domain or type	Name	Domain or type	Wear time	Cut-point	Axis	Wear location
							outside the home										
							Composite domain sum: work, home, transport and leisure	Multi	Quest.		Domain-specific SB						
Chastin, 2014	41.1 (9.0)	69	23	46	Scotland, NR	General population	IPAQ-LF (sitting and sitting + motorized)	Single (weekday + weekend day), multi	Quest.	Last 7 days	Sitting with and without motorized transport	activPAL	Sitting/ lying	7 days	N/A	N/A	Thigh
Chau, 2012	>18	76	60%	40%	Australia, NR	Workers	OSPAQ	Multi	Quest.	Past 7 days	Occup. sitting	ActiGraph GT1M + log	ST	7 days	< 100 cpm	Vertical	Right hip
Chau, 2011	>18	95; at work = 81, total all domains workday = 82, total all domains non-workday = 76, average total = 82.<br><br>Women: at work = 50, total all domains workday = 51, total all domains non workday = 45, average total = 51. Men: all analyses = 31	35 (36.8%)	60 (63.2%)	Australia, NR	Workers	WSQ	Multi	Quest.	Past 7 days	Total all-domain sitting, occup. sitting, TV, computer at home, leisure activities, travel on workdays and non-workdays	ActiGraph GT1M	ST	7 days	< 100 cpm	Vertical	Right hip
		75 for all analyses. IPAQ = 48 for all					IPAQ-SF	Single		Last 7 days	Sitting						

First Author, year	Age range or mean (SD)	N analyzed (%)			Country, Study name	Population	Self-report measure					Device measure					
		Total	Men	Women			Name	Multi-item or single	Measure type	Recall period*	Domain or type	Name	Domain or type	Wear time	Cut-point	Axis	Wear location
		analyses. Men: all analyses = 27															
Chau, 2014	38 (11)	42; activPAL = 32; WSQ at work on workday = 31	6 (14%)	36 (86%)	Australia, Stand@Work	Workers	WSQ	Multi	Quest.	Past 7 days	Occup. sitting, TV, computer at home, leisure activities, travel on workdays and non-workdays	activPAL3	Sitting/ lying at work	Work week during work time	N/A	N/A	Thigh
		activPAL = 32; OSPAQ = 33					OSPAQ				Occup. sitting						
Chinapaw, 2009	25-38, reliability = 28.9 (3.5), validity = 30.1 (3.6)	Reliability = 58; validity = 47	Reliability = 20, validity 17	Reliability = 28, validity = 41	Netherlands, NR	Workers	AQuAA	Multi	Quest.	Past 7 days	SBs	ActiGraph 7164	ST	2 weeks	< 699 cpm	Vertical	Right hip
Chu, 2018	20-65	Reliability = 84, validity = 78	31%	69%	Singapore, NR	University desk workers	ASBQ	Multi,	Quest.	Typical week	Sum of SBs: occup. + travel + leisure + other	ActiGraph GT3X BT	ST	7 days	< 150 cpm	VM	NR
							GPAQ	Single	Quest.	Usual sitting on a typical day	Sitting (single question)						
Clark, 2015	36-89, mean=59	700	45%	55%	Australia, NR	General population	AusDiab3 Quest.	Multi	Quest.	Past 7 days	Sum of weekday + weekend SBs: work, travel, TV, leisure computer use and	activPAL3	Sitting	7 days	N/A	N/A	Right thigh

First Author, year	Age range or mean (SD)	N analyzed (%)			Country, Study name	Population	Self-report measure					Device measure					
		Total	Men	Women			Name	Multi-item or single	Measure type	Recall period*	Domain or type	Name	Domain or type	Wear time	Cut-point	Axis	Wear location
											"other" purposes						
Clark, 2016	18-55	57	30 (53%)	27 (47%)	Australia, NR	University population (students and staff)	Past-day Adults' Sedentary Time-University (PAST-U)	Multi	Quest.	Past day	Sitting or lying down for work, study, travel, TV viewing, leisure-time computer use, reading, eating, socialising and other purposes	activPAL	Sitting	1 day	N/A	N/A	Right thigh
Clark, 2011	median = 34.9 (IQR: 28.5–46.0)	121	48 (36%)	73 (60%)	Australia, Stand Up Australia	Full time workers	NR	Single	Quest.	Past week	Occupational sitting, breaks in sitting	ActiGraph GT1M + log of work hours	ST at work	7 days	< 100 cpm	Vertical	Right hip
Clark, 2013	33-75, 56 (9)	Reliability = 86, validity with activPAL = 72 (baseline); validity with ActiGraph = 59	0	100%	Australia, Living Well after Breast Cancer	Breast cancer survivors	Past-day Adults' Sedentary Time (PAST)	Multi	Quest.	Past day	Single weekday time spent sitting or lying: at work, travelling, TV, computer (excluding work), reading (excluding work), hobbies, and any other not reported in previous items	activPAL3	Sitting/ lying	7 days	N/A	N/A	Right thigh
												ActiGraph GT3X	ST		< 100 cpm	Vertical	Right hip

First Author, year	Age range or mean (SD)	N analyzed (%)			Country, Study name	Population	Self-report measure					Device measure					
		Total	Men	Women			Name	Multi-item or single	Measure type	Recall period*	Domain or type	Name	Domain or type	Wear time	Cut-point	Axis	Wear location
Clayton, 2016	77.4 (7.5)	15	15	0	USA, NR	Men with prostate cancer and history of Androgen-Deprivation Therapy use	IPAQ-SF	Single	Quest.	Usual day past 7 days	Sitting (single question)	activPAL3	Sitting	7 days	N/A	N/A	Thigh
Cleland, 2014	44 (14)	65	31 (48%)	34 (52%)	Ireland, NR	Middle-aged men and women	GPAQ	Single	Quest.	Usual day	Sitting (single question)	ActiGraph GT3X	ST	7 days	≤ 100 cpm	NR	Hip
Cleland, 2018	71.8 (6.6)	228	129	97	Ireland, Healthy Urban Living and Ageing in Place	Older adults	IPAQ-LF	Single (weekday + weekend day)	Quest.	Usual day previous 7 days	Sitting	ActiGraph GT3X	ST	7 days	≤ 99 cpm	NR	Right hip
Clemes, 2012	41.5 (12.8)	44	30%	70%	UK, NR	University staff	NR	Single	Daily sitting log	Past day sitting	Sitting	ActiGraph GT1M	ST	7 days	< 100 cpm	Vertical	NR
								Multi	Quest.	Usual weekday and weekend day	Travel, work, TV, using a computer at home, and leisure time, not including TV (e.g. visiting friends, movies, dining out etc.)						
Conroy, 2013	21.3 (1.1)	128	53	75	USA, NR	College students	IPAQ-SF (modified)	Single	Quest.	Daily recall	Sitting (single question)	ActiGraph GT3X	ST	7 days	< 100 cpm	NR	Right hip
Craig, 2003	Aust = 35.9 (11.7), BRA = 42.9 (14.2), UK1 =	Aus = 62, Brazil = 28, UK1 = 149, UK2 = 101, FIN = 88, GU = 41, 40, 34, 40, CSA	Aus = 40 (65%), Brazil = 12 (43%), UK1 = 68 (46%), UK2 = 38	Aus = 22 (35%), Brazil = 16 (57%), UK1 = 81 (54%), UK2 = 63	Australia, Brazil, Canada, Finland, Guatemala, Netherlands, Japan,	General population	IPAQ-SF, IPAQ-LF (sitting question only)	Single	Quest.	Tested last 7 days and usual week (meta-analysis	Sitting (single question)	CSA 7164	ST	7 days	< 100 cpm	Vertical	NR

First Author, year	Age range or mean (SD)	N analyzed (%)			Country, Study name	Population	Self-report measure					Device measure					
		Total	Men	Women			Name	Multi-item or single	Measure type	Recall period*	Domain or type	Name	Domain or type	Wear time	Cut-point	Axis	Wear location
	35.2 (10.6), UK2 = 41.1 (8.4), CAN = 49.2 (14.8), 46.5 (16.5), 39.1 (17.5), FIN = 55.6 (8.6), GU = 25.9 (5.0), NET = 32.7 (10.9), JAP = 33.8 (10.2), PORT = 351 (11.5), USA1 = 48.9 (6.1), SA = 31.8 (12.0), 35.6 (12.1), 32.3 (8.5), 31.6 (7.5), USA2 = 37.4 (11.9), 36.1 (12.8), SW = 40.7 (10.8), 47.1 (13.6)	61, Netherlands = 74, Japan = 144, Portugal = 196, USA1 = 28, SA = 144, 108, 107, 92, USA2 = 30, 30, Sweden = 50, 200	(38%), FIN = 43 (49%), GU = 23 (56%), 20 (50%), 16 (47%), 19 (48%), 45 (73%), Netherlands = 34 (40%), Japan = 74 (51%), Portugal = 96 (49%), USA1 = 7 (25%), SA = 76 (53%), 48 (44%), 54 (51%), 46 (50%), USA2 = 15 (50%), 14 (47%), Sweden = 22 (44%), 77 (39%)	(62%), FIN = 45 (51%), GU = 18 (44%), 20 (50%), 18 (53%), 21 (52%), 16 (27%), Netherlands = 40 (60%), Japan = 70 (49%), Portugal = 100 (51%), USA1 = 21 (75%), SA = 68 (47%), 60 (56%), 53 (49%), 46 (50%), USA2 = 15 (50%), 16 (53%), Sweden = 28 (56%), 123 (61%)	Portugal, South Africa, Sweden, United States, United Kingdom, NR					used weekday on past 7 days)							
Curry, 2015	40.1 (10.5)	50	0	50 (100%)	Wales, NR	South Asian women	IPAQ-SF	Single	Quest.	Previous 7 days	Sitting	ActiGraph GT1M and GT3X	ST	7 days	≤ 50 cpm	Vertical	Waist
Dahl-Petersen, 2013	men = 44.8 (14.2),	1508	659	849	Greenland, NR	Inuit	Modified IPAQ-LF (Greenlandic	Single	Quest.	Previous 7 days	Sitting	Actiheart	ST	2-5 days	< 1.5 MET	NR	Chest

First Author, year	Age range or mean (SD)	N analyzed (%)			Country, Study name	Population	Self-report measure					Device measure					
		Total	Men	Women			Name	Multi-item or single	Measure type	Recall period*	Domain or type	Name	Domain or type	Wear time	Cut-point	Axis	Wear location
	women = 43.1 (13.8)						, sitting questions)										
De Cocker, 2016	40.3 (9.1)	122; WSQ: tailored = 36, generic = 64, control = 28; ActivPAL: tailored = 35, generic = 35, control = 23	NR	NR	Belgium, NR	Flemish employees	WSQ	Multi	Quest.	Last 7 days	Sitting on workday and non-workday while: in travel, at work, TV, computer at home (non-work related), and; doing other leisure activities	activPAL	Sitting	5 days (work days)	N/A	N/A	Thigh
De Greef, 2011	62 (9)	92; Intervention = 60, control = 32	69%	31%	Belgium, NR	Patients with Type 2 diabetes	IPAQ (version NR)	Single	Quest.	Last 7 days	Sitting	ActiGraph 7164	ST	5 days	< 100 cpm	Vertical	NR
Doyle, 2018	Reliability: 21.27 (2.32), validity: 19.30 (1.87)	Validity = 43, reliability = 86	Reliability: 41%, validity: 39.5%	Reliability: 59%, validity: 60.5%	United Arab Emirates, NR	Emirati university students	GPAQ (Arabic version)	Single	Quest.	Typical week	Sitting	ActiGraph GT3X	ST	7 days	< 100 cpm	NR	Right hip
Duncan, 2019	Validity: 42.3 (11.9) , reliability: 41.9 (11.8)	74	Validity: 47, reliability: 48	Validity: 27, reliability: 26	Canada, NR	Individuals with schizophrenia	IPAQ-SF	Single (week day)	Quest.	Last 7 days	Sitting	ActiGraph GT3X	ST	7 days	≤ 99 cpm	VM	Right hip
Dyrstad, 2014	48.2 (14.1)	1751	878 (50.1%)	873 (49.1%)	Norway, NR	General population	IPAQ-SF	Single	Quest.	Last 7 days	Sitting	ActiGraph GT1M	ST	7 days	< 100 cpm	Vertical	NR
Ekblom, 2015	50-65	948	462	486	Sweden, SCAPIS pilot study	General population	NR	Single	Quest.	Previous 7 days	Sitting	ActiGraph GT3X	ST	4 days	< 200 cpm	VM	Right hip

First Author, year	Age range or mean (SD)	N analyzed (%)			Country, Study name	Population	Self-report measure					Device measure					
		Total	Men	Women			Name	Multi-item or single	Measure type	Recall period*	Domain or type	Name	Domain or type	Wear time	Cut-point	Axis	Wear location
Ekelund, 2006	20-69; men 42 (13); women 41.6 (13)	185	87	98	Sweden, NR	Workers	IPAQ-SF	Single	Quest.	Last 7 days	Sitting	ActiGraph 7164	ST	7 days	< 100 cpm	Vertical	Waist
Ellingson, 2012	30.0 (5.8)	21; meets recommendations = 12; inactive = 9	0	21	USA, NR	Healthy women	IPAQ-LF (sitting questions only)	Single (weekday + weekend day)	Quest.	Last 7 days	Sitting	ActiGraph GT1M	ST	7 days	< 100 cpm	Vertical	Hip
Elramli, 2017	56 (15)	76; 39 intervention, 37 control	13	63	Scotland, NR	People within first 5 years of being diagnosed with rheumatoid arthritis	IPAQ-LF (sitting questions only)	Single (weekday + weekend day)	Quest.	Previous 7 days	Sitting	activPAL	Sitting/ lying	7 days	N/A	N/A	Right thigh
Emadian, 2017	45.0 (9.79)	54	54 (100%)	0	England, NR	Overweight & obese South Asian men	IPAQ-LF (sitting questions only)	Single (weekday + weekend day)	Quest.	Last 7 days	Sitting	ActiGraph GT3X	ST	7 days	< 100 cpm	NR	Right hip
English, 2016	Stroke: 67.2 (11.1); healthy control: 70.4 (7.8)	Stroke = 39, healthy = 22	Stroke: 26, control: 14	Stroke: 14, control: 8	Australia, NR	Individuals who had experienced a stroke	MARCA	Multi	Quest.	Past day	SBs (TV, video games, reading, computer)	activPAL3	Sitting	7 days	N/A	N/A	Thigh (non-paretic thigh or right)
Fitzsimons, 2012	37 (11)	41	23	18	Scotland, NR	Scottish adults	IPAQ-LF (weekday only)	Single	Quest.	Last 7 days	Sitting (weekday)	activPAL3	Sitting/ lying	3 days	N/A	N/A	NR
Fitzsimons, 2013	68 (6)	24; 22 activPAL, 23 SBQ	14	10	Scotland, NR	Older Scottish adults	SBQ	Multi	Quest.	Usual weekday and weekend	Total ST (combined items)	activPAL3	Sitting/ lying	7 days	N/A	N/A	NR
Fjeldsoe, 2009	Validity = 32 (5), reliability = 33 (5)	Validity = 75, reliability = 40	0	Validity = 75, reliability = 40	Australia, NR	Women with young children	AWAS	Multi	Quest.	Typical week in past month	Sitting (across multiple domains)	ActiGraph MT1	ST	7 days	≤ 100 cpm	Vertical	Waist

First Author, year	Age range or mean (SD)	N analyzed (%)			Country, Study name	Population	Self-report measure					Device measure					
		Total	Men	Women			Name	Multi-item or single	Measure type	Recall period*	Domain or type	Name	Domain or type	Wear time	Cut-point	Axis	Wear location
Fowles, 2017	Women: 55 (10); men: 63 (9)	35	6	26	Canada, NR	University population	Canadian Society for Exercise Physiology Physical Activity and Sedentary Behavior Quest.	Multi	Quest.	NR	Sum: occup. + leisure + total sitting	ActiGraph GT3X	ST	7 days	Wear time - MVPA	NR	NR
French, 2007	IPAQ: 47.6 (10.2)	IPAQ = 1092, ActiGraph = 158	IPAQ: 78.4%	IPAQ: 21.6%	USA, Route H Study	Transportation workers	Modified IPAQ-SF	Single	Quest.	Daily recall previous 7 days	Sitting	ActiGraph	ST	4 days	≤ 250 cpm	VM	NR
Gao, 2017	33.1 (10.7)	70	29 (41.4%)	41 (58.6%)	Finland, China, NR	Finnish and Chinese office workers	Long-term and short-term ST	Single	Quest.	Long-term: average workday in past 3 months; short-term: past day average ST	Occup. sitting	X6-1a	ST	5 work days	N/A	Used inclinometer	Thigh
Gardiner, 2011	72.8 (8.1)	48	13 (27.1%)	35 (72.9%)	Australia, Stand Up for Your Health	Older adults	Modified Salmon Quest.	Multi	Interview Quest.	Past week	Total ST: TV, computer use, reading, socialising, travel and hobbies, and a summary measure	ActiGraph GT1M	ST	6 days	< 100 cpm	Vertical	Right hip
Gennuso, 2015	75.1 (6.5)	58	21%	79%	USA, NR	Older adults	YPAS	Multi	Quest.	Typical week over past month	SBs	ActiGraph GT1M	ST	10 days	< 100 cpm	Vertical	Right hip

First Author, year	Age range or mean (SD)	N analyzed (%)			Country, Study name	Population	Self-report measure					Device measure					
		Total	Men	Women			Name	Multi-item or single	Measure type	Recall period*	Domain or type	Name	Domain or type	Wear time	Cut-point	Axis	Wear location
							CHAMPS	Multi	Quest.	Frequency / duration over past 4 weeks	SBs						
Gennuso, 2016	70 (8)	44	16	28 (64%)	USA, NR	Older adults	Modified Salmon Quest. (Gardiner)	Multi	Quest.	Past week	Sum: TV, computer, reading, socializing, driving or in public transportation, hobbies, and other activities	activPAL	Sitting/ lying	1 week	N/A	N/A	Right hip
Gibbs, 2015	median (IQR): 30.9 (27.8-33.7)	448	133 (29.7%)	315 (70.3%)	USA, IDEA Study	Overweight & obese young adults	EARLY Trials Sedentary Behavior Quest.	Multi	Quest.	Typical weekday and weekend	Sum: TV, computer or video games, office work, reading or other recreation, using a phone, and travel-work, using a computer, paperwork	SenseWear armband	ST	7 days	N/A	N/A	Arm
							GPAQ	Single		Typical day	Sitting/reclining						
Gilbert, 2016	55 .11 (13.91)	154 (valid accel data)	NR	Full sample: 142 (82.56%)	USA, Increasing Motivation for Physical Activity in Arthritis Clinical Trial (IM PAACT)	Patients with Rheumatoid Arthritis	modified YPAS	Multi	Quest.	Typical week in past month	SBs	ActiGraph GT1M	ST	7 days	< 100 cpm	Vertical	Right hip

First Author, year	Age range or mean (SD)	N analyzed (%)			Country, Study name	Population	Self-report measure					Device measure					
		Total	Men	Women			Name	Multi-item or single	Measure type	Recall period*	Domain or type	Name	Domain or type	Wear time	Cut-point	Axis	Wear location
Golubic, 2014	Women: 54.0 (9.3), men: 55.0 (9.9)	1923	580	1343	Denmark, France, Germany, Greece, Italy, Netherlands, Norway, Spain, Sweden, and UK, NR	Middle-aged men and women	Recent Physical Activity Quest. (RPAQ)	Multi	Quest.	Past 4 weeks	SBs: leisure time, occupation, commuting, and domestic life	Actiheart	ST	Minimum 4 days	≤ 1.5 METs	N/A	chest
Gomersall, 2015	28 (7.4)	58	30 (52%)	28 (48%)	Australia, NR	Adults from an Australian university	MARCA	Multi	Quest.	Previous day	SBs	activPAL	Sitting/ lying	24 hours (wake time)	N/A	N/A	Right thigh
Gordon, 2013	45.5 (12.7)	24	8	16	USA, NR	Adults with sedentary occupations	IPAQ-SF	Single	Quest.	Past 7 days	Total weekday sitting	ActiGraph GT3X	ST	1 week	< 100 cpm	NR	Right hip
							SBQ	Multi	Quest.	Usual weekday & weekend day	SBs	activPAL	Sitting/ lying at work		N/A	N/A	Thigh
Grimm, 2012	63.9 (7.7)	127	31	96	USA, NR	Older adults	IPAQ-SF	Single	Quest.	Previous week	Sitting	ActiGraph 7164	ST	7 days	≤ 50 cpm	Vertical	Right hip
Gupta, 2017	44.9 (9.8)	183	110 (60%)	73 (40%)	Denmark, New method for Objective Measurements of physical Activity in Daily living (NOMAD)	Blue collar workers	None	Single	Quest.	Past 24 hours	Work day sitting	ActiGraph GT3X+	Work day sitting	2 work days	Inclinometer function	N/A	Right thigh
Haakstad, 2010	32.3 (3.6)	77	0	77	Norway, NR	Pregnant women	PAPQ	Multi	Quest.	NR	Daily sitting/ lying	ActiReg	Sitting/ lying	7 days	Used inclinometer function	NR	Right thigh and chest
Hagstromer, 2010	18-65	980	443 (44%)	537 (56%)	Sweden, NR	General population	IPAQ-LF (sitting	Single (weekday +	Quest.	Last 7 days	Sitting	ActiGraph 7164	ST	7 days	< 100 cpm	Vertical	Lower back

First Author, year	Age range or mean (SD)	N analyzed (%)			Country, Study name	Population	Self-report measure					Device measure					
		Total	Men	Women			Name	Multi-item or single	Measure type	Recall period*	Domain or type	Name	Domain or type	Wear time	Cut-point	Axis	Wear location
							questions only)	weekend day)									
Hansen, 2014	49.0 (13.2)	121	57	64	Denmark, Danish Health Examination Survey	General population	IPAQ-LF (sitting questions only)	Single (weekday + weekend day)	Quest.	Past 7 days	Sitting	ActiHeart	ST	7 days	< 1.5 MET	N/A	Chest
Headley, 2018	48.48 (11.39)	127	86 (68%)	41 (32%)	USA, NR	College employees	OSPAQ	Multi	Quest.	Past 7 days	Occup. sitting	activPAL3	Sitting (occupational and total day)	7 days	N/A	N/A	Thigh
Hekler, 2012	75.3 (6.8)	870	44%	56%	USA, Seniors Neighborhood Quality Life Study	Older adults (65+)	CHAMPS	Multi	Quest.	Past 4 weeks	ST: TV, reading, socialize, passive transit, attend events	ActiGraph 7164 & 71256	ST	7 days	≤ 100 cpm	Vertical	Waist
Herrmann, 2013	Study 1: 43.1 ± 11.4, study 2: 40.2 ± 12.6	Study 1/ validity: 53 (wore accel), reliability: 16; GPAQ Category: Low (n = 14) ; GPAQ Category: Moderate (n = 39); GPAQ Category: High (n = 16)	Validity: 12, reliability: 8	Validity: 57, reliability: 8	USA, NR	General population	GPAQ	Single	Quest.	Usual day in a typical week	Sitting	ActiGraph GT1M	ST	7 days	< 100 cpm	Vertical	Waist
							IPAQ-SF			Weekdays last 7 days							
Hoos, 2012	43.01 (9.05)	72	0	72	USA, NR	Adult Latinas	GPAQ	Single	Quest.	Usual day	Sitting	ActiGraph GT1M	ST	7 days	NR	Vertical	Right hip
Hur, 2019	70 (9)	111	69 (62%)	42 (38%)	Canada, NR	Patients with fibrotic interstitial lung disease	IPAQ-LF (sitting questions only)	Single (weekday + weekend day)	Quest.	Last 7 days	Sitting	ActiGraph GT3X	ST	7 days	< 100 cpm	NR	Waist (non-dominant side)

First Author, year	Age range or mean (SD)	N analyzed (%)			Country, Study name	Population	Self-report measure					Device measure					
		Total	Men	Women			Name	Multi-item or single	Measure type	Recall period*	Domain or type	Name	Domai n or type	Wear time	Cut-point	Axis	Wear location
Hurtig-Wennlof, 2010	>65	54	23	31	Sweden, NR	Elderly (65+)	Modified IPAQ-SF	Single	Quest.	Last 7 days	Sitting	ActiGraph GT1M	ST	7 days	< 100 cpm	Verti cal	Waist
Hutchison, 2018	FM = 63.6 (7.40), CO = 72.5 (8.17)	35 (FM = 20, CO = 15)	20% FM =1, C = 6	80%; FM = 19, C = 9	USA, NR	Older adults (with and without fibromyalgia)	SBQ	Multi	Quest.	Typical weekday and weekend day	Total SB	ActiGraph WGT3X	ST	7 days	< 100 cpm	Verti cal	Waist
Igelström, 2013	median = 60 (IQR: 16)	34	NR	NR	Sweden, NR	People with obstructive sleep apnea and obesity	IPAQ-SF	Single	Quest.	Weekdays last 7 days	Sitting	SenseWear Pro 3	ST	5-7 days	N/A	N/A	Arm
		33					Logbook	N/A	Diary	Daily	Sitting						
Innerd, 2018	44 (9.2), 24-60	117; normal = 37, overweight = 37, obese = 43	57 (49%)	60 (51%)	UK, NR	University staff or students	IPAQ-SF	Single	Quest.	Past day (modified)	Sitting	ActiGraph GT3X+	ST	3 days	N/A	VM	Waist
Ishii, 2018	50.1 (7), reliability = 40.3 (11.4)	Validity = 392, reliability = 34	Validity = 156 (39.8%), reliability = 20 (58.8%)	Validity = 236 (60.2%), reliability = 14 (41.2%)	Japan, NR	Japanese adults	NR	Multi	Quest.	Past 7 days	Work and non-workdays ST in: car travel; public travel; at work; TV/videos /DVDs; computer, cell phone or tablet outside of work; in leisure time	Active Style Pro HJA-350IT	ST	7 days	≤ 1.5 MET S	Tri-axial	NR
Jancey, 2014	18+	Validity = 41, reliability = 99	Validity = 17, reliability = 36	Validity = 24, reliability = 63	Australia, NR	Office workers	OSPAQ	Multi	Quest.	Past 5 working days	Occup. sitting	ActiGraph GT3X+ (worn at work only)	ST	5 worki ng days	< 100 cpm	Verti cal	Waist or left thigh
Kim, 2018	33.3 (11.4)	27	15	12	USA, NR	University staff or students	Sedentary Behavior	Multi	Diary record	Past day	SBs	Autographer (camera + tri-axial	SBs	4 days (2 week	N/A	N/A	Around neck

First Author, year	Age range or mean (SD)	N analyzed (%)			Country, Study name	Population	Self-report measure					Device measure					
		Total	Men	Women			Name	Multi-item or single	Measure type	Recall period*	Domain or type	Name	Domain or type	Wear time	Cut-point	Axis	Wear location
							Record (SBR)					accelerometer))		day + 2 week end)			
Kim, 2017	46.2 (SE=0.4)	1458	610	848	USA, Physical Activity Measurement Survey	General population	24-Hour Physical Activity Recall	Multi	Recall diary	24 hour recall	SBs	SenseWear Armband Mini	ST	24 hours (excludes sleep)	N/A	N/A	Arm
King, 2016	60.0 (9.3)	89	22	67	USA, NR	Underactive adults	EMA	N/A	EMA	Past day	Sitting	Smart phone accelerometer	ST	24 hours (wakeful time)	≤ 56 cpm	NR	NR
Knell, 2017	43.3 (13.1)	238	115 (48.3%)	123 (51.7%)	USA, Pathways between Socioeconomic Status and Behavioral Cancer Risk Factors Study (PATHS)	Community dwelling adults	EMA	Multi	EMA	Past 24-h (i.e. previous day)	SB	ActiGraph GT3X	ST	7 days	NR	Vertical	Dominant hip
							NHANES	Multi	Quest.	Usual week	TV and computer use weekday and weekend						
							IPAQ-LF (sitting questions only)	Single (weekday + weekend day)	Quest.	Past 7 days	Sitting						
Kohler, 2017	Validity = 50 (13), reliability = 52 (13)	Validity = 49, reliability, reliability = 67	Validity = 24 (49%), reliability = 33 (49%)	Validity = 25 (51%), reliability = 34 (51%)	Germany, German National Cohort pilot study	Healthy, community dwelling adults	cpar24	N/A	24-h recall	Previous 24 hours	SBs	ActiGraph GT3X	ST	3 days	< 100 cpm	NR	NR
Kozey-Keadle, 2012	46.5 (10.8)	13	NR	NR	USA, NR	Overweight, non-exercising	IPAQ-SF	Single	Quest.	Typical workday in last 7 days	Sitting	activPAL	Sitting	7 days	N/A	N/A	Right thigh

First Author, year	Age range or mean (SD)	N analyzed (%)			Country, Study name	Population	Self-report measure					Device measure					
		Total	Men	Women			Name	Multi-item or single	Measure type	Recall period*	Domain or type	Name	Domain or type	Wear time	Cut-point	Axis	Wear location
						office workers	Domain-Specific Quest. (D-SQ)	multi		Previous 7 days	Travel, TV, occup., computer at home, leisure time not including TV	ActiGraph GT3X	ST		100 & 150 cpm	NR	Right hip
Kozo, 2012	45.1 (11.0)	2199	1139 (51.8%)	1058 (48.2%)	USA, Neighborhood Quality of Life Study	Community dwelling adults	IPAQ-LF (sitting questions only)	Single (weekday + weekend day)	Quest.	Previous 7 days	Sitting	ActiGraph 7164 & 71256	ST	7 days	< 100 cpm	Vertical	Waist
Laeremans, 2017	35 (10)	122	45%	55%	Spain, England, Belgium, FP7 PASTA Project (Physical Activity through Sustainable Transport Approaches)	Healthy adults	GPAQ	Single	Quest.	Typical day	Sitting	SenseWear	ST	7 days	N/A	N/A	Left arm
Lagersted-Olsen, 2014	40.9 (8.6)	26 (work sitting = 25, leisure time on workday = 26, leisure day = 26)	14	12	Denmark, NR	Office workers	New questions - unnamed but based on IPAQ and MOSPAQ	Multi	Quest.	Past 7 days	Occup. sitting	ActiGraph GT3X+	ST at work	7 days	N/A - used inclinometer	N/A	Right thigh
Larsson, 2019	Validity = 42.9 (8.9), reliability = 70.3 (5.0)	Validity = 284, reliability = 95	Validity = 95, reliability = 29	Validity = 188, reliability = 66	Sweden, Validity = Physical Activity and Healthy Brain Functions, reliability = Health Project at GIH	Validity = office workers, reliability = seniors	SED-GIH	Single	Quest.	Normal day	Sitting	activPAL3 micro	Sitting/ lying	7 days	N/A	N/A	Right thigh

First Author, year	Age range or mean (SD)	N analyzed (%)			Country, Study name	Population	Self-report measure					Device measure					
		Total	Men	Women			Name	Multi-item or single	Measure type	Recall period*	Domain or type	Name	Domain or type	Wear time	Cut-point	Axis	Wear location
Legge, 2017	SLE = 43.9 (12.5), RA = 51.5 (13.4), control = 50.9 (11.2)	SLE = 20, arthritis = 19, healthy controls = 20	SLE = 2 (10%), RA = 8 (42%), Control = 7 (35%)	SLE = 18 (90%), RA = 11 (58%), control = 13 (65%)	Canada, NR	Patients with systemic lupus erythematosus	Framingham Physical Activity Index	Single (working and leisure hours)	Quest.	Unclear	SBs	ActiGraph GT3X+	ST	7 days	< 100 cpm	NR	Waist
Lewis, 2016	Men: 65.4 (5.7); Women: 72.4 (6)	27	10	17	Australia, Small Steps	Older adults	MARCA	Multi	Quest.	Previous day	SBs	activPAL3	Sitting time	7 days	N/A	N/A	Thigh
Lewis, 2018	67.9 (6.6)	52	32	20	UK, NR	Older cancer survivors and adults at risk of cancer	IPAQ-LF (sitting questions only)	Single (weekday + weekend day)	Quest.	Past 7 days	Sitting	ActiGraph GT3X	ST	7 days	< 100 cpm	VM	Right hip
Libertine, 2011	45.6 (11.28)	19	NR	NR	USA, NR	Overweight and obese office workers	Sitting Quest.<br>Focus Quest.	NR	Quest.	NR	Sitting	activPAL	Sitting/ lying	2 weeks	N/A	N/A	NR
Lopez-Rodriguez, 2017	71.96 (5.48)	73	11	62	Spain, NR	Healthy elderly	EXERNET Quest.	Single sedentary question	Quest.	Past 7 days	Sitting	ActiGraph GT1M	ST	7 days	≤ 100 cpm	Vertical	Right hip
Loprinzi, 2013	21.4 (2.1)	87 (78 accelerometer)	58.6%	41.3%	USA, NR	Healthy young adults	SBQ	Multi	Quest.	Typical weekday and weekend day	ST	ActiGraph GT1M	ST	7 days	< 100 cpm	Vertical	Right hip
Lucas, 2013	NR	60	37%	63%	USA, NR	College freshmen	IPAQ-LF (sitting questions only)	Single (weekday + weekend day)	Quest.	Last 7 days	Sitting	activPAL	Sitting/ lying	7 days	N/A	N/A	NR
Mader, 2006	Validation men = 57.6 (14.4), women =	Validity = 35, reliability = 178	Validation = 22, reliability = 101	Validation = 13, reliability = 77	Switzerland, NR	Middle-aged women & men	IPAQ-SF	Single	Quest.	Last 7 days	Sitting	ActiGraph AM7164	ST	7 days	< 100 cpm	Vertical	Hip

First Author, year	Age range or mean (SD)	N analyzed (%)			Country, Study name	Population	Self-report measure					Device measure					
		Total	Men	Women			Name	Multi-item or single	Measure type	Recall period*	Domain or type	Name	Domain or type	Wear time	Cut-point	Axis	Wear location
	49.7 (14.0). Reliability men = 46.8 (13.2), women = 46.1 (14.8)																
Maher, 2017	74.2 (8.2)	100	33	67	USA, NR	Community-dwelling older adults	Daily diary	N/A	Diary	Current day	Sitting/ lying	activPAL	Sitting/ lying	14 days	N/A	N/A	Thigh
Marmeleira, 2013	47.7 (11.3)	58	37	21	Portugal, NR	Adults who are blind	IPAQ-SF	Single	Quest.	Last 7 days	Sitting	ActiGraph GT1M	ST	7 days	< 100 cpm	Vertical	Right hip
Marshall, 2010	NR	Validity = 55	Validity = 11, reliability = 96	Validity = 44, reliability = 157	Australia, NR	Middle-aged women & men	Marshall Sitting Quest.	Multi	Quest.	Usual weekday and weekend day	Sitting (travel, work, TV, computer, leisure)	ActiGraph GT1M	ST	7 days	< 100 cpm	Vertical	NR
Marshall, 2015	83.5 (6.5)	230	69 (30%)	161 (70%)	USA, NR	Community-dwelling older adults	Modified SBQ	Multi	Quest.	Typical weekday and weekend day	ST: TV, computer/ internet use, reading, socializing, driving or using motorized travel, doing hobbies, office or volunteer work, napping, and other behaviors	ActiGraph GT3X+	ST	7 days	< 100 cpm	NR	Right hip
Matsuo, 2016	men: 46.3 (8.0); women: 35.8 (7.5)	62	36	26	Japan, NR	Workers	Workers' Sitting- and Walking-Time Quest. (WSWQ)	Multi	Quest.	Typical day	Occup. sitting, leisure sitting, non-	activPAL	Sitting/ lying	7 days	N/A	N/A	Thigh

First Author, year	Age range or mean (SD)	N analyzed (%)			Country, Study name	Population	Self-report measure					Device measure					
		Total	Men	Women			Name	Multi-item or single	Measure type	Recall period*	Domain or type	Name	Domain or type	Wear time	Cut-point	Axis	Wear location
											workday sitting						
Matsuzaki, 2016	35.0 (14.2)	245	136 (56%)	109 (44%)	India, Hyderabad DXA Study	General population	Andhra Pradesh Children and Parent Study Physical Activity Quest.	Multi	Quest.	Last 7 days	Multiple SBs	Actiheart	ST	8 days	N/A	N/A	Chest
Matthews, 1995	Men: 25.5 (3.94), women: 28.2 (6.85)	25	14	11	USA, NR	University students	PAL	N/A	3-day Log	Current day	ST	Tritrac R3D	ST	7 days	N/A	Tri-axial	Right hip
Matthews, 2013	41.3 (14.8)	88	40	48 (53.7%)	USA, NR	Middle-aged women & men	PDR	N/A	Quest.	Past day	ST	activPAL	Sitting/ lying	7 days	N/A	N/A	Right thigh
												ActiGraph GT3X+	ST		< 100 cpm	Vertical	Right hip
Matthews, 2018	63.2 (5.9)	activPAL = 932, AARP = 842, ACT24 = 923, ActiGraph = 924	461	471	USA, Interactive Diet and Activity Tracking in AARP	General population	AARP Quest., 24-hour recall (ACT24)	AARP = single, ACT24 = n/A	AARP = Quest., ACT24 = recall	AARP = past 12 months, ACT24 = 24-h recall	ST	activPAL	Sitting/ lying	7 days	N/A	N/A	Right thigh
												ActiGraph GT3X+	ST		< 100 cpm	Vertical	Right hip
Matthews, 2011	48.3 (12.01)	45	17	28	Scotland, NR	Adults with intellectual disabilities	IPAQ-SF	Single	Quest.	Past 7 days	Sitting	ActiGraph GT1M	ST	7 days	NR	Vertical	NR
Matton, 2007	Employed/ unemployed = 22-61, retired = 48-78	Employed/unemployed: reliability = 66, validity = 62, retired: reliability = 36, validity = 49	Employed/ unemployed: reliability = 31, validity = 32, retired: reliability = 20,	Employed/ unemployed: reliability = 35, validity = 30, retired: reliability = 16, validity = 19	Belgium, NR	Employed/ unemployed adults, retired adults	FPACQ	Single	Quest.	Usual week	TV	Triaxial RT3	TV time (overlaid from log)	7 days	NR	VM	Right hip

First Author, year	Age range or mean (SD)	N analyzed (%)			Country, Study name	Population	Self-report measure					Device measure					
		Total	Men	Women			Name	Multi-item or single	Measure type	Recall period*	Domain or type	Name	Domain or type	Wear time	Cut-point	Axis	Wear location
			validity = 30														
Mazzoni, 2017	58 (11)	42	0	42	Sweden, Physical Training and Cancer	Breast cancer patients	Log book	N/A	Log book	Current day	ST	SenseWear Armband Mini	ST	7 days	≤ 1.5 MET	N/A	Arm (right or left)
McNeil, 2018	59 (5)	Accelerometer: high = 165, moderate = 156, self-report: high = 180, moderate = 175	0	Accelerometer: high = 165, moderate = 156, self-report: high = 180, moderate = 175	Canada, Breast Cancer and Exercise Trial in Alberta	Post-menopausal women	SIT-Q	Multi	Quest.	Usual past 12 months	Total, occup. and leisure ST	ActiGraph GT3X+	ST	7 days	< 100 cpm	Vertical	Waist
Melville, 2011	48.3 (12.01)	Accelerometer = 45, IPAQ = 47	NR	NR	Scotland, TAKE5 intervention	Adults with intellectual disabilities and obesity	IPAQ-SF	Single	Quest.	Last 7 days	Sitting	ActiGraph GT1M	ST	7 days	< 500 cpm	Vertical	Hip
Menezes, 2017	43.22 (13.9)	49	17	32	Portugal, NR	People who are deaf	IPAQ-SF	Single	Quest.	Last 7 days	Sitting	ActiGraph GT1M	ST	7 days	< 100 cpm	Vertical	Right hip
Mensah, 2016	40.5 (14.3)	88 (total validity), 82 (domain validity), 32 (reliability)	45	51	France, NR	Healthy general population	STAQ	Multi	Quest.	Last 4 weeks	Total sitting: occup., travel	ActiGraph GT3X+	ST	7-14 days	< 150 cpm	NR	Hip
Metcalf, 2018	Training = 49.1 (14.1), testing = 50.6 (13.7), validation = 36.5 (8.1)	Training = 86, testing = 22, validation = 120	Training = 32.6%, testing = 27.3%, validation = 7.5%	Training = 67.4%, testing = 72.7%, validation = 92.5%	USA, Active Ottumwa	General population	GPAQ	Single	Quest.	Past 7 days	Sitting	ActiGraph GT9X	ST	7 days	Staudenmayer decision tree algorithm	N/A	Non-dominant wrist
Meyer, 2015	38.6 (14.0)	24	0	24	USA, NR	Patients with depression	IPAQ-LF (sitting questions only)	Single (weekday + weekend day)	Quest.	Last 7 days	Sitting	ActiGraph GT3X+	ST	7 days	< 1.5 METS	VM	Waist (either side)

First Author, year	Age range or mean (SD)	N analyzed (%)			Country, Study name	Population	Self-report measure					Device measure					
		Total	Men	Women			Name	Multi-item or single	Measure type	Recall period*	Domain or type	Name	Domain or type	Wear time	Cut-point	Axis	Wear location
Moran, 2016	Assisted living: 89.2 ± 6.8, independent living: 78.9 ± 5	40 (20 assisted living (14 accel data), 20 independent living (18 with accel data)	13	27	Australia, NR	Older adults	IPAQ-SF	Single	Quest.	Last 7 days	Sitting	SenseWear Armband	ST	7 days	< 1.5 METS	N/A	Arm
Moss, 2018	39.6 (9.1)	56	28	28	South Africa, NR	People with intellectual disabilities	IPAQ-SF (proxy reported by caregivers)	Single	Quest.	Last 7 days	Sitting	ActiHeart	ST	7 days	< 1.5 METS	N/A	NR
Mumu, 2017	35 (9)	155	74	88	Bangladesh, NR	Healthy adults	GPAQ	Single	Quest.	Usual day	Sitting	ActiGraph GT3X+	ST	7 days	< 100 cpm	Vertical	Left hip
Murillo-Rabago, 2010	30	43	44%	56%	Mexico, NR	Healthy adults	IPAQ-SF & IPAQ-LF (questions NR)	Single	Interview	Last 7 days	Sitting	ActiGraph GT1M	ST	7 days	NR	Vertical	NR
O'Neill, 2017	63 (10)	55	22	33	Northern Ireland, NR	Patients with Bronchiectasis	IPAQ-LF (sitting questions only)	Single (weekday + weekend day)	Quest.	Last 7 days	Sitting	ActiGraph GT3X	ST	7 days	< 100 cpm	NR	Dominant hip
Oostdam, 2013	31.4 (3.9)	55	0	55	Netherlands, FitFor2-study	Overweight and obese pregnant women	AQuAA	Multi	Quest.	Past week	SBs	Actitrainer	ST	4 days	< 700 cpm	Vertical	Waist
Otten, 2010	41.4 (13.0)	40	32%	68%	USA, NR	Overweight and obese adults	None	Single	Quest.	Average day	TV	TV monitoring device	TV time	3 weeks	N/A	N/A	N/A
Oviedo-Caro, 2018	32.5 (4.4)	186	0	186	Spain, NR	Pregnant women	SBQ	Multi	Quest.	Typical weekday and weekend day	SBs	SenseWear Mini armband	ST	7 days	≤ 1.5 MET	N/A	Arm
Oyeyemi, 2014	32.6 (9.9)	144	86	58	Nigeria, NR	General population	Hausa IPAQ-SF	Single	Quest.	Last 7 days	Sitting (weekday)	ActiGraph 7164	ST	7 days	< 100 cpm	Vertical	Right hip

First Author, year	Age range or mean (SD)	N analyzed (%)			Country, Study name	Population	Self-report measure					Device measure					
		Total	Men	Women			Name	Multi-item or single	Measure type	Recall period*	Domain or type	Name	Domain or type	Wear time	Cut-point	Axis	Wear location
Pedersen, 2016	Validity = 45.62 (10.96), reliability = 42.87 (11.34)	Validity = 34 (cohort 1), reliability = 75 (cohort 3)	Validity = 6, reliability = 13	Validity = 28, reliability = 62	Australia, NR	Desk-based workers	OSPAQ	Multi	Quest.	Typical workday in last 7 days	Occup. sitting	activPAL	Sitting	1 8-h workday	N/A	N/A	Right thigh
Pedisic, 2014	>18 years	Validity = 143, reliability = 96	Validity = 37.4%, reliability = 37.5%	Validity = 62.6%, reliability = 62.5%	Australia, NR	Desk-based workers	SITBRQ	Multi	Quest.	Typical workday	Duration and frequency of sitting breaks at work	ActiGraph GT1M	Duration and frequency of sitting breaks at work	7 days	< 100 cpm	Vertical	Right hip
Peters, 2010	53.4 (9.4)	545	271	274	China, Shanghai Women's Health Study and Shanghai Men's Health Study	Urban adults	PYPAQ	Multi	Quest.	Past year	SBs	ActiGraph 7164	ST	7 days	< 100 cpm	Vertical	Left hip
Pinto, 2017	55.62 (9.55)	76	0	76	USA, NR	Breast cancer survivors	7-d PAR	N/A	Daily recall	Previous 7 days	Sitting	ActiGraph GT3X	ST	7 days	< 100 cpm	NR	NR
Powell, 2011	39	41	NR	NR	USA, NR	Overweight/ obese Latina immigrants	GPAQ	Single	Quest.	Typical day	Sitting	NR	ST	3 week days, 1 week end day	NR	Vertical	NR
Prince, 2018	43 (12)	313	16 (5%)	297 (95%)	Canada, Champlain Nurses' Study	Nurses	IPAQ-SF	Single	Quest.	Last 7 days	Sitting	ActiGraph GT3X+	ST	7 days	< 150 cpm	VM	Right hip
							SBQ	Multi		Usual weekday and weekend day	SBs						
Prince, 2019	61.5 (9.8)	35	19	16	Canada, SIT-CR study	Cardiac rehabilitation patients	IPAQ-SF	Single	Quest.	Last 7 days	Sitting	activPAL3	Sitting + lying	7 days	N/A	N/A	Right thigh

First Author, year	Age range or mean (SD)	N analyzed (%)			Country, Study name	Population	Self-report measure					Device measure					
		Total	Men	Women			Name	Multi-item or single	Measure type	Recall period*	Domain or type	Name	Domain or type	Wear time	Cut-point	Axis	Wear location
Rees-Punia, 2019	51.7 (range: 31-72)	713	290	423	USA, Cancer Prevention 3 Study	General population	7-day diary	N/A	Diary	Current day	Sitting	ActiGraph GT3X	ST	7 days	NR	Tri-axial	Non-dominant hip
							Sitting Quest.	Multi	Quest.	Past year typical day							
Riviere, 2018	30.1 (10.7)	87	NR	NR	France, NR	General population	GPAQ (French version)	Single	Quest.	Usual day	Sitting	ActiGraph GT3X	ST	7 days	NR	NR	Waist
							IPAQ-LF (French version, sitting questions only)	Single (weekday and weekend day)		Last 7 days							
Roman-Vinas, 2010	43.2 (14.1)	Validity = 54, reliability = 66	Validity = 23, reliability = 27	Validity = 31, reliability = 39	Spain, NR	General population	IPAQ-LF (sitting questions only)	Single (weekday + weekend day)	Quest.	Last 7 days	Sitting	ActiGraph MTI	ST	7 days	<101 cpm	Vertical	Waist
Rosenberg, 2010	Validity: women = 41.2 (8.7), men = 43.9 (8.0), Reliability = 20.4 (1.3)	Validity = 842, reliability = 49	Validity = 441	Validity = 401	USA, NR	Validity = overweight men & women	SBQ	Multi	Quest.	Usual weekday and weekend day	SBs	ActiGraph 7164	ST	7 days	< 100 cpm	Vertical	Right hip
Rosenberg, 2016	83.6 (6.4)	307, self-report: 280, accel: 302	123	222	USA, NR	Older adults in retirement communities	SBQ	Multi	Quest.	Usual weekday and weekend day	SBs	ActiGraph GT3X	ST	6 days	< 100 cpm	NR	Right hip
Rosenberg, 2015	71.4 (6.4)	23 (ActiGraph 22)	30%	70%	USA, Take Active Breaks from Sitting Pilot Study	Overweight and obese older adults	IPAQ-LF (sitting questions only)	Single (weekday + weekend day)	Quest.	Last 7 days	Sitting	activPAL	Sitting/ lying	7 days	N/A	N/A	Thigh
												ActiGraph GT3X+	ST		< 100 cpm	NR	Waist
Ruiz-Casado, 2016	54 (11)	177	36%	64%	Spain, NR	Spanish cancer survivors	IPAQ-SF	Single	Quest.	Last 7 days	Sitting	ActiGraph GT3X	ST	5-10 days	< 100 cpm	NR	NR
							GPAQ			Typical day							

First Author, year	Age range or mean (SD)	N analyzed (%)			Country, Study name	Population	Self-report measure					Device measure					
		Total	Men	Women			Name	Multi-item or single	Measure type	Recall period*	Domain or type	Name	Domain or type	Wear time	Cut-point	Axis	Wear location
Ruiz-Casado, 2015	18-65	139	NR	NR	Spain, NR	Spanish cancer survivors	IPAQ-SF	Single	Quest.	Last 7 days	Sitting	ActiGraph GT3X	ST	3 week days, 2 week end	NR	NR	NR
Ryan, 2014	Middle-aged: included = 54.7 (5.8), excluded = 54.2 (5.7), older/included = 73.8 (6.5), excluded = 75.8 (7.7)	Middle-aged/included = 3332, excluded = 1694, older/included = 2022, excluded = 1449	Middle-aged/included = 45.4%, excluded = 45.6%, older/included = 46.3%, excluded = 40.6%	Middle-aged/included = 54.6%, excluded = 54.4%, older/included = 53.7%, excluded = 59.4%	England, Health Survey for England	General population	Health Survey for England	Single (week day and weekend)	Quest.	NR	Sitting	ActiGraph GT1M	ST	1 week	NR	Vertical	NR
Ryan, 2018	73.7 ± 6.3 years	Reliability = 73, validity = 86	Reliability =41	Reliability = 48	England, NR	Community dwelling older adults	IPAQ-LF (sitting and sitting + motorized)	Single (weekday + weekend day), multi	Quest.	Last 7 days	Sitting with and without motorized transport	GeneActiv Original	ST	7 days	NR	Tri-axial	Thigh
Ryde, 2017	56.5 (17.7)	Self-report = 90, accelerometer = 29	0	Self-report = 90, accelerometer = 29	Scotland, Well!Bingo project	Women attending Bingo club	Marshall Sitting Quest.	Multi	Quest.	Previous week	Total sitting: travel + work + TV + home computer use + leisure time (excluding TV)	ActiGraph GT3X	ST	7 days	< 100 cpm	Vertical	Waist
Sasaki, 2018	56.6 (7.3)	63	15	48	USA, NR	People with Multiple Sclerosis	Marshall Sitting Quest.	Multi	Quest.	Usual weekday and weekend day	Total sitting: travel + work + TV + home computer use + leisure time	ActiGraph GT3X	ST	7 days	< 100 cpm	NR	Right hip

First Author, year	Age range or mean (SD)	N analyzed (%)			Country, Study name	Population	Self-report measure					Device measure					
		Total	Men	Women			Name	Multi-item or single	Measure type	Recall period*	Domain or type	Name	Domain or type	Wear time	Cut-point	Axis	Wear location
											(excluding TV)						
Schaller, 2016	30.7 (15.3)	78: low back patients = 27, healthy controls = 53	47	31	Germany, Movement Coaching study and the Make Move study	Low back pain patients & healthy controls	GPAQ	Single	Quest.	Last 7 days	Sitting	ActiGraph GT3X	ST	7 days	< 100 cpm	NR	Right waist
Scheers, 2012	41.4 (9.8)	Valid SenseWear = 405, SWD = 383	212	230	Belgium, NR	Workers	FPACQ	Multi	Quest.	Usual week	Sitting	SenseWear Pro 3	ST	7 days	N/A	N/A	Right arm
Segura-Jimenez, 2013	51.1 (8.2)	Validity = 123, reliability = 183	0	Validity = 123, reliability = 183	Spain, the Andalus project	Women with fibromyalgia	Modified IPAQ-LF (Spanish, sitting/lying and sitting/lying + motorized)	Multi: sitting (weekday + weekend + transportation)	Quest.	Last 7 days	Sitting/lying, Sitting + travel	SenseWear Pro 3	ST	7 days (9 remove first and last day)	< 3 METS	Biaxial	Arm
Shadyab, 2017	79.2 (6.7)	Accelerometer-measured (n = 1,297) or self reported (n = 1,383)	0	Accelerometer-measured (n = 1,297) or self reported (n = 1,383)	USA, Women's Health Initiative	Older women	No name	Multi	Quest.	Usual day	ST (sitting + lying)	ActiGraph GT3X	ST	7 days	≤ 72 cpm	VM	Right hip
Shuval, 2014	40-79	151	NR	NR	USA, Rapid Assessment Disuse Index (RADI) study	Primary care patients	Rapid Assessment Disuse Index (RADI)	Single	Matrix Quest.	Past week/ Past Month/ Past Year	Sitting: at home/ work, TV/video/ DVDs, computer at home/ work, eating meals, etc.	ActiGraph GT3X	ST	7 days	< 100 cpm	NR	Right hip

First Author, year	Age range or mean (SD)	N analyzed (%)			Country, Study name	Population	Self-report measure					Device measure					
		Total	Men	Women			Name	Multi-item or single	Measure type	Recall period*	Domain or type	Name	Domain or type	Wear time	Cut-point	Axis	Wear location
Simpson, 2015	Validity = 44.1 (16.1), reliability = 44.7 (16.3)	Validity = 419, reliability = 217	Validity = 206, reliability = 106	Validity = 213, reliability = 111	USA, The Effects o f Statins on Muscle Performance	Healthy adults	Question 8 of the PPAQ	Multi	Quest.	Typical day	Sitting	Actical	ST	4 days	NR	Omni	Dominant hip
Stolberg, 2018	42.3 (9.1)	60	18	42	Denmark, NR	Patients undergoing gastric bypass	Recent Physical Activity Quest.	Multi	Quest.	Previous 4 weeks	SBs	ActiGraph GT3X	ST	7 days	< 100 cpm	Vertical	Hip
Sudholz, 2018	32.1 (9.9)	ActivPAL = 52, ActiGraph = 49	ActivPAL = 30, ActiGraph = 20	ActivPAL = 22, ActiGraph = 29	Australia, NR	Workers	None	Single	Quest.	Previous 7 days	Sitting at work	activPAL + log book	Sitting time	8 days	N/A	N/A	Left thigh
												ActiGraph GT3X + log book	ST at work		< 100 cpm	NR	Right hip
Sushames, 2015	Indigenous: 35.6 (10.9), non-Indigenous: 32.9 (11.5)	Indigenous: 61, non-Indigenous: 36	NR	NR	Australia, NR	Indigenous Australians	Past Day Adults' ST Quest.	Multi	Quest.	Previous day	SBs	ActiGraph GT3X	ST	7 days	< 100 cpm	NR	Hip
Sweatt, 2015	36.6 (7.8)	44	0	44	USA, NR	Latina Immigrants	GPAQ	Single	Quest.	Typical week	Sitting	ActiGraph MTI	ST	4 days	NR	Vertical	Right hip
Terada, 2015	Men: 22.3 (1.8), women:21.6 (1.0)	19	11	8	Canada, NR	General population	Activity Log Diary	N/A	Diary	Current day	SBs	SenseWear Pro 3	ST	3 days	≤ 1.5 METS	N/A	Right arm
Toledo, 2017	49.0 (8.9)	28	85%	15%	USA, NR	US veterans & university employees	BeWell24 App	N/A	EMA	5-minute recall	SBs	activPAL3	Sitting/ lying	7 days	N/A	N/A	Right thigh
Umstattd Meyer, 2013	44.6 (10.9)	87	22	78%	USA, NR	Free living adults	IPAQ (version NR)	Single	Quest.	Last 7 days	Sitting	ActiGraph GT1M	ST	7 days	< 100 cpm	Vertical	Non-dominant hip
Unick, 2017	27.7 (4.4)	595	22%	78%	USA, Study of Novel Approaches to Weight Gain	Young adults	SBQ	Multi	Quest.	Usual weekday and weekend	SBs	SenseWear Armband	ST	7 days	< 1.5 METs	Biaxial	Arm

First Author, year	Age range or mean (SD)	N analyzed (%)			Country, Study name	Population	Self-report measure					Device measure					
		Total	Men	Women			Name	Multi-item or single	Measure type	Recall period*	Domain or type	Name	Domain or type	Wear time	Cut-point	Axis	Wear location
					Prevention trial												
Urda, 2017	48 (10)	44	0	44	USA, NR	Office workers	PPAQ	Multi	Quest.	Typical weekday and weekend day in previous 7 days	Total ST (sitting/ lying/ sleeping)	activPAL3	Sitting/ lying	7 days	N/A	N/A	Thigh
							OSPAQ	Multi	Quest.	Previous 7 days	Occup. sitting						
Van Cauwenberg, 2014	74.2 (6.2)	Validity = 442, reliability = 28	45.2%	54.8%	Belgium, NR	Free-living older adults	NR	Multi	Quest.	Last 7 days	ST: TV, computer, reading, hobbies, seated conversation, listening to music, telephone use, public transport, driving a car, being passenger in a car, sitting during household chores, resting, eating	ActiGraph GT3X	ST	7 days	< 100 cpm	NR	Right hip
Van Der Ploeg, 2010	≥18	Reliability = 134, validity = 129	79	55	Australia, NR	Workers	2006 Australian Bureau of Statistics time use survey	N/A	Time use diary	Current day	SBs	ActiGraph GT1M	ST	10 days	< 100 cpm	Vertical	Right hip
Van Dyck, 2015	43.5 (12.3)	542	45.2%	54.8%	Belgium, BEPAS	General population	IPAQ-LF (sitting	Single (weekday +	Quest. (interview)	Last 7 days	Sitting	ActiGraph 7164	ST	7 days	< 100 cpm	Vertical	Right hip

First Author, year	Age range or mean (SD)	N analyzed (%)			Country, Study name	Population	Self-report measure					Device measure					
		Total	Men	Women			Name	Multi-item or single	Measure type	Recall period*	Domain or type	Name	Domain or type	Wear time	Cut-point	Axis	Wear location
							questions only)	weekend day)									
Van Dyck, 2010	42.7 (12.6)	1200	47.9%	52.1%	Belgium, BEPAS	General population	IPAQ-LF (sitting questions only)	Single (weekday + weekend day)	Quest.	Last 7 days	Sitting	ActiGraph 7164	ST	7 days	< 100 cpm	Vertical	Right hip
van Nassau, 2015	38 (11)	34, ActiGraph = 34, activPAL = 35	NR	36 (86%)	Australia, Stand@work trial	Health agency employees	OSPAQ	Multi	Quest.	Average workday and non-work day on last 7 days	Workplace sitting	activPAL3	Sitting at work	Work time during 1 work week	N/A	N/A	Thigh
		33, ActiGraph=34, ActivPAL = 35					WSQ	Single		Typical workday in last 7 days	Occup. sitting	ActiGraph GT1M & GT3X	ST at work		< 100 cpm	Vertical	Right hip
Vandezande, 2014	41.2 (10.9)	298	70 (23.5%)	228 (76.5%)	USA, NR	Overweight and obese adults	SB Quest.	Multi	Quest.	Typical weekday and weekend	Sum of SBs	ActiGraph	ST	7 days	< 100 cpm	NR	Right hip
Vandoni, 2017	median = 18	30	24	6	Italy, NR	High school students (median age = 18)	IPAQ-LF (sitting questions only)	Multi (week day + weekend day sitting incl. TV)	Quest.	Last 7 days	Sitting	ActiHeart	ST	5 days	< 100 cpm	Omni	NR
Vanroy, 2014	68 (11)	16	9 (56.2%)	7 (43.8%)	Belgium, NR	Stroke patients	Activity Diary	N/A	Activity Diary	Current day	SBs (≤ 1 MET)	SenseWear Pro2 Armband	ST	1 day	N/A	N/A	Right arm
Veitch, 2018	NR	64	NR	NR	Australia, NR	Orthopedic trauma patients	IPAQ (version NR)	Single	Quest.	Last 7 days	Sitting	activPAL	Sitting	10 days	N/A	N/A	NR
							AusDiab3	Multi		Past 7 days	SB						
Visser, 2013	65-92, 74.3 (6.9)	validity = 83, reliability = 63	50.6%	49.4%	Netherlands, Longitudinal Aging Study Amsterdam	Older adults	LASA SB Quest.	Multi	Quest.	Usual weekday and	Sum of SBs: napping, reading,	ActiGraph GT3X	ST	8 days	<100 cpm	NR	Waist

First Author, year	Age range or mean (SD)	N analyzed (%)			Country, Study name	Population	Self-report measure					Device measure					
		Total	Men	Women			Name	Multi-item or single	Measure type	Recall period*	Domain or type	Name	Domain or type	Wear time	Cut-point	Axis	Wear location
										weekend day	listening to music, TV, computer, working, hobby, talking, travel, church/ theater						
Wanner, 2017	47.0 (15.0)	354	175	179	Switzerland, Swiss Food Panel	General population	GPAQ (German, French, Italian versions)	Single	Quest.	Usual day, typical week	Sitting	ActiGraph GT3X	ST	7 days	< 100 cpm	NR	Right hip
Wanner, 2016	18+ Mean = 54.6, SD = NR	346	157 (45.4%)	189 (54.6%)	Switzerland, Swiss Cohort Study on Air Pollution and Lung and Heart Disease in Adults	General population	IPAQ-LF (sitting + motorized))	Multi (weekday + weekend day sitting + motorized travel)	Quest.	Last 7 days	Sitting + motorized transport	ActiGraph GT3X	ST	8 days	< 150 cpm	NR (suspected VM)	Right hip
Waters, 2016	43 ± 9	37	16	24	Singapore, SB in Business School Employees study	Office workers	SB in Business School Employees Quest.	Multi	Quest.	Typical day	Sum of SBs: work, transport, leisure including TV, napping, eating, online, other	ActiGraph GT3X	ST	1 week	< 150 cpm	Vertical	Right hip
Watson, 2017	29.5 (5.7)	95 (accelerometer = 89, GPAQ = 91)	0	95 (accelerometer = 89, GPAQ = 91)	South Africa, Soweto First 1000 Days Cohort	Pregnant women	GPAQ	Single	Quest.	Typical day	Sitting	ActiGraph GT3X	ST	7 days	< 100 cpm	NR	Right hip
Welch, 2018	56.8 (9.2)	414	0	414	USA, NR		IPAQ-LF (sitting	Single (weekday +	Quest.	Last 7 days	Sitting	ActiGraph GT1M	ST	7 days	< 100 cpm	Vertical	Right hip

First Author, year	Age range or mean (SD)	N analyzed (%)			Country, Study name	Population	Self-report measure					Device measure					
		Total	Men	Women			Name	Multi-item or single	Measure type	Recall period*	Domain or type	Name	Domain or type	Wear time	Cut-point	Axis	Wear location
						Breast cancer survivors	questions only)	weekend day)									
							Sitting Time Quest.	Multi			Sum of SBs						
Whitfield, 2013	34.7 (7.7)	MSTQ = 25, Accelerometer = 21 (workday), 16 (non-workday)	44%	56%	USA, NR	Recreational runners	Multicontext Sitting Time Quest.	Multi	Quest.	Usual week (work day and non-work day)	Weekend & week day sitting across contexts	ActiGraph GT1M	ST	1 work day, 1 non-work day	< 100 cpm	Vertical	Right hip
Wick, 2016	40.8 (11.4)	38	8	30	Switzerland, NR	Office workers	OSPAQ	Multi	Quest.	Last 7 days	Occup. sitting	ActiGraph GT3X + work log	ST	1 work week	Sitting time from inclinometer	N/A	Right thigh
Wijndaele, 2014	Sample 1: 39.4 (11.1)	Validity: 49 (total ST), reliability: 48 (total ST)	Validity: 41%	Validity: 59%	Belgium, NR	General population	SIT-Q-7d	Multi	Quest.	Last 7 days	Sitting: meals, travel, occup. nonoccup. screen time, and other ST	activPAL3	Sitting	7 days	N/A	N/A	Thigh
	Sample 2: 49.6 (7.3)	Validity: 402, reliability: 237 (total ST	Validity: 193	Validity: 209	UK, NR							Actiheart	ST	6 days		N/A	NR
Yi, 2014	NR	679	NR	NR	USA, 2011 Physical Activity and Transit Survey	General population	Physical Activity Transit Survey	Multi	Quest.	NR	Daytime and evening sitting	NR (accelerometer)	ST	7 days	< 100 cpm	NR	NR
Yu,2014	NR	68	26	42	UK, NR	Rheumatoid arthritis patients	IPAQ (version NR)	Single	Quest.	Last 7 days	Sitting	ActiGraph	ST	7 days	NR	NR	NR

AQuAA – Activity Questionnaire for Adults and Adolescents, AusDiab3 – Australian Diabetes, Obesity and Lifestyle General Questionnaire 3, AWAS – Australian Women's Activity Survey, BEPAS – Belgian Environment and Physical Activity Study, CHAMPS – Community Health Activities Model Program for Seniors, cpm – counts-per-minute, EMA – ecological momentary assessment, FPACQ – Flemish physical activity

computerized Questionnaire, IPAQ – International Physical Activity Quest., IPAQ-LF – International Physical Activity Questionnaire-Long Form, IPAQ-SF – International Physical Activity Questionnaire-Short form, MARCA – Multimedia Activity Recall for Children and Adolescents, N/A – not applicable, NQLS – Neighborhood Quality of Life Study, NR– not reported, NS – not stated, occup. – occupational, OSPAQ – Occupational Sitting and Physical Activity Questionnaire, PAL – Physical Activity Log, PAPQ – physical activity and pregnancy Questionnaire, PAST-U – Past-day Adults' Sedentary Time-University, PDR – Previous Day Recall, PPAQ – Paffenbarger Physical Activity Questionnaire, PYPAQ – Past Year Physical Activity Questionnaire, Quest. – questionnaire, SITBRQ – Workplace Sitting Breaks Questionnaire, SIT-Q-7d – last 7-day sedentary behavior Questionnaire, ST – sedentary time, STAQ – Sedentary, Transportation and Activity Questionnaire, UK – United Kingdom, USA - United States of America, VM – vector magnitude, WSQ – Workforce Sitting Questionnaire, x – vertical axis, YPAS – Yale Physical Activity Survey for Older Adults

*Note: The IPAQ-LF is a single sitting item for weekdays and weekend days separately unless otherwise stated. The IPAQ-LF was assumed to reference the last 7 days unless otherwise specified. The GPAQ was assumed as a typical day in a usual week unless otherwise specified.
