# Supplementary material for: A comparison of self-reported and device measured sedentary behaviour in adults: a systematic review and meta-analysis
Source: Int J Behav Nutr Phys Act. 2020 Mar 4;17:31. doi: 10.1186/s12966-020-00938-3 (PMC7055033; doi:10.1186/s12966-020-00938-3)
Supplement: Supplementary file 16 — Additional file 16: Supplemental table 3. Validity characteristics. [file 12966_2020_938_MOESM16_ESM.pdf]

Supplemental table 3. Validity characteristics

First author, year	Population	Self-report measure				Device measure				Correlation	MD: Self-report – device*
		Name	Domain or type	Units	Mean (SD)*	Name	Domain or type	Units	Mean (SD)*		
Abraham, 2009	Apparently healthy	Bouchard Activity Record	Sitting (categories 1 and 2)	min/d	487.0 (194.3)	ActiGraph GT1M	ST	min/day	650.6 (111.8)	r = 0.56, p <.001	163.6 (150.6)
						activPAL	Sitting/lying		518.5 (147.8)	r = 0.87, p <.001	31.5 (107.6)
Adams, 2015	Apparently healthy	IPAQ	Sitting	min/week	Seattle: 2555.0 (1230.5), Baltimore: 2553.7 (1253.4)	ActiGraph	ST	min/day	Seattle: 494.4 (106.4), Baltimore: 506.8 (105.2)	NR	NR
Aguilar-Farias, 2015	Community-dwelling older adults	Generic sitting question	Sitting	h/day	Average = 6.16 (2.35); Weekday = 6.19 (2.41), Weekend day = 6.09 (2.35)	activPAL	ST	h/day	Average = 9.60 (1.66), weekday = 9.55 (1.64), weekend day = 9.68 (1.96)	Average = 0.33, weekday = 0.31, weekend = 0.28	Average = -3.53 (LoA: -8.10, 1.05), weekend = -3.45 (LoA: -8.57, 1.25), yesterday = -3.30 (LoA: -8.78, 2.19)
		MARCA	ST		Average = 11.42 (1.99)					Yesterday = 0.49, average = 0.63, day before yesterday = 0.67	Average = 1.66 (LoA: -1.49, 4.82) day before yesterday = 0.82 (LoA: -3.54, 5.18), yesterday = 1.47 (LoA: -3.45, 6.40)
Aguilar-Farias, 2017	General population	GPAQ	Sitting	min/day	Total: 259.7 (211.67), men: 241.9 (164.53), women: 272.4 (239.67)	ActiGraph GT3X	ST	min/day	Total: 555.2 (168.03), men: 533.7 (165.17), women: 571.0 (170.35)	r = 0.23, p<.001, men: r = 0.23, p = 0.03, women: r = 0.21, p = 0.02	-293.9 (LoA: -768.9, 181.2)
Aittasalo, 2017	Employees from small to medium workplaces	WSQ	Sitting (work, home, travel)	min/day	509 (142)	Hookie AM13	ST	min/day	298.5 (81.3)	NR	NR
Alkahtani, 2016	Male university students	GPAQ	Sitting	h/day	Median (IQR): 6.0 (3.0, 9.0)	ActiGraph GT3X	ST	h/day	Median (IQR): 9.4 (8.6, 10.2)	0.08	3.3 (LoA: -3.1, 9.7)
Anjana, 2015	Urban and rural populations	MPAQ	TV, sitting	min/week	4965 (869)	ActiGraph GT3X	ST	min/week	5009 (785)	r = 0.484 (95% CI: 0.32, 0.62)	44.4 (LoA: -1599.1, 1687.8)
Barone Gibbs, 2017	Older adults	CHAMPS	TV, computer (work or recreation), riding in car	h/day	Median (IQR): Sit less: 7.8 (7.1, 10.1), Get Active: 10.3 (6.3, 13.5)	SenseWear Pro	ST	h/day	Sit less: 10.5 (0.3), Get Active: 10.3 (0.6) *least	NR	NR

First author, year	Population	Self-report measure				Device measure				Correlation	MD: Self-report – device*
		Name	Domain or type	Units	Mean (SD)*	Name	Domain or type	Units	Mean (SD)*		
									square means and SE		
Barwais, 2014	Sedentary adults	Sedentary and Light Intensity Physical Activity Log (7-Day SLIPA log)	Sum of SBs	h/day	Sedentary: total = 15.2 (1.7); men = 15.7 (1.7); women = 14.4 (1.5). Sitting: total = 9.3 (2.0); men = 9.8 (1.7); women = 8.2 (2.3)	ActiGraph GT3X	ST	h/day	Sedentary: total = 15.5 (1.9); men = 16.0 (1.9); women = 14.6 (1.5). Sitting: total = 7.5 (1.1); men = 7.7 (1.0); women = 7.2 (1.2)	Pearson: sedentary = 0.86 (95% CI: 0.70, 0.94), sitting = 0.60 (95% CI: 0.24, 0.81), Weighted kappa: sedentary = 0.61 (95% CI: 0.443, 0.769); sitting = 0.22 (95% CI: 0.059, 0.389)	Sedentary = -0.3 (LoA: -2.1, 1.6), sitting = 1.8 (LoA: -1.5, 5.0)
Benitez-Porres, 2013	Fibromyalgia patients	IPAQ	Sitting	min/day	222 (116)	ActiGraph GT1M	ST	min/day	598 (126)	r = 0.148, p = 0.164	-371.3 (LoA: -76.5, -666.1)
Biddle, 2012	Young adults at risk for diabetes	IPAQ	Sitting	min/day	NR	ActiGraph GT3X	ST	min/day	615.07 (103.94)	r = 0.326, p<0.01	-251 (LoA: 499, -1001)
		Marshall Questionnaire	ST							r = 0.311, p<0.01	22 (LoA: -518, 560)
Biswas, 2018	Cardiac rehab patients	SBQ	SBs	h/day	Weekday: 7.8 (3.4), weekend day: 8.1 (4.0)	ActiGraph GT3X	ST	h/day	4.6 (2.7)	R = 0.21, p = 0.26	NR
Blanchard, 2010	Older adults	Diary	Driving	min + h/day	4h24min (3h45min)	CarChip E/X	Driving time	min + hrs/day	4h11min (3h38min)	NR	NR
Blikman, 2014	Patients after total hip Arthroplasty	IPAQ-SF, IPAQ-LF	Sitting	NR	NR	NR	ST	NR	NR	r = 0.18 (long form), r = 0.01 (short form)	NR
Bond, 2013	Bariatric surgery patients	SBQ	SBs	h/day	Weekdays: 9.98 (5.28), weekend day: 8.71 (4.62), all days: 9.61 (4.83)	SenseWear Pro2	ST	h/day	Weekdays: 9.46 (2.06), weekend: 8.78 (1.98), all days: 9.27 (1.85)	Weekdays ICC = 0.22, p = 0.06, weekend: ICC = 0.19, p = 0.08, all days ICC = 0.22, p = 0.06	Weekdays = -0.51 (LoA: 0.2, 13.6), weekend days = 0.08 (LoA: 0.1, 11.5), all days = -0.34 (LoA: 0.2, 13.1)
Bonn, 2015	Men who underwent Prostate Specific Antigen testing	Active-Q	SBs	min/day	606 (136)	GENEA	ST	min/day	789 (186)	r = 0.19 (95% CI: 0.04-0.34)	-178 (LoA: -606, 250)

First author, year	Population	Self-report measure				Device measure				Correlation	MD: Self-report – device*
		Name	Domain or type	Units	Mean (SD)*	Name	Domain or type	Units	Mean (SD)*		
Boyle, 2015	Colon cancer survivors	Marshall Domain-Specific Sitting Questionnaire	SBs	h/day	8.5 (95%CI: 8.0-9.0)	ActiGraph GT3X	ST	h/day	8.7 (95% CI: 8.5-9.0)	r = 0.19, ICC = 0.16 (95% CI: 0.01, 0.30)	NR
Bruening, 2016	College students	devilSPARC mEMA	SBs	% of reports	81.40%	ActiGraph GT3X	ST	% of wear time	53.60%	NR	NR
Bueno-Antequera, 2017	Outpatients with severe mental illness	SBQ	SBs	h/day	9.2 (3.6)	SenseWear	ST	h/day	8.9 (2.4)	Weekday: r = 0.21, p = 0.044; weekend: r = 0.16, p = 0.132, average r = 0.20, p = 0.053)	LoA: weekdays (8.37, -6.82), weekend days (9.44, -8.53) and average day ST (8.23, -6.89)
Busschaert, 2015	General population	last-7-day SB questionnaire (SIT-Q-7d)	Sitting/lying down	mins/day	Adults: weekday = 648.18 (270.12), weekend day = 512.27 (238.02), average = 609.35 (242.73). Older adults: weekday = 515.45 (176.26), weekend day = 506.36 (160.32), average = 512.86 (162.08)	activPAL	Sitting	mins/day	Adults: weekday = 479.18 (156.26), weekend day = 447.76 (99.07), average = 470.20 (125.46). Older adults: weekday = 477.67 (111.33), weekend day = 551.76 (115.60), average = 498.84 (102.47)	Adults: weekday r = 0.52 (95% CI: 0.22; 0.73); weekend day r = 0.06 (−0.29; 0.40); average r = 0.49 (0.18; 0.71). Older adults: weekday r = 0.50 (95% CI: 0.19; 0.72); weekend day r = 0.38 (0.04; 0.64); average r = 0.48 (0.16; 0.71)	Older adults: weekday = 35.80 (LoA: 535.51, 11.24); weekend day = -45.46 (-577.29, -0.37); average = 16.32 (-534.18, -40.21)
Camhi, 2015	Metabolically healthy obese (MHO) and metabolically abnormal obese (MAO), young black and white women	NR	Sitting	mins/day	MHO = 299.0 (56.4), MAO = 422.4 (78.6)	NR	ST	h/day	MHO = 585.8 (21.6), MAO = 643.8 (30.1)	NR	NR
Celis-Morales, 2012	Chilean population (general pop)	IPAQ	Sitting	min/day	454.2 (103.1)	Actitrainer	ST	min/day	523.1 (90.9)	r = 0.654, 95% CI: 0.570, 0.738; concordance correlation = 0.519, 95% CI: 0.402, 0.697	-68.8 (LoA: -228.3, 90.8),

First author, year	Population	Self-report measure				Device measure				Correlation	MD: Self-report – device*
		Name	Domain or type	Units	Mean (SD)*	Name	Domain or type	Units	Mean (SD)*		
Cerin, 2012	Urban older adults	IPAQ - Long	Sitting	min/week	1968 (621)	ActiGraph GT1M	ST	min/week	3585 (792)	0.16, NS	-1646 (95% CI: -1854, -1439)
Cerin, 2016	General population	IPAQ - Long	Sitting	h/day	6.3 (2.9)	ActiGraph GT1M and GT3X	ST	h/day	8.8 (1.7)	r = 0.37 (95% CI: 0.35, 0.40)	NR
Chapman, 2016	Non-institutionalised adults with mental illness	Marshall Domain-Specific Sitting Questionnaire	Sitting: travel; work; TV; computer; leisure time (not including TV)	h/day	Median (IQR) = 10.3 (6.3, 14.5), mean = 10.6 (4.7)	ActiGraph GT3X	ST	h/day	Median (IQR): 9.2 (7.9, 10.6), mean = 9.1 (1.9)	r (69) = 0.21, p = 0.08	NR
Chasan-Taber, 2004	Pregnant women	Pregnancy Physical Activity Questionnaire (PPAQ)	SBs (household/ care giving, occup., sports/exercise, travel, inactivity)	NR	NR	ActiGraph	ST	NR	NR	Swartz cut-point r = -0.17, Hendelmen: r = -0.34, Freedson: r = 0.12	NR
Chastin, 2018	Seniors	-Two single item direct measures of ST<br>-Direct question about the total time spent sitting;<br>-Visual analogue scale of proportion of day spent sitting;<br>-Single item proxy measure (TV time)<br>-Pattern based on report of # of bouts of sitting and average duration (total duration);<br>-Sum of time spent in 13 SBs (TV, work, computer/ screen for leisure, reading, listening or playing music, seated hobbies, talking, eating, self-care, activities of daily	Sitting, TV, multi-SBs, domain-specific SB	min/day	Previous day recall: single item total ST = 422.5 (160.9), single item proportion = 49.8% (17.5%), single TV = 191.5 (123.2), sum of domains = 499.0 (196.8), sum of behaviours = 811.9 (331.5), pattern = 589.8 (587.3). Previous week recall: single item = 486.0 (486.5), single proportion = 50.9% (16.2%), TV = 235.9 (206.5), sum = 662.6 (466.5), sum of behaviours = 1125.2 (867.8), pattern = 845.4 (1796.7). Usual	activPAL	Sitting	min/day	628.7 (120.7)	Previous day: single item r = 0.20, p<.001; single item proportion r = 0.28, p<.001; single TV r = 0.24, p<.001; sum of domains r = 0.23, p<.001; sum of behaviours r = 0.23, p<.001; pattern r = 0.17, p<.001. Previous week recall: single item r = 0.23, p<.001; single item proportion r = 0.36, p<.001; TV r = 0.23, p<.001; sum of domains r = 0.30, p<.001; sum of behaviours r = 0.32, p<.001; pattern r = 0.23, p<.001. Unanchored/usual day: single item r = 0.20, p<.001; single item proportion r = 0.32, p<.001; TV r = 0.26,	Previous day recall: single item LoA = -146.8, 560.8; single item proportion LoA = -22.1, 47.8; TV LoA = 144.8, 733.2; sum of domains LoA = -272.5, 533.1; sum of behaviours LoA = -651.2, 366.7; pattern LoA = -471.6, 747.5. Previous week recall: single item LoA = -149.9, 563.9; single item proportion LoA = -19.3, 42.9; TV LoA = 80.6, 731.9; sum of domains LoA = -412.9, 482.1; sum of behaviours LoA = -755.2, 265.3; pattern LoA = -528.8, 726.9. Usual day: single item LoA = -135.1, 549.2; single item proportion LoA = -18.9, 51.1; TV

First author, year	Population	Self-report measure				Device measure				Correlation	MD: Self-report – device*
		Name	Domain or type	Units	Mean (SD)*	Name	Domain or type	Units	Mean (SD)*		
		living, napping, sitting in transport and sitting during leisure activities outside the home (e.g. watching a play at the theatre)) -Sum of domains time spent at work, home, transport and leisure			day: single item = 379.2 (152.3), single proportion = 46.6% (18.1%), TV = 215.8 (123.6), sum of domains = 557.4 (234.0), sum of behaviours = 933.8 (426.8), pattern = 266.7 (743.9)					p<.001; sum of domains r = 0.16, p<.001; sum of behaviours r = 0.33, p<.001; pattern r = 0.02, p = 0.624.	LoA = 124.7, 705.4; sum of domains LoA = -372.8, 528.8; sum of behaviours LoA = -725.3, 285.6; pattern LoA = -33.8, 979.6.
Chastin, 2014	General population	IPAQ-LF	Sitting (work/home/leisure), sitting during travel	h/day	Total week = 6.6 (2.6); total week + transportation = 8.1 (2.6); total weekdays = 7.4 (2.7); total weekend days = 5.4 (2.6)	activPAL	Sitting	h/day	Total week = 9.9 (1.9); total week + transportation = 9.9 (1.9); total weekdays = 10.6 (1.6); total weekend days = 10.9 (1.7)	Total week ICC = 0.112, r = 0.114, p = 0.351; total week + transportation ICC=0.149, r = 0.159, p = 0.193; total weekdays ICC = 0.159, r = 0.174, p= 0.154; total weekend days ICC = 0.275, r = 0.010, p = 0.936	Total week = 3.40 (95% CI: -2.15, 8.93); total week + transportation = 2.15 (955% CI: -3.71, 7.22); total weekdays = 3.43 (95% CI: -2.08, 8.94); total weekends = 4.64 (95% CI: -2.01, 11.30)
Chau, 2012	Workers	OSPAQ	Occup. sitting	min/day	median = 406 (IQR: 308-450)	ActiGraph GT1M	ST	min/day	NR	r = 0.65, p<0.01	22 (95% CI: 3, 41, LoA: -141.63, 185.18)
Chau, 2011	Workers	Workforce Sitting Questionnaire	Occup. sitting, TV, computer at home, leisure activities, travel on workdays and non-workdays	min/day	Median (IQR): All: At work = 360 (311, 394); total all domains, workday = 600 (562, 648); total all domains, non-work days = 486 (417, 550); average total, work and non-workdays = 567 (518, 606). Women: At work = 366 (337, 408); total all domains, workday = 606 (563, 648); total all domains, non-work days = 465 (418, 538); average total,	ActiGraph GT1M	ST	min/day	NR	All: At work r = 0.45, p<.01; total all domains, workday r = 0.34, p<.01; total all domains, non-work days r = 0.23, p<.05; average total, work and non-workdays r = 0.40, p<.01. Women: At work median r = 0.38, p<.01; total all domains, workday r = 0.31, p<.05; total all domains, non-work days r = 0.22, NS; average total, work and non-workdays r = 0.46, p<.01. Men: At work r = 0.41, p<.05; total all domains, workday r = 0.29, NS; total all domains, non-	Average total = 44.55 (LoA: -295.31, 384.41), sitting at work = 1.58 (LoA: -227.86, 231.02)

First author, year	Population	Self-report measure				Device measure				Correlation	MD: Self-report – device*
		Name	Domain or type	Units	Mean (SD)*	Name	Domain or type	Units	Mean (SD)*		
					work and non-workdays = 571 (532, 607). Men: At work = 344 (275, 378); total all domains, workday = 583 (562, 654); total all domains, non-work days = 507 (392, 558); average total, work and non-workdays = 566 (484, 603)					work days r = 0.18, NS; average total, work and non-workdays r = 0.26, NS.	
		IPAQ	Sitting	min/day	Median (IQR): All: weekday = 598 (542, 643); weekend day = 493 (421, 550); average total = 565 (511, 606). Women: weekday = 598 (553, 639); weekend day = 471 (421, 541); average total = 561 (512, 604). Men: weekday = 598 (536, 661); weekend day = 496 (430, 562); average total = 570 (493, 607)					All: weekday r = 47, p<.01; weekend day r = 0.31, p<.01; average total r = 0.46, p<.01. Women: weekday r = 0.43, p<.01; weekend day r = 0.28, NS; average total r = 0.45, p<.01. Men: weekday r = 0.57, p<.01; weekend day r = 0.37, NS; average total r = 0.43, p<.05.	
Chau, 2014	Workers	Workforce Sitting Questionnaire (WSQ)	Occup. sitting, TV, computer at home, leisure activities, travel on workdays and non-workdays	min/day	Sitting at work on workday = 426 (121)	activPAL3	Sitting at work	min/day	347 (59)	NR	NR
		OSPAQ	Occup. sitting		Sitting at work = 403 (114);						

First author, year	Population	Self-report measure				Device measure				Correlation	MD: Self-report – device*
		Name	Domain or type	Units	Mean (SD)*	Name	Domain or type	Units	Mean (SD)*		
Chinapaw, 2009	Workers	Activity Questionnaire for Adults and Adolescents (AQuAA)	SBs	min/week	Median (IQR) = 3045 (2455, 3610)	ActiGraph 7164	ST	min/week	Median (IQR) = 5307 (4956, 5458)	r = 0.15, NS	Median diff = 2216 (IQR: 1579, 2729)
Chu, 2018	University desk workers	ASBQ	Occup.; travel; leisure-time; other	min/day	164.1 (148.9)	ActiGraph GT3X BT	ST	min/day	663.8 (70.4)	Sum r = 0.10 (95% CI: -0.12, 0.32); occup. r = 0.18 (95% CI: -0.05, 0.38); transportation r = 0.11 (95% CI: -0.12, 0.32); eating r = 0.07 (95% CI: -0.16, 0.29); TV r = 0.05 (95% CI: -0.18, 0.26); leisure-time computer use r = -0.10 (95% CI: -0.32, 0.12); other leisure activities r = -0.05 (95% CI: -0.27, 0.18)	4.6 (LoA: -431.2, 440.4)
		GPAQ	Sitting		213.2 (150.9)					r = 0.28 (95% CI: 0.11, 0.47).	-174.8 (LoA: -556.1, 206.5)
Clark, 2015	General population	AusDiab3 questionnaire	Work, travel, TV viewing, leisure computer use and "other" purposes, on weekdays and weekend days	h/day	6.85 (2.69), women = 6.43 (2.47); men = 7.36 (2.86)	activPAL3	Sitting	h/day	Total = 8.86 (1.81), women = 8.55 (1.81); men = 9.24 (1.74)	Total r = 0.46 (95% CI: 0.40, 0.52); women r = 0.47 (95% CI: 0.39, 0.55); men r = 0.42 (95% CI: 0.32, 0.41)	Total = 2.01 (2.45); women = 2.12 (2.26); men = 1.87 (2.66)
Clark, 2016	University population (students and staff)	Past-day Adults' ST-University (PAST-U)	Sitting or lying down: work, study, travel, TV viewing, leisure-time computer, reading, eating, socialising and other purposes	h/day	Composite sum = 10.72 (2.42)	activPAL	Sitting	h/day	10.65 (2.42)	ICC = 0.64 (95% CI: 0.45, 0.77)	5 min (LoA: -3.9, 4.1)

First author, year	Population	Self-report measure				Device measure				Correlation	MD: Self-report – device*
		Name	Domain or type	Units	Mean (SD)*	Name	Domain or type	Units	Mean (SD)*		
Clark, 2011	Full time workers	NR	Occup. sitting, breaks in sitting	h/day	7.05 (1.31)	ActiGraph GT1M	ST	h/day	6.60 (0.94)	r = 0.39 (95% CI = 0.22–0.53)	0.45 (95% CI: 0.23, 0.66)
Clark, 2013	Breast cancer survivors	Past-day Adults' Sedentary Time (PAST)	Single weekday: sitting or lying while at work, travelling, watching TV, using the computer (excluding work), reading (excluding work), hobbies, and any other not reported in previous	h/day	Composite sum = 8.20 (2.81)	activPAL3, ActiGraph GT3X	Sitting/lying, ST	h/day	ActivPAL: On day of recall total time = 9.2 (2.4); average daily = 8.8 (1.5), ActiGraph: day of recall = 8.9 (1.4); average day = 8.9 (1.0)	ActivPAL: Day of recall r = 0.58 (95% CI: 0.40, 0.72); average daily r = 0.34 (95% CI: 0.12, 0.43), ActiGraph: day of recall r = 0.51 (95% CI: 0.29, 0.68); average daily r = 0.45 (95% CI: 0.22, 0.63)	activPAL of 5+ mins: = -0.15 (95% CI: -0.72, 0.42, LoA: -4.90, 4.60)
Clayton, 2016	Men with prostate cancer and history of Androgen-Deprivation Therapy use	IPAQ	Sitting	h/day	5.9 (3.1)	activPAL3	Sitting	h/day	9.6 (2.5)	r = 0.22, p = 0.04	3.5
Cleland, 2014	Middle-aged men and women	GPAQ	Sitting	min/day	Median (IQR): total = 300 (180, 435); women = 240 (180, 360); men = 360 (180, 480)	ActiGraph GT3X	ST	min/day	Median (IQR): total = 696 (617, 751); women = 660 (613, 719); men = 715 (627, 776)	Overall: r = 0.187, p = 0.135; women r = 0.378, p = 0.027; men r = -0.053, p = 0.778	-348.7 (LoA: -721.1, 23.7)
Cleland, 2018	Older adults	IPAQ - Long	Sitting	min/day	Median (IQR): weekday: total median = 300.0 (197.5, 420.0); men = 300.0 (240, 435); women = 240.0 (180, 360). Weekend day: total median = 300 (240, 420);	ActiGraph GT3X	ST	min/day	Median (IQR): weekday: total median = 486.9 (425.8, 566.5); men = 513.5 (460.5, 600.8); women = 453.2 (393.0, 522.5). Weekend day: total = 501.4	Weekday: total r = 0.70, p = 0.01; men r = 0.49, p = 0.01; women r = 0.47, p = 0.01. Weekend day: total r= 0.26, NS; men r = 0.55, p = 0.01; women r = 0.36, p = 0.01	Weekday = -168.6 (SD = 144.5, LoA: -451.8, 114.6), weekend day = -173.9 (SD = 136.6, LoA: -441.6, 93.8)

First author, year	Population	Self-report measure				Device measure				Correlation	MD: Self-report – device*
		Name	Domain or type	Units	Mean (SD)*	Name	Domain or type	Units	Mean (SD)*		
					men = 360 (240, 480); women = 240 (180, 360)				(436.2, 580.2); men = 529.3 (459.3, 597.2); women = 477.1 (419.9, 549.7)		
Clemes, 2012	University staff	NR	Sitting	min/day	Weekday: 466.8 (161.7); weekend day: 391.4 (171.3)	ActiGraph GT1M	ST	min/day	Weekday: 639.7 (133.9); weekend day: 612.4 (132.7)	Weekday: r = 0.70 (p<.001); ICC = 0.82 (p<.001); weekend day: r = 0.55 (p< .001); ICC = 0.69 (p< .001)	Weekday = -173 (LoA: -401.6, 55.6); weekend = -218.6 (LoA: -509.6, 72.4)
		NR	Travel, work, TV, computer at home, leisure time, not including TV		Weekday: 626.0 (222.1); weekend day: 615.6 (280.0)					Weekday r = 0.54 (p<.001); ICC = 0.64 (p<.001); weekend day r = 0.13 (p = 0.41); ICC = 0.20 (p = 0.23)	Weekday = -13.7 (LoA: -382.0, 354.6); weekend day = -4.2 (LoA: -578.5, 570.2)
Conroy, 2013	College students	IPAQ - short	Sitting	min/day	369.68 (154.62)	ActiGraph GT3X	ST	Proportion	66.54% (8.63%)	r = 0.34	NR
Craig, 2003	General population	IPAQ-SF and IPAQ-LF	Sitting	min/day	NR	CSA 7164	ST	NR	NR	Long form range r = 0.14 to 0.51 (depended on mode), short form range: 0.07 to 0.61	NR
Curry, 2015	South Asian women	IPAQ - short	Sitting	min/week	2207.17 (1868.86)	ActiGraph GT1M and GT3X	ST	min/week	3474.94 (508.06)	r = -0.140, p = 0.229	223.83 (LoA: -337.24, 785.66)
Dahl-Petersen, 2013	Inuit	Modified IPAQ - long	Sitting	h/day	Median (IQR): men = 11.9 (10.0, 14.0), women = 11.3 (10.0, 13.2)	Actiheart	ST	h/day	Median (IQR): men = 14.9 (13.0, 17.2), women = 15.8 (13.8-17.5)	Men r = -0.01, p = 0.8, omen r = 0.06, p = 0.10	NR

First author, year	Population	Self-report measure				Device measure				Correlation	MD: Self-report – device*
		Name	Domain or type	Units	Mean (SD)*	Name	Domain or type	Units	Mean (SD)*		
De Cocker, 2016	Flemish employees	WBQ	Sitting on workday and non-workday while 1) travel, 2) work, 3) TV, 4) computer at home (non-work related), and 5) doing other leisure activities	min/day	Total Workday sitting: tailored = 507 (104), generic = 457 (107), control = 449 (126), Total non-workday sitting tailored = 141 (70), generic = 130 (63), control = 123 (58), Sitting at work: tailored = 338 (107), generic = 288 (59), control = 281 (65), Sitting during transport: tailored = 78 (84), generic = 66 (79), control = 81 (106), TV viewing: tailored = 100 (57), generic = 95 (62), control = 91 (68), personal computer use: tailored = 50 (46), generic = 51 (40), control = 58 (62), other leisure sitting: tailored = 101 (42), generic = 99 (61), control = 95 (48)	activPAL	Sitting	h/day	Total sitting awake: tailored = 576 (109), generic = 578 (101), control = 605 (96)	NR	NR
De Greef, 2011	Patients with Type 2 diabetes	IPAQ	Sitting	min/day	Intervention: 463 (122), control: 505 (174)	ActiGraph 7164	ST	min/day	Intervention: 1140 (90), control: 1118 (109)	NR	NR
Doyle, 2018	Emirati university students	GPAQ (Arabic version)	Sitting	min/day	Median = 360 (IQR: 240-600)	ActiGraph GT3X	ST	min/day	Median = 492 (IQR: 431-563)	r = -0.02, 95% CI: -0.35, 0.33	99 (LoA: -539, 341)

First author, year	Population	Self-report measure				Device measure				Correlation	MD: Self-report – device*
		Name	Domain or type	Units	Mean (SD)*	Name	Domain or type	Units	Mean (SD)*		
Duncan, 2019	Individuals with schizophrenia	IPAQ-SF	Sitting	min/day	424.6 (247.0)	ActiGraph GT3X	ST	min/day	1 minute bouts: 515.7 (135.8); 10 min bouts: 403.1 (138.1); 30 min bouts: 213.6 (111.9)	1 minute bouts: ICC: 0.21 (CI: 0.04–0.36), p = 0.004, r = 0.25, p = 0.029; 10 minute bouts: ICC = 0.23 (CI: 0.06–0.39), p = 0.004, r = 0.30, p = 0.009; 30 minute bouts: ICC = 0.09 (CI: −0.05–0.24), p = 0.04, r = 0.26, p = 0.023	1 minute bouts: = -86.1 (95% LoA: -569.9, 397.6), 10 minute bouts: = 26.8 min (95% LoA: -458.7, 512.3); 30 minute bouts: = 220.7 (LoA: -266.6, 708.0)
Dyrstad, 2014	General population	IPAQ-SF	Sitting	min/day	420 (95% CI: 412–429)	ActiGraph GT1M	ST	min/day	551 (95% CI: 547, 555)	NR	All = -131 (SD = 166), men = -127 (SE = 6); women = -134 (SE = 5)
Ekblom, 2015	General population	NR	Sitting	h/day	Men: median (IQR) = 300 (180, 420); women = 240 (180, 420)	ActiGraph GT3X	ST	min/day	Men: median (IQR) = 528 (470, 589); women median = 502 (438, 560)	r = 0.30, p<0.05	Median difference = -185 (5th, 95th percentile: -467, 119)
Ekelund, 2006	Workers	IPAQ-SF	Sitting	h/day	men: median (IQR) 6.9 (4.6, 8.6); women: 6.9 (5.2, 8.9)	ActiGraph 7164	ST	min/day	Men: 427 (74), women: 394 (69)	r = 0.16, p<0.05	NR
Ellingson, 2012	Healthy women	IPAQ-LF	Sitting (weekday + weekend)	min/week	Meets: 3125 (883); inactive: 3357 (1128)	ActiGraph GT1M	ST	min/day	Meets: 580 (80); inactive: 611 (47)	NR	NR
Elramli, 2017	People within first 5 years of being diagnosed with rheumatoid arthritis	IPAQ-LF	Sitting (weekday + weekend)	h/day	Weekday: intervention = 5.3 (1.9); control = 4.7 (2.5). Weekend day: intervention = 5.1 (2.1); control = 4.6 (2.5)	activPAL	Sitting/lying	h/day	Intervention: 18.0 (1.9); control: 18.5 (1.4)	NR	NR
Emadian, 2017	Overweight & obese South Asian men	IPAQ-LF	Sitting (weekday + weekend)	min/day	577.8 (860.7)	ActiGraph GT3X	ST	min/day	551.4 (95.0)	NR	-56.2 (p = 0.610, LoA: 1631.2, 1518.9)
English, 2016	Individuals who had	MARCA	SBs (TV, video games,		Total Sitting: stroke = 729	activPAL3	Sitting	h/day	Stroke: 10.9 (2.0), control: 8.2 (2.0)	NR	NR

First author, year	Population	Self-report measure				Device measure				Correlation	MD: Self-report – device*
		**Name**	**Domain or type**	**Units**	**Mean (SD)***	**Name**	**Domain or type**	**Units**	**Mean (SD)***		
	experienced a stroke		reading, computer)		(143), control = 602 (153)						
Fitzsimons, 2012	Scottish adults	IPAQ-LF (weekday only asked)	Sitting (weekday)	min/day	504 (192)	activPAL3	Sitting	min/day	667 (SD 81)	ICC = 0.24 (95% CI -0.07, 0.51)	162 (95% CI: 105, 220)
Fitzsimons, 2013	Older Scottish adults	SBQ	Total ST (combined items)	h/week	51.8 (99.5), weekdays = 33.8 (75.0), weekend days = 18.0 (31.5)	activPAL3	Sitting/lying	h/day	18.06 (1.54)	NR	NR
Fjeldsoe, 2009	Women with young children	AWAS	Sitting (across multiple domains)	min/week	Median (IQR): 2385 (1480)	ActiGraph MT1	ST	min/week	Median (IQR): 2829 (568)	r = 0.32, p = 0.006	NR
Fowles, 2017	University population	CSEP PASBQ	Occup., leisure & total sitting	h/day	6.4 (3.5)	ActiGraph GT3X	ST	h/day	12.2 (1.2)	r = 0.29, p = 0.13	NR
French, 2007	Transportation workers	modified IPAQ	Sitting	h/day	9.3 (SE = 0.13)	ActiGraph	ST	min/day	342.8 (SE = 6.2)	NR	NR
Gao, 2017	Finnish and Chinese office workers	Long-term and short-term ST	Occup. sitting	% of day	Long-term: 79.0 (13.5); short-term: 79.3 (14.3)%	X6-1a	ST	% of day	76.6 (12.4)%	Long-term: (r = 0.532, 95% CI: 0.336, 0.684, p<.001), short-term: r = 0.533 (95%CI: 0.449, 0.607, p<0.001)	Long term = 2.4% (95% CI: -0.5, 5.3, p = 0.091, LoA: -21.2, 25.9), short-term = 2.2% (95% CI: 0.7, 3.6, p = 0.005, LoA: -24.2, 28.5)
Gardiner, 2011	Older adults	modified Salmon questionnaire	Watching TV, computer, reading, socialising, travel and hobbies, and a summary measure (total ST time)	h/day	Total ST: 7.3 (1.8)	ActiGraph GT1M	ST	h/day	Median: 10.2 (IQR: 9.5, 10.9)	r = 0.30 (95% CI: 0.02, 0.54)	-3.6
Gennuso, 2015	Older adults	Yale Physical Activity Survey for Older Adults (YPAS)	SBs	h/day	NR	ActiGraph GT1M	ST	h/day	9.2 (1.3)	8.6% agreement between the YPAS and accelerometer-derived SB time	NR

First author, year	Population	Self-report measure				Device measure				Correlation	MD: Self-report – device*
		Name	Domain or type	Units	Mean (SD)*	Name	Domain or type	Units	Mean (SD)*		
		CHAMPS								r = 0.14, p = 0.28;	
Gennuso, 2016	Older adults	Modified Salmon questionnaire (Gardiner)	Watching TV, using computer, reading, socializing, passive travel, doing hobbies, other activities	h/day	9.0 (3.3)	activPAL	Sitting/lying	h/day	Median: 9.4 (IQR: 8.2, 10.5)	r = 0.06, p = 0.72, Lin's concordance coefficient value was 0.07 (95 % CI -0.18, 0.30)	0.31 (95 % CI: -6.74, 7.37)
Gibbs, 2015	Overweight & obese young adults	EARLY Trials Sedentary Behavior Questionnaire	SBs: TV, computer or video games, office work, reading or other recreation, using a phone, and travel-work, computer, paperwork	h/day	Median (IQR): 10.0 (8.1-12.9)	SenseWear armband	ST	h/day	Median (IQR): 9.1 (7.8-10.2)	r = 0.21, p<.001	1.1 (95% LoA: -4.9, 11.9)
		GPAQ	Sitting		Median (IQR): 8.0 (5.0, 10.0)					r = 0.32, p<.001	0.7 (LoA: -7.8, 5.8)
Gilbert, 2016	Patients with Rheumatoid Arthritis	modified Yale Physical Activity Survey (YPAS)	SBs	h/day	13.00 (2.59)	ActiGraph GT1M	ST	h/day	9.86 (1.38)	Pearson r = 0.29 (95% CI: 0.15, 0.42, p<.001)	LoA demonstrated a MD of ± 4.0 h/day
Golubic R, 2014	Middle-aged men and women	Recent Physical Activity Questionnaire (RPAQ)	SBs (leisure time, occup., travel, and domestic life)	h/day	Total: 12.7 (3.0), women: 12.9 (2.7), men: 13.4 (3.1)	Actiheart	ST	h/day	Total: 15.8 (2.3), women: 15.9 (2.2), men: 15.6 (2.6)	Total: r = 0.20 (95% CI: 0.14, 0.25)	Total = -3.1 (LoA: -9.6, 4.9), women = -3.0 (LoA: -9.0, 4.1), men = -2.2 (LoA: -8.3, 5.5)
Gomersall, 2015	Adults from an Australian university	MARCA	SBs	h/day	11.00 (2.33)	activPAL	Sitting/lying	h/day	10.4 (2.6)	r = 0.77 (95% CI: 0.64, 0.86; p<.001)	Mean bias = 0.59 (LoA: -2.35, 3.53)
Gordon, 2013	Adults with sedentary occupations	IPAQ	Total day sitting	h/day	All-day sitting: 548.7 (167.2)	ActiGraph GT3X & activPAL	ST	min/day	ActiGraph: 364.4 (37.3), activPAL at work: 381.6 (52.0)	NR	NR
		SBQ	SBs		Sitting at work: 360.0 (171.7)		Sitting/lying at work				

First author, year	Population	Self-report measure				Device measure				Correlation	MD: Self-report – device*
		Name	Domain or type	Units	Mean (SD)*	Name	Domain or type	Units	Mean (SD)*		
Grimm, 2012	Older adults	IPAQ-SF	Sitting	min/day	Total: 336.4 (184.1); men: 330.7 (218.5), women: 338.4 (171.4)	ActiGraph 7164	ST	min/day	Total: 597.2 (96.8), men: 630.6 (94.1), women: 586.4 (95.7)	All: r = 0.042, NS, men: r = 0.051, NS, women: r = 0.050, NS	Total = 262.0 (LoA: 608.2, -84.2), men = 294.3 (LoA: 664.9, -76.3), women = 250.4 (LoA: 568.8, -86.0)
Gupta, 2017	Blue collar workers	None	Workday sitting	min/day	271.8 (160.5)	ActiGraph GT3X	Workday sitting	min/day	475.8 (138.3)	r = 0.32, p<0.001	204.1 (LoA: -139.7, 547.8)
Haakstad, 2010	Pregnant women	physical activity and pregnancy questionnaire (PAPQ)	Daily sitting/lying	min/day	1122.2 (111.7)	ActiReg	Sitting/lying	min/day	1117.2 (144.5)	r = 0.288, p = 0.013	3.0 (LoA: -309.6, 315.5)
Hagstromer, 2010	General population	IPAQ-LF	Sitting (weekday + weekend)	min/day	All: 590 (208), men: 632 (195), women: 557 (211)	ActiGraph 7164	ST	min/day	All: 466 (87), men: 477 (94), women: 457 (80)	All r = 0.23, p<0.01, men: r = 0.13, p<0.05, women: r = 0.26, p<0.01	Sitting MD (SD) = 130 (207), p<.001. Differences (i.e., error) between the IPAQ and ActiGraph scores increased as the min/day in sitting reported in the IPAQ increased (R^2 = 0.50)
Hansen, 2014	General population	IPAQ-LF	Sitting (weekday + weekend)	h/day	Median (IQR); women: 13.8 (12.0, 16.3); men: 14.4 (12.6, 18.0)	ActiHeart	ST	h/day	Median (IQR): women: 15.6 (14.4, 17.1); men: 16.3 (14.6, 17.5)	NR	NR
Headley, 2018	College employees	OSPAQ	Occup. sitting	min/day & % of day	327.7 (168.3) min/day, 66.2% (21.9%)	ActivPAL3	Sitting (occupational and total day)	min/day & % of day	Total day: 59.4 (9.3)%, 563.1 (96.4) min/day; Occupational: 57.0 (15.7)%, 275.6 (90.0) min/day	NR	NR
Hekler, 2012	Older adults (65+)	CHAMPS	ST (TV, reading, socialize, passive travel, attend events)	min/week	1095.7 (486.4)	ActiGraph 7164 & 71256	ST	min/week	NR	r = 0.12, p<.001	2841.6 (LoA: -4476.7, -1206.5)
Herrmann, 2013	General population	GPAQ	Sitting	min/day	Low = 547.5 (179.2); moderate = 529.2 (173.4);	ActiGraph GT1M	ST	min/day	Low = 716.1 (128.5); moderate =	r = -0.12, NS	NR

First author, year	Population	Self-report measure				Device measure				Correlation	MD: Self-report – device*
		Name	Domain or type	Units	Mean (SD)*	Name	Domain or type	Units	Mean (SD)*		
					high = 550.7 (166.3)				658.5 (106.2); high = 600.8 (88.1)		
		IPAQ			Low = 512.8 (184.4); moderate = 413.2 (167.5); high = 477.7 (169.9)						
Hoos, 2012	Adult Latinas	GPAQ	Sitting	min/week	1130.14 (915.49)	ActiGraph GT1M	ST	min/week	3365.79 (530.43)	r = 0.28, NS	NR
Hur, 2019	Patients with fibrotic interstitial lung disease	IPAQ-LF	Sitting	min/day	Median (IQR): 360 (240-566)	ActiGraph GT3X	ST	min/day	Median (IQR): waist1 = 337 (256, 418) ; waist2 = 294 (219, 389); wrist1 = 54 (37, 96) ; wrist2 = 48 (33, 93)	wrist1 r = 0.10, p = 0.29; wrist2 r = 0.14, p = 0.15; waist1 r = 0.38, p<0.001; waist2 r = 0.40, p<0.001	NR
Hurtig-Wennlof, 2010	Elderly (65+)	modified IPAQ	Sitting	min/day	Median (IQR): women = 300 (240, 420); men = 420 (300, 600)	ActiGraph GT1M	ST	min/day	Median (IQR): women = 454 (399, 511); men = 518 (435, 565)	r = 0.277, p<0.05	NR
Hutchison, 2018	Older adults (with and without fibromyalgia)	SBQ	Total	min/week	FM = 68 (18), CO = 78 (16)	ActiGraph WGT3X	ST	min/day	FM = 238 (66), CO = 335 (78)	FM: r = -0.13, CO: r = 0.00	NR
Igelstrom, 2013	People with obstructive sleep apnea and obesity	IPAQ	Sitting	h/day	7h51min (2h59min)	SenseWear Pro 3 Armband	ST	h/day	9h55min (3h10min)	NR	114 (LoA: -362, 590) min/day
		Logbook	Sitting		8h21min (3h0min)						86 (LoA: -396, 568) min/day
Innerd, 2018	University staff or students	IPAQ-SF	Sitting	min/day	BMI normal = 476 (84), overweight = 545 (91), obese = 628 (83)	ActiGraph GT3X+	ST	min/day	BMI normal = 597 (67), overweight = 704 (73), obese = 823 (94)	r = 0.49, 95% CI: 0.45, 0.53	Normal = -121 (LoA: -227, 35), overweight = -159 (LoA: -345, 27), obese = -195 (LoA: -394, 5)

First author, year	Population	Self-report measure				Device measure				Correlation	MD: Self-report – device*
		Name	Domain or type	Units	Mean (SD)*	Name	Domain or type	Units	Mean (SD)*		
Ishii, 2018	Japanese adults	NR	Work and non-workday ST: passive travel; work; TV, videos and DVDs; computer, cell phone or tablet outside of work; in leisure time	min/day	Median (IQR) workday = 510.9 (330.0, 684.7); non-workday = 422.2 (301.3, 554.0), total = 506.7 (350.7, 636.4)	Active Style Pro HJA-350IT	ST	min/day	Median (IQR) workday = 524.7 (411.8, 614.6); non-workday = 498.4 (410.8, 578.7), total = 512.1 (429.8, 589.0)	Workday r = 0.57, p<.001, non-workday r = 0.23, p<.001, total r = 0.49, p<.001	Work day = -0.4 (LoA: -378.9, 378.1), non-work day = -49.2 (LoA: -477.7, 379.2), total = -13.4 (LoA: -361.9, 335.2)
Jancey, 2014	Office workers	OSPAQ	Occup. sitting	min/week	Occasion 1: 1917.9 (471.6); Occasion 2: 1917.6 (488.1)	ActiGraph GT3X+	ST	min/week	NR	r = 0.58 (95% CI: 0.33, 0.75), p<0.001	-25.4 (LoA: -784.7, 733.9)
Kim, 2018	University staff or students	Sedentary Behavior Record (SBR)	SBs	NR	NR	Autographer	SBs	NR	NR	NR	NR
Kim, 2017	General population	24-Hour Physical Activity Recall	SBs	min/day	Least square means = 450.3 (SE = 4.4)	SenseWear	ST	min/day	least square means = 654.3 (SE = 5.1)	r = 0.45, p<0.0001	NR
King, 2016	Underactive adults	EMA	Sitting	h/day	8.5 (2.4)	Smart phone accelerometer	ST	min/day	459.5 (86.9)	NR	NR
Knell, 2017	Community dwelling adults	EMA	SB	min/week	2320.2 (1998.8)	ActiGraph GT3X	ST	min/week	3400.8 (864.0)	r = 0.16 (p = 0.03), LCC = 0.07 (95% CI: -0.02, 0.16)	NR
		NHANES	TV + computer weekday + weekend		2862.3 (1676.4)					r = 0.07 (p = 0.33), LCC = 0.05 (95% CI: -0.06, 0.16)	
		IPAQ	Sitting		2897.1 (1559.6)					r = 0.08 (p = 0.27), LCC = 0.06 (95% CI: -0.05, 0.18)	
Kohler, 2017	Healthy, community dwelling adults	cpar24	SBs	min/24-h	median = 980	ActiGraph GT3X	ST	min/24-h	median = 1004	r = 0.54	Median diff = 7, p = 0.39, MD = −31 (LoA: -380, 319)

First author, year	Population	Self-report measure				Device measure				Correlation	MD: Self-report – device*
		Name	Domain or type	Units	Mean (SD)*	Name	Domain or type	Units	Mean (SD)*		
Kozey-Keadle, 2012	Overweight, non-exercising office workers	IPAQ-SF	Sitting	h/day	Weekday = 9.3 (3.3), weekend = 6.2 (3.1)	activPAL, ActiGraph GT3X	activPAL: sitting, ActiGraph: ST	% of wear time	activPAL: weekday = 68.8 (8.5), weekend = 60.4 (15.6), ActiGraph 100cpm weekday = 66.4 (10.2), weekend = 62.7 (8.9); ActiGraph 150 cpm weekday = 70.5 (9.4), weekend = 66.7 (9.0)	Weekdays: r = 0.41, ns; weekend: r = 0.55, p<0.05	Weekday = 40.5 (95% CI: -125.2, 22.3), weekend = 147.1 (95% CI: -228.3, -66.6)
		Domain-Specific Questionnaire (D-SQ)	Travel, TV, occup., computer at home, leisure time not including TV		12.6 (2.9), 12.1 (5.0)					Weekdays: r = 0.30, ns, weekends: r = 0.17, ns	Weekday = 176 (95% CI: 96.1, 256.9), weekend = 157.6 (95% CI: 22.1, 293.0)
Kozo, 2012	Community dwelling adults	IPAQ-LF	Sitting	min/day	364.9 (95% CI: 357.5, 372.4)	ActiGraph 7164 & 71256	ST	min/day	499.6 (95% CI: 495.1, 504.1)	NR	NR
Laeremans, 2017	Healthy adults	GPAQ	Sitting	min/day	Median = 535 (IQR: 420-635)	SenseWear	ST	min/day	Median (IQR) = 550 (481, 653)	r = 0.09	% difference median (IQR) = 8 (-12, 30), MD = 8 (LoA: -75, 92)
Lagersted-Olsen, 2014	Office workers	New questions - unnamed but based on IPAQ and MOSPAQ	Occup. sitting	h/day	Working hours = 5.6 (1.5), leisure time on workday = 3.1, leisure day = 4.6	ActiGraph GT3X+	ST	h/day	Working hours = 5.6 (1.1), leisure time on a work day = 3.5, leisure day = 7.0	Working hours r = 0.081, p = 0.699, leisure time on workday r = -0.185, p = 0.366, leisure day r = 0.100, p = 0.626	Work hours = 0 (LoA: -3.4, 3.4), leisure day = 2.4 (LoA: -7.8, 3.0)
Larsson, 2019	Validity = office workers, reliability = seniors	SED-GIH	Sitting	h/day	N/A - categorical response options	activPAL3 micro	Sitting time	h/day	9.7 (1.4)	r = 0.31, kappa = 0.12	NR
Legge, 2017	Patients with systemic lupus erythematosus	Framingham Physical Activity Index	SBs	min/day	SLE = 387.0 (233.2), RA = 416.8 (233.6), health = 468.0 (221.6)	ActiGraph GT3X+	ST	min/day	SLE = 603.6 (68.7), RA = 603.4 (72.5), healthy = 618.0 (93.5)	NR	NR
Lewis, 2016	Older adults	MARCA	SBs	min/day	559.2 (137.4)	activPAL3	Sitting	min/day	534.1 (114.1)	NR	NR
Lewis, 2018	Older cancer survivors and adults at	IPAQ - LF	Sitting	min/week	All = 3025 (1392), men = 3193 (1514),	ActiGraph GT3X	ST	min/week	all = 3919 (1380), men = 4051 (805),	r = 0.33, p<0.05, men r = 0.40, p<0.05, women r = 0.11, NS	% difference = 48.98%

First author, year	Population	Self-report measure				Device measure				Correlation	MD: Self-report – device*
		Name	Domain or type	Units	Mean (SD)*	Name	Domain or type	Units	Mean (SD)*		
	risk of cancer				women = 2742 (1139)				women = 3708 (757)		
Libertine, 2011	Overweight and obese office workers	Sitting Questionnaire	Sitting	h/day	NR	activPAL	Sitting/lying	h/day	NR	Weekday r = 0.22, weekend r = 0.34	Weekday = 2.8 (95% CI: 1.9, 3.6), weekend = 3.0 (95% CI: 1.1, 4.9)
		Focus Questionnaire								Weekday r = 0.10, weekend r = 0.42	Weekday = -0.91 (95% CI: -2.1, 0.30), weekend = -1.8 (95% CI: -3.0, -0.58)
Lopez-Rodriguez, 2017	Healthy elderly	EXERNET Questionnaire	Sitting	h/day	4.21 (1.33); men = 3.91 (1.04); women = 3.73 (1.18)	ActiGraph GT1M	ST	min/day	394.98 (83.98); men = 425.23 (94.97); women = 389.61 (81.55)	All r = 0.574, p<0.01; men r = 0.63, p<0.05; women r = 0.57, p<0.01	NR
Loprinzi, 2013	Healthy young adults	SBQ	ST	min/day	421.2 (95% CI: 384, 457)	ActiGraph GT1M	ST	min/day	518.3 (95% CI: 497, 539)	NR	NR
Lucas, 2013	College freshmen	IPAQ-LF	Sitting	h/day	NR	activPAL	Sitting/lying	h/day	NR	r = 0.33, p<.05	Weekday = -3.14, weekend = -4.36
Mader, 2006	Middle-aged women & men	IPAQ-SF	Sitting	min/week	2603 (1397)	ActiGraph AM7164	ST	min/week	3852 (629)	NR	NR
Maher, 2017	Community-dwelling older adults	Daily diary	Sitting/lying time	min/day	636.6 (219.9)	activPAL	Sitting/lying	min/day	573.7 (145)	NR	NR
Marmeleira, 2013	Adults who are blind	IPAQ-SF	Sitting	h/day	6.16 (2.42)	ActiGraph GT1M	ST	h/day	10.63 (1.5)	r = 0.36, p<0.01	-4.47 (LoA: -8.99, 0.06)
Marshall, 2010	Middle-aged women & men	Marshall Sitting Questionnaire	Sitting (travel, work, TV, computer, leisure)	min/day	NR	ActiGraph GT1M	ST	NR	NR	NR	Women: weekday = -63.6 (LoA: -395.6, 268.4), weekend = 10.8 (LoA: -398.0, 419.7)
Marshall, 2015	Community-dwelling older adults	Modified SBQ	ST (TV watching, computer/ internet use, reading, socializing, driving or using	h/day	Total = 11.4 (4.9), women = 11.6 (5.0), men = 11.1 (4.6)	ActiGraph GT3X+	ST	h/day	Total = 9.4 (1.5), women = 9.2 (1.1), men = 9.9 (1.2)	r = 0.22 (95% CI: 0.09, 0.34), CCC = 0.08 (95% CI: 0.02, 0.15)	2.06, p = 0.048

First author, year	Population	Self-report measure				Device measure				Correlation	MD: Self-report – device*
		Name	Domain or type	Units	Mean (SD)*	Name	Domain or type	Units	Mean (SD)*		
			motorized travel, doing hobbies, office or volunteer work, napping, and other behaviors)								
Matsuo, 2016	Workers	Workers' Sitting- and Walking-Time Questionnaire (WSWQ)	Occup. sitting, leisure sitting, non-workday sitting	min/day, percentage/day method	Median (IQR) Time 1 work sitting: T-method: 428 (360, 510), P-method: 496 (404, 574); non-work time sitting: T-method = 200 (120, 240), P-method = 202 (132, 284); non-work day sitting: T-method = 480 (300, 660), P-method = 576 (408, 683). Time 2 work sitting: T-method: 420 (330, 490), P-method: 470 (360, 540); non-work time sitting: T-method = 180 (120, 240), P-method = 215 (132, 264); non-work day sitting: T-method = 600 (300, 660), P-method = 603 (315, 714)	activPAL	Sitting/lying	min/day, %/day	Median (IQR) Time 1 work sitting: ActivPAL: 412 (352-502); non-work sitting 184 (137-230), non-workday sitting 590 (495-677).	Time 1 (pre-activPAL): work time r = 0.52 (time) & 0.59 (%); non-work time r = 0.55 (time) & 0.57 (%); non-workday r = 0.25 (time) & 0.42 (%) (All sig. at p<0.05 except non-workday time method). Time 2 (post-activPAL): work time r = 0.56 (time) & 0.65 (%); non-work time r = 0.51 (time) & 0.60 (%); non-work day r = 0.37 (time) & 0.53 (%) (all p<0.05)	Time 1: work time sitting MD time method = -7 (LoA: -254, 241), P-method = 35 (LoA: -200, 269). Non-workday sitting mean diff time method = -115 (LoA: -588, 358), P-method = -56 (LoA: -392, 281)
Matsuzaki, 2016	General population	Andhra Pradesh Children and Parent Study Physical Activity Questionnaire (APCAPS-PAQ)	Multiple SBs	min/day	All = 847 (206), men = 791 (188), women = 916 (207)	Actiheart	ST	min/day	NR	r = 0.26 (p<0.001), men r = 0.08 (p = 0.37), women r = 0.35, p<0.001	mean bias = -48 (SE = 14.6), men = -68 (SE = 20.4), women = -23 (SE = 20.5)

First author, year	Population	Self-report measure				Device measure				Correlation	MD: Self-report – device*
		**Name**	**Domain or type**	**Units**	**Mean (SD)***	**Name**	**Domain or type**	**Units**	**Mean (SD)***		
Matthews, 1995	University students	Physical Activity Log (PAL)	ST	min/day	538.3 (113.3)	Tritrac R3D	ST	min/day	753.7 (90.5)	NR	NR
Matthews, 2013	Middle-aged women & men	Previous Day Recall (PDR)	ST	h/day	All = 10.1 (2.91), men = 9.88 (3.21), women = 9.94 (2.51)	activPAL, ActiGraph GT3X+	Sitting/lying, ST	h/day	activPAL: 9.0 (1.90), men 9.16 (2.09), women 9.19 (1.91), ActiGra ph: 9.1 (1.71) men	activPAL vs. PDR: men r = 0.67, women r = 0.34	Men = 0.72 (LoA: -2.61, 4.05), women = 0.75 (LoA: -2.21, 3.71)
Matthews, 2018	General population	AARP questionnaire	ST	h/day	8.0 (3.3)	activPAL, ActiGraph GT3X+	Sitting/lying, ST	h/day	activPAL: 9.8 (1.8), men = 10.2 (1.7), women = 9.4 (1.7), ActiGraph (100 cpm) = 9.7 (1.6), ActiGraph (200 cpm) = 8.0 (1.7)	NR	-1.8 (3.5)
		24-hour recall (ACT24)			9.9 (2.2)						0.1 (2.0)
Matthews, 2011	Adults with intellectual disabilities	IPAQ-SF	Sitting	h/day	9.36 h (3.21)	ActiGraph GT1M	ST	h/day	10.17 (SD, 2.06)	NR	-0.81 (SD = 3.80, LoA: -8.41, 6.79)
Matton, 2007	Employed/unemployed adults, retired adults	Flemish Physical Activity Computerized Questionnaire (FPACQ)	TV	h/week	Unemployed/ employed men = 17.52 (9.07), women = 12.85 (7.39). Retired: men = 22.33 (11.43); women = 18.06 (9.50)	Triaxial RT3	TV time (overlaid from log)	h/week	Unemployed/ employed men = 19.54 (11.90), women = 15.76 (8.14). Retired: men = 29.15 (14.53); women = 21.76 (10.84)	Unemployed/ employed men = 0.69 (p<0.001), women = 0.83 (p<.001). Retired: men = 0.78 (p<.001); women = 0.80 (p<.001)	NR
Mazzoni, 2017	Breast cancer patients	Log book	ST	min/day	517 (185), 95% CI: 494, 540	SenseWear Armband Mini	ST	min/day	713 (128), 95% CI: 697, 729	r = 0.40 (p<.01)	-196 (LoA: ±408)
McNeil, 2018	Post-menopausal women	SIT-Q	Total, occup. and leisure ST	h/day	High = 10.7 (3.6), moderate = 11.0 (3.4)	ActiGraph GT3X+	ST	h/day	High = 8.7 (1.5), moderate = 8.9 (1.6)	NR	NR
Melville, 2011	adults with intellectual disabilities and obesity	IPAQ-S	Sitting	min/day	557.4 (189.4)	ActiGraph GT1M	ST	min/day	612.0 (121.75)	NR	NR

First author, year	Population	Self-report measure				Device measure				Correlation	MD: Self-report – device*
		Name	Domain or type	Units	Mean (SD)*	Name	Domain or type	Units	Mean (SD)*		
Menezes, 2017	People who are deaf	IPAQ-S	Sitting	h/day	7.6 (2.7)	ActiGraph GT1M	ST	h/day	10.1 (1.6)	r = 0.13 (NS)	-2.56 (LoA: -8.21, 3.09)
Mensah, 2016	Healthy general population	STAQ	Total sitting, occup., travel, leisure	h/week	Total sitting = 46.5 (23.2)	ActiGraph GT3X+	ST	h/week	NR	Total r = 0.54, p<.0001, work: r = 0.88, p<.0001, transport r = 0.35, p =0.001, leisure r = 0.19, p = 0.09	Total sitting = -17 (LoA: -43.8, 34.6), work = 2.6, transport = -1.4, leisure = 17.6
Metcalf, 2018	General population	GPAQ	Sitting	min/day	Training = 332.7 (208.0), testing = 363.2 (210.4), validation = 294.9 (201.0)	ActiGraph GT9X	ST	min/day	Training = 535.1 (117.8), testing = 574.6 (154.4), validation = 510.7 (211.1)	NR	NR
Meyer, 2015	Patients with depression	IPAQ	Sitting	min/day	546.5 (212.3)	ActiGraph GT3X+	ST	min/day	692.6 (93.5)	r = 0.550, p = 0.005	NR
Moran, 2016	Older adults	IPAQ-SF	Sitting	h/day	Assisted living: 10.5 (1.3), independent living: 10.0 (2.1)	SenseWear Armband	ST	h/day	Assisted living: , independent living:	NR	-269 min/day (LoA: -535, -3)
Moss, 2018	People with intellectual disabilities	IPAQ-SF (proxy reported by caregivers)	Sitting	NR	NR	ActiHeart	ST	MET/min	NR	r = 0.31, p = 0.04	740.0 (SD=0.02) and 62.65 (SD=0.01)
Mumu, 2017	Healthy adults	GPAQ	Sitting	min/day	Median (IQR) = 120 (60; 180)	ActiGraph GT3X+	ST	min/day	Mean (SD) = 551.15 (83.03), median (IQR) = 546.03 (494.73; 607.05)	r = 0.23, p <.001	Urban = -419.63 (LoA: -659.55, -179.71), rural = -415.0 (LoA: -577.02, -252.98)
Murillo-Rabago, 2010	Healthy adults	IPAQ-SF & IPAQ-LF	Sitting	NR	NR	ActiGraph GT1M	ST	NR	NR	IPAQ-SF r = 0.30, p=0.05; IPAQ-LF r = 0.33, p<0.03	NR
O'Neill, 2017	Patients with Bronchiectasis	IPAQ-LF	Sitting	min/day	362 (115)	ActiGraph GT3X	ST	min/day	634 (77)	NR	-160 (SD=171, LoA: 175, -495)
Oostdam, 2013	Overweight and obese pregnant women	Activity Questionnaire for Adults and Adolescents (AQuAA)	SBs	min/week	Median (min, max) = 2460 (0, 6780)	Actitrainer	ST	min/week	Median (min, max) = 4361 (2984, 5717)	r = 0.23	NR

First author, year	Population	Self-report measure				Device measure				Correlation	MD: Self-report – device*
		**Name**	**Domain or type**	**Units**	**Mean (SD)***	**Name**	**Domain or type**	**Units**	**Mean (SD)***		
Otten, 2010	Overweight and obese adults	None	TV	h/day	4.3 (1.3)	TV monitoring device	TV time	h/day	4.9 (2.6)	r = 0.54, p<0.001	-0.6
Oviedo-Caro, 2018	Pregnant women	SBQ	SBs	h/day	Average = 8.7 (3.5), weekday = 8.8 (3.9), weekend = 8.7 (3.6)	SenseWear	ST	h/day	Average = 10.0 (2.1), weekday = 10.1 (2.3), weekend = 9.9 (2.4)	Weekday = r = 0.23 (p = 0.001), average = 0.20 (p = 0.008), weekend = (r = 0.12, p = 0.103)	Weekday = −1.33 (3.99), weekend = −1.20 (4.07), average = −1.29 (3.74)
Oyeyemi, 2014	General population	Hausa IPAQ-SF	Sitting (weekday)	min/day	Total = 230 (136), men = 210 (133), women = 260 (137)	ActiGraph 7164	ST	min/day	Total = 409 (102), men = 405 (109), women = 415 (91)	All = 0.06, men = -0.08, women = 0.14, all NS	2180 (p = 0.13, 95% LoA = 2510, 150)
Pedersen, 2016	Desk-based workers	OSPAQ	Occup. sitting	% of workday	62.56% (22.61%)	activPAL	Sitting	% of workday	65.72% (21.76)	r = 0.90	Bias = -3.16 % (SD = 9.32 %, LoA = -21.4, 15.1)
Pedisic, 2014	Desk-based workers	Workplace Sitting Breaks Questionnaire (SITBRQ)	Duration and frequency of sitting breaks at work	breaks/h, min/day	NR	ActiGraph GT1M	Duration and frequency of sitting breaks at work	breaks/h, min/day	NR	Frequency of breaks (breaks/hour) 0.24 (95% CI: 0.07 - 0.40). Total duration of breaks (min/day) 0.05 (95% CI: -0.12, 0.22)	NR
Peters, 2010	Urban adults	Past Year Physical Activity Questionnaire	SBs	min/day	Median (IQR): all = 344.8 (248.3, 466.3), men = 356.7 (271.9, 496.6), women = 325.1 (241.7, 433.5)	ActiGraph 7164	ST	min/day	Median (IQR): all = 507.8 (448.6, 568.1), men = 517.4 (462.0, 584.4), women = 498.3 (437.5, 556.5)	r = 0.16, p<.001	-152.3 (95% CI: -166.2, -138.4, LoA: -484.0, 179.4)
Pinto, 2017	Breast cancer survivors	7-d PAR	Sitting	min/week	3258.08 (1030.33)	ActiGraph GT3X	ST	min/week	4612.07 (760.72)	r = 0.37, p = 0.002	NR
Powell, 2011	Overweight/ obese Latina immigrants	GPAQ	Sitting	NR	NR	NR	ST	NR	NR	r = 0.09, p = 0.60	NR
Prince, 2018	Nurses	IPAQ-SF	Sitting	min/day	301 (192)	ActiGraph GT3X+	ST	min/day	445 (109)	r = 0.43, p<.001	NR
		SBQ	SBs		365 (178)					r = 0.31, p < 0.001	
Prince SA. 2019	Cardiac rehabilitation patients	IPAQ-SF	Sitting	min/day	487.5 (193.2)	ActivPAL3	Sitting + lying	min/day	497.3 (107.9)	r = 0.05, P = 0.79;	9.7 (95% CI: -67.7, 87.2, p = 0.800)

First author, year	Population	Self-report measure				Device measure				Correlation	MD: Self-report – device*
		Name	Domain or type	Units	Mean (SD)*	Name	Domain or type	Units	Mean (SD)*		
Rees-Punia, 2019	General population	7-day diary	Sitting	min/day	Total = 557.3 (135.3), women = 540.8 (126.7), men = 581.3 (144.0)	ActiGraph GT3X	ST	min/day	Total = 579.7 (96.7), men = 597.4 (95.9), women = 567.5 (95.5)	r = 0.56 (95% CI: 0.51, 0.61)	NR
		Sitting questionnaire			Total = 450.7 (173.2), women = 453.7 (180.4), men = 446.2 (162.2)					r = 0.41 (95% CI: 0.35, 0.47)	
Riviere, 2018	General population	GPAQ (French)	Sitting	min/day	588.6 (146.4)	ActiGraph GT3X	ST	min/day	843.6 (134.5)	r = 0.42, p < 0.01	251.2 (SD = 161.1, LoA: 90.1, 412.3)
Roman-Viñas, 2010	General population	IPAQ-LF	Sitting	min/day	373.1 (184.3)	ActiGraph MTI	ST	min/day	481.9 (121.1)	r = 0.34, p<.05	NR
Rosenberg, 2010	validity = overweight men & women	SBQ	SBs	Total = h/week, weekday/ weekend-specific = h/day	Total: women = 64.6 (26.7), men = 66.6 (24.9), weekday: women = 10.3 (4.6), men = 9.0 (3.9), weekend day: women = 8.8 (3.9), men = 10.8 (4.0)	ActiGraph 7164	ST	NR	NR	Men: r = –0.01 (0.81), women: r = 0.10 (0.07)	NR
Rosenberg, 2016	Older adults in retirement communities	SBQ	SBs	h/day	11.0 (4.9)	ActiGraph GT3X	ST	h/day	8.6 (1.0)	NR	NR
Rosenberg, 2015	Overweight and obese older adults	IPAQ-LF	Sitting	h/day	Weekday = 8.38 (2.54), weekend day = 7.48 (2.50)	activPAL	Sitting	h/day	9.22 (1.70)	NR	NR
Ruiz-Casado, 2016	Spanish cancer survivors	IPAQ-SF	Sitting	min/week	1793 (914)	ActiGraph GT3X	ST	min/week	3583 (1065)	r = 0.07	-1824 (SD = 1308, LoA: -4367, 739)
		GPAQ			1635 (947)					r = 0.17	-1988 (SD = 1231, LoA: -4400, 425)
Ruiz-Casado, 2015	Spanish cancer survivors	IPAQ-SF	Sitting	min/day	NR	ActiGraph GT3X	ST	min/day	NR	r = 0.29	-297

First author, year	Population	Self-report measure				Device measure				Correlation	MD: Self-report – device*
		Name	Domain or type	Units	Mean (SD)*	Name	Domain or type	Units	Mean (SD)*		
Ryan, 2014	General population	Health Survey for England	Sitting	min/day	Middle-aged/ include: 603 (272), excluded: 609 (310), older/included: 754 (293), excluded: 814 (351)	ActiGraph GT1M	ST	min/day	Middle-aged/ included: 576 (86), excluded: 581 (98), older/ included: 613 (83), excluded: 619 (88)	NR	NR
Ryan, 2018	Community dwelling older adults	IPAQ-LF	Sitting	h/week	39.7 (14.9)	GeneActiv Original	ST	h/week	67.3 (10.5)	r = 0.29	NR
Ryde, 2017	Women attending Bingo club	Marshall Sitting Questionnaire	Total sitting: travel + work + TV + home computer + leisure time (excluding TV)	h/day	Weekday = 7.7 (3.6), weekend day = 8.0 (3.6)	ActiGraph GT3X	ST	h/day	9.6 (1.7)	NR	NR
Sasaki, 2018	People with Multiple Sclerosis	Marshall Sitting Questionnaire	Total sitting: travel + work + TV + home computer + leisure time (excluding TV)	min/day	652.7 (258.0) adjusted for 12h	ActiGraph GT3X	ST	min/day	493.7 (64.2) adjusted for 12h	r = 0.34, p<.01	Mean bias = 80.54 (LoA: -410.5, 571.5)
Schaller, 2016	Low back pain patients & healthy controls	GPAQ	Sitting	min/day	454 (214)	ActiGraph GT3X	ST	min/day	577 (103)	NR	-122.2 (LoA: -502.5, 258.1)
Scheers, 2012	Workers	Flemish physical activity computerized questionnaire (FPACQ)	Sitting	h/week	37.85 (16.65)	SenseWear Pro 3	ST	h/week	62.86 (12.61)	r = 0.54, p<.001	-25.01
Segura-Jimenez, 2013	Women with fibromyalgia	IPAQ-LF	Sitting, Sitting + travel	min/day	sitting = 319 (171), sitting + travel = 359 (195)	SenseWear Pro 3	ST	min/day	907 (136)	Sitting: r = -0.12, sitting + travel: r = -0.11	NR
Shadyab, 2017	Older women	No name	ST (sitting + lying)	h/day	8.6 (4.3)	ActiGraph GT3X	ST	h/day	9.2 (SD, 1.5)	NR	NR

First author, year	Population	Self-report measure				Device measure				Correlation	MD: Self-report – device*
		Name	Domain or type	Units	Mean (SD)*	Name	Domain or type	Units	Mean (SD)*		
Shuval, 2014	Primary care patients	Rapid Assessment Disuse Index (RADI)	Sitting (home/work, TV/ videos/ DVDs, computer at home/work, eating meals, etc.)	NR	NR	ActiGraph GT3X	ST	NR	NR	Past week (controlling for wear time) = 0.291, p<0.01, past month = 0.189, p<.05, past year = 0.245, p<.01	NR
Simpson, 2015	Healthy adults	Question 8 of the Paffenbarger Physical Activity Questionnaire	Sitting	NR	NR	Actical	ST	NR	NR	0.20 (95% CI: 0.14, 0.33)	NR
Stolberg, 2018	Patients undergoing gastric bypass	Recent Physical Activity Questionnaire	SBs	min/day	342.9 (181.1)	ActiGraph GT3X	ST	min/day	603.5 (68.8)	NR	NR
Sudholz, 2018	Workers	None	Sitting at work	min/day	Median (IQR) = 420 (360, 450)	activPAL, ActiGraph GT3X	Sitting time, ST	min/day	median (IQR): ActivPAL = 346.81 (281.46, 414.31), ActiGraph = 430.94 (395.11, 451.38)	activPAL = 0.24 (95% CI: -1.0, 0.47), ActiGraph = 0.39 (0.15, 0.68)	NR
Sushames, 2015	Indigenous Australians	Past Day Adults' ST Questionnaire	SBs	min/day	Indigenous = 526 (23.9), non-Indigenous = 575 (82.6)	ActiGraph GT3X	ST	min/day	Indigenous = 350 ± 51.4, non-Indigenous = 390 ± 45.3	p<.001	NR
Sweatt, 2015	Latina Immigrants	GPAQ	Sitting	min/day	186.14 (105.04)	ActiGraph MTI	ST	min/day	575.46 (93.67)	r = 0.06, p = 0.68	NR
Terada 2015	General population	Activity Log Diary	SBs	min/day	512.1 (95.2)	SenseWear Pro 3	ST	min/day	475.2 (82.4)	r = 0.68, p<.01	36.9 (LoA: -103.3, 177.1)
Toledo, 2017	US veterans & university employees	BeWell24 App	SBs	min/day	NR	activPAL3	Sitting/lying	min/day	695.5 (139.3)	ICC = 0.35 (95% CI: 0.04, 0.56)	-160.4 (LoA: -179.8, -141.0)
Umstattd Meyer, 2013	Free living adults	IPAQ	Sitting	min/day	502.1 (95% CI: 468.7, 535.4)	ActiGraph GT1M	ST	min/day	NHANES data reduction = 667.4 (650.0, 685.0), Crouter2 = 525.8 (507.0,	NR	NR

First author, year	Population	Self-report measure				Device measure				Correlation	MD: Self-report – device*
		Name	Domain or type	Units	Mean (SD)*	Name	Domain or type	Units	Mean (SD)*		
									544.6), Rev Crouter2 = 640.5 (622.9, 658.1)		
Unick, 2017	Young adults	SBQ	SBs	h/day	10.1 (3.5)	SenseWear	ST	h/day	9.1 (1.8)	*r* = 0.27, *p* < 0.001	-0.9 (SD=3.5)
Urda, 2017	Office workers	Paffenbarger Physical Activity Questionnaire (PPAQ)	Total ST (sitting/lying /sleeping)	h/24-h	14.65 (2.77)	activPAL3	Sitting/lying	hours/24-h	17.71 (1.46) h/24h, 5.69 (1.06) h/8.5-h working day	r = 0.253; p = 0.098	NR
		OSPAQ	Occup. sitting	h/8.5 workday	5.96 (1.11)					r = 0.100; p = 0.518	
Van Cauwenberg, 2014	Free-living older adults	NR	SBs (TV, computer, reading, sedentary hobbies, seated conversation or listening to music, telephone, travel, sitting for household chores, resting, and eating)	min/day	Median (IQR) = 475.0 (383.0, 599.0)	ActiGraph GT3X	ST	min/day	580.4 (97.7)	r = 0.30, men: r = 0.35, women: r = 0.24	All = -81.88 (95% LoA: -364.16; 200.41), men = -103.55 (-375.36; 168.26), women: = -66.59 (LoA: -352.67; 219.49)
Van Der Ploeg, 2010	Workers	2006 Australian Bureau of Statistics time use survey	SBs	min/day	Household = 144 (91), leisure = 213 (172), transportation = 164 (138), total non-occup. = 523 (246)	ActiGraph GT1M	ST	min/day	Household = 210 (104), leisure = 164 (133), transportation = 120 (94), total non-occup. = 495 (194)	Household = 0.39 (p<.05), leisure = 0.56 (p<.05), transportation = 0.50 (p<.05), non-occup. = 0.57 (p<.05)	NR
Van Dyck, 2015	General population	IPAQ-LF	Sitting	min/day	413.1 (157.6)	ActiGraph 7164	ST	min/day	506.8 (114.1)	r = 0.67, p<.001	93.7 (LoA: -197.9, 385.2)
Van Dyck, 2010	General population	IPAQ-LF	Sitting	min/day	421.6 (158.2)	ActiGraph 7164	ST	min/day	504.8 (117.5)	NR	NR

First author, year	Population	Self-report measure				Device measure				Correlation	MD: Self-report – device*
		Name	Domain or type	Units	Mean (SD)*	Name	Domain or type	Units	Mean (SD)*		
van Nassau, 2015	Health agency employees	OSPAQ	Occup. sitting	min/work day	391 (88)	activPAL3, ActiGraph GT1M & GT3X	ActivPAL: sitting time, ActiGraph: ST	min/day	ActivPAL = 347 (58), ActiGraph = 348 (54)	r = 0.37, p<0.05,	Exact numbers not reported.
		WSQ	Workplace sitting		405 (72)					r = 0.25, NS	
Vandezande, 2014	Overweight and obese adults	SBQ	SBs (sum of questions)	h/day	10.96 (5.16)	ActiGraph	ST	h/day	8.65 (1.69)	NR	NR
Vandoni, 2017	Highschool students (median age = 18)	IPAQ-LF	Sitting	min/day	Median (IQR) = 480.0 (420.0, 540.0)	ActiHeart	ST	min/day	Median (IQR) = 660.50 (542.50, 763.40)	r = -0.0199, p = 0.917	209.2 (LoA: -284.79, 676.0)
Vanroy, 2014	Stroke patients	Activity Diary	SBs (≤ 1 MET)	min/day	Median (IQR): 342.0 (247.5, 378.0)	SenseWear Pro2	ST	min/day	Median (IQR): 293.52 (209.24, 437.95)	r = 0.16, p = 0.567	NR
Veitch, 2018	Orthopedic trauma patients	IPAQ	Sitting	h/day	Median = 8.0	activPAL	Sitting	h/day	Median = 10.59	r = 0.384, p < 0.001	NR
		Australian Diabetes, Obesity and Lifestyle General Questionnaire 3 (AusDiab3)	SB		Median = 9.21					r = 0.551, p < 0.001	
Visser, 2013	older adults	LASA SB Questionnaire	Sum of SBs (napping, reading, listening to music, watching TV, computer, working, hobbies, talking, transportation, church/ theater)	h/day	Average = 10.4 (3.5), weekday = 10.2 (3.8), weekend = 10.8 (3.5)	ActiGraph GT3X	ST	h/day	Average = 10.2 (1.2), weekday = 10.1 (1.2), weekend day = 10.3 (1.4)	r = 0.36, p<.01	2.1 (LoA: -7.40, 3.25)
Wanner, 2017	General population	GPAQ	Sitting	h/week	Total = 51.5 (24.5), men = 54.5 (25.1), women = 48.5 (23.6)	ActiGraph GT3X	ST	h/week	Total = 61.8 (8.4), men = 63.7 (8.4), women = 59.8 (8.0)	r = 0.47, p<.001, men: r = 0.44, p<.001, women: r = 0.49, p<.001	10.3 (LoA: -32.7, 53.2)

First author, year	Population	Self-report measure				Device measure				Correlation	MD: Self-report – device*
		Name	Domain or type	Units	Mean (SD)*	Name	Domain or type	Units	Mean (SD)*		
Wanner, 2016	General population	IPAQ-LF	Sitting + motorized transport	h/week	Sitting only = 37.0 (19.8), men = 37.7 (20.6), women = 36.5 (19.1); sitting + motorised transport: 43.2 (20.5), men = 44.1 (21.8), women = 42.5 (19.4)	ActiGraph GT3X	ST	h/week	Total = 69.3 (11.0), men = 71.4 (11.0), women = 67.6 (10.7)	All r = 0.42, p<.001, men: r = 0.28, p<.001, women: r = 0.53, p<.001	26.4 (LoA: -12.0, 64.9)
Waters, 2016	Office workers	SB in Business School Employees Questionnaire	Sum of SBs (work, transport, leisure including TV, napping, eating, online, other)	min/day	Median (IQR): workdays = 870.0 (338.8), non-workdays = 650.0 (365.0)	ActiGraph GT3X	ST	min/day	Median (IQR): workdays = 667.0 (174.5), non-work days = 557.0 (158.0)	NR	NR
Watson, 2017	Pregnant women	GPAQ	Sitting	min/day	Median (IQR): 300 (120, 480)	ActiGraph GT3X	ST	min/day	Median (IQR): 457 (380.4, 546.5)	NR	127.5 (95%CI: -299.2, 554.2) at 14–18 weeks gestation and 89.2 (95%CI:-390.7, 569.2) at 29–33 weeks gestation, indicating that the GPAQ underestimates the amount of SB by an average of 127.5 min/day in the second trimester and 89.2 min/day in the third trimester.
Welch, 2018	Breast cancer survivors	IPAQ-LF	Sitting	min/day	303.8 (163.4)	ActiGraph GT1M	ST	min/day	603.9 (78.0)	r = 0.26, P < .001	300.1 (95% CI: 283.6, 317.0, p<.001)
		Sitting Time Questionnaire	Sum of SBs		605.2 (296.2)					r = 0.30, P < .001	8.1 (95% CI: -35.5, 19.4, p = 0.56)
Whitfield, 2013	Recreational runners	Multicontext Sitting Time Questionnaire	Weekend & week day sitting across contexts	min/day	Workday = 659.2 (221.2), non-workday = 568.2 (228.9)	ActiGraph GT1M	ST	min/day	Workday = 550.9 (89.1), non-workday = 478.6 (80.1)	Workday r = 0.16, p = 0.48, non-workday r = 0.44, p = 0.09	NR

First author, year	Population	Self-report measure				Device measure				Correlation	MD: Self-report – device*
		Name	Domain or type	Units	Mean (SD)*	Name	Domain or type	Units	Mean (SD)*		
Wick, 2016	Office workers	OSPAQ	Occup. sitting	% of workday	73.2 (18.9)	ActiGraph GT3X	ST	% of workday	77.1 (11.1)	ICC = 0.51 (95% CI: 0.24; 0.71)	-3.9 (SD = 15.1)
Wijndaele, 2014	General population	SIT-Q-7d	Total sitting (meals, transport, occup., non-occup. screen time, and other ST)	h/day	Sample 1 = 10.93 (4.35), sample 2: total = 9.20 (4.37), men = 9.94 (4.43), women = 8.98 (4.29)	Sample 1: activPAL3, sample 2: Actiheart	1) Sitting, 2) ST	h/day	Sample 1 = 9.35 (2.69), sample 2 (used accel method): total = 10.04 (2.29), men = 10.54 (1.97), women = 9.62 (1.76)	Sample 1: r = 0.52, p<.01, sample 2: total r = 0.37, p<.001, men r = 0.32, p<.001, women r = 0.35, p<.001	Sample 1 = 0.98 (4.25), Sample 2: total = -0.73 (3.71), p<.001, men = -0.59 (3.66), p<.001, women = -0.84 (3.77), p<.001
Yi, 2014	General population	Physical Activity Transit Survey	Daytime and evening sitting	min/day	444	NR	ST	min/day	494	r = 0.32, p<0.01	49 (LoA: -441, 343)
Yu,2014	Rheumatoid arthritis patients	IPAQ	Sitting	min/day	343 (141)	NR	ST	min/day	583 (98)	r = 0.29, p<0.05	NR

Accel – accelerometer, CI – confidence interval, GPAQ – Global Physical Activity Questionnaire, IPAQ-LF – International Physical Activity Questionnaire Long Form, IPAQ – SF – International Physical Activity Questionnaire Short Form, LoA –limits of agreement, MD – mean difference, NR –not reported, occup. – occupational, OSPAQ – Occupational Sitting Physical Activity Questionnaire, SBQ – Sedentary Behaviour Questionnaire, SD –standard deviation, SIT-Q-7d – Last 7-day sedentary behavior questionnaire, ST – sedentary time, WSQ – Workforce Sitting Questionnaire

*Mean (SD) unless otherwise noted.
