# Supplementary material for: A comparison of self-reported and device measured sedentary behaviour in adults: a systematic review and meta-analysis
Source: Int J Behav Nutr Phys Act. 2020 Mar 4;17:31. doi: 10.1186/s12966-020-00938-3 (PMC7055033; doi:10.1186/s12966-020-00938-3)
Supplement: Supplementary file 17 — Additional file 17: Supplemental table 4. Reliability characteristics. [file 12966_2020_938_MOESM17_ESM.pdf]

Supplemental table 4. Reliability characteristics

First author, year	Population	Self-report measure		Reliability assessment		
		Name	Domain or type	Time between recall	Statistic used	Results
Aguilar-Farias, 2015	Community-dwelling older adults	SB Question	Sitting	Unclear	ICC	Average = 0.79 (SEM 1.03), weekday = 0.80 (SEM 1.04), weekend day = 0.78 (SEM 1.10)
		MARCA	ST			Day before yesterday = 0.72 (SEM 1.18), yesterday = 0.96 (SEM 0.47)
Alkahtani, 2016	Male university students	GPAQ	Sitting	NR	Spearman	0.70, p <0.01
Anjana, 2015	Urban and rural populations	MPAQ	TV, sitting	1 month	ICC	Sitting = 0.81, TV viewing = 0.67
Bonn, 2015	Men who underwent Prostate Specific Antigen testing	Active-Q	SBs	3 weeks	ICC	0.80 (95% CI: 0.74, 0.86)
Busschaert, 2015	General population	last-7-day SB questionnaire (SIT-Q-7d)	Sitting/lying down	>6 days	ICC	Adults = 0.22, older adults = -0.13
Cerin, 2012	Urban older adults	IPAQ - LF	Sitting	2 weeks	ICC	0.77 (95% CI: 0.66, 0.83)
Chasan-Taber, 2004	Pregnant women	Pregnancy Physical Activity Questionnaire (PPAQ)	SBs (household/care giving, occupational, sports/ exercise, transportation, and inactivity	one week	ICC	0.79
Chau, 2012	Workers	OSPAQ	Occupational sitting	1 week	ICC	0.89 (95% CI: 0.83, 0.92)
Chau, 2011	Workers	WSQ, IPAQ	WSQ: Occupational sitting, TV, computer at home, leisure activities, travel on workdays and non-workdays. IPAQ = sitting	7 days	ICC	**WSQ: ALL WORKDAY:** Transport ICC = 0.67 (95% CI: 0.54, 0.77); at work ICC = 0.63 (95% CI: 0.49, 0.74); watching TV ICC = 0.91 (95% CI: 0.87, 0.94); computer at home ICC = 0.56 (95% CI: 0.40, 0.69); other leisure activities ICC = 0.68 (95% CI: 0.55, 0.78); total all domains ICC = 0.65 (95% CI: 0.51, 0.75). **WSQ: ALL NON- WORKDAY:** Transport ICC = 0.60 (95% CI: 0.45, 0.72); at work ICC = 0.50 (95% CI: 0.33, 0.64); watching TV ICC = 0.79 (95% CI: 0.69, 0.85); computer at home ICC = 0.81 (95% CI: 0.73, 0.87); other leisure activities ICC = 0.59 (95% CI: 0.44, 0.71); total all domains ICC = 0.80 (95% CI: 0.72, 0.87). **WSQ: ALL AVERAGE TOTAL WORK+NONWORK DAYS** ICC = 0.76 (95% CI: 0.66, 0.83). **WSQ: WOMEN: WORKDAY**: Transport ICC = 0.69 (95% CI: 0.53, 0.81); at work ICC = 0.79 (95% CI: 0.66, 0.87); watching TV ICC = 0.95 (95% CI: 0.91, 0.97); computer at home ICC = 0.59 (95% CI: 0.39, 0.74); other leisure activities ICC = 0.74 (95% CI: 0.59, 0.84); total all domains ICC = 0.77 (95% CI: 0.65, 0.86). **WSQ: WOMEN NON-WORKDAY:** Transport ICC = 0.63 (95% CI: 0.45, 0.77); at work ICC = 0.57 (95% CI: 0.37, 0.72); watching TV ICC = 0.76 (95% CI: 0.62, 0.85); computer at home ICC = 0.79 (95% CI: 0.66,

First author, year	Population	Self-report measure		Reliability assessment		
		Name	Domain or type	Time between recall	Statistic used	Results
						0.87); other leisure activities ICC = 0.76 (95% CI: 0.62, 0.85); total all domains ICC = 0.85 (95% CI: 0.76, 0.91). **WSQ: WOMEN AVERAGE TOTAL** ICC = 0.90 (95% CI: 0.84, 0.94). **WSQ: MEN: WORKDAY:** Transport ICC = 0.56 (95% CI: 0.27, 0.75); at work ICC = 0.51 (95% CI: 0.21, 0.72); watching TV ICC = 0.86 (95% CI: 0.70, 0.93); computer at home ICC = 0.53 (95% CI: 0.24, 0.73); other leisure activities ICC = 0.51 (95% CI: 0.21, 0.72); total all domains ICC = 0.46 (95% CI: 0.16, 0.69). WSQ: **MEN NON-WORKDAY:** Transport ICC = 0.56 (95% CI: 0.28, 0.75); at work ICC = 0.29 (95% CI: -0.04, 0.56); watching TV ICC = 0.81 (95% CI: 0.64, 0.91); computer at home ICC = 0.83 (95% CI: 0.69, 0.91); other leisure activities ICC = 0.23 (95% CI: -0.12, 0.53); total all domains ICC = 0.57 (95% CI: 0.30, 0.76). **WSQ: MEN AVERAGE TOTAL** ICC = 0.57 (95% CI: 0.30, 0.76). **IPAQ TOTAL SITTING:** Weekday ICC = 0.69 (95% CI: 0.56, 0.78); weekend ICC = 0.65 (0.51, 0.76); average total ICC = 0.73 (0.61, 0.81). **IPAQ WOMEN SITTING:** Weekday ICC = 0.72 (95% CI: 0.57, 0.83); weekend ICC = 0.72 (0.56, 0.82); average total ICC = 0.77 (0.62, 0.86). **IPAQ MEN SITTING:** Weekday ICC = 0.63 (95% CI: 0.36, 0.80); weekend ICC = 0.47 (0.15, 0.70); average total ICC = 0.67 (0.42, 0.83).
Chinapaw, 2009	Workers	Activity Questionnaire for Adults and Adolescents (AQuAA)	SBs	2 weeks	ICC	0.60 (95% CI: 0.40, 0.74)
Chu, 2018	University desk workers	ASBQ, GPAQ	ASBQ: occupational; transportation; leisure-time; other. GPAQ: sitting	7 days	Spearman r; ICC	GPAQ r = 0.71 (95% CI: 0.62, 0.82), ICC = 0.73 (95% CI: 0.61, 0.82). ASBQ sum r = 0.61 (95% CI: 0.46, 0.73), ICC = 0.72 (95% CI: 0.57, 0.82), ASBQ occup r = 0.82 (95% CI: 0.74, 0.88), ICC = 0.78 (95% CI: 0.66, 0.86); ASBQ transport r = 0.71 (95% CI: 0.58, 0.80), ICC = 0.68 (95% CI: 0.51, 0.79); ASBQ eating r = 0.59 (95% CI: 0.43, 0.71), ICC = 0.73 (95% CI: 0.58, 0.82); ASBQ TV r = 0.78 (95% CI: 0.68, 0.85), ICC = 0.82 (95% CI: 0.73, 0.88); ASBQ leisure computer r = 0.67 (95% CI: 0.53, 0.77), ICC = 0.59 (95% CI: 0.43, 0.71); ASBQ other leisure r= 0.41 (95% CI: 0.23, 0.58), ICC = 0.38 (95% CI: 0.18, 0.55)
Clark, 2013	Breast cancer survivors	Past-day Adults' Sedentary Time (PAST)	Single weekday: time spent sitting or lying while at work, travelling, watching TV, computer (excluding work), reading (excluding work), hobbies, and any other purposes not reported in the previous items.	7 days	Composite score: ICC; individual domains spearman	Composite sum ICC = 0.50 (95% CI: 0.32, 0.64); work r = 0.64 (95% CI: 0.49, 0.75), transport r = 0.44 (0.25, 0.60), TV r = 0.38 (0.18, 0.55); computer r = 0.40 (95% CI: 0.21, 0.57); reading r = 0.37 (95% CI: 0.17, 0.54); hobbies r = 0.36 (95% CI: 0.16, 0.53), other sedentary r = 0.22 (95% CI: 0.01, 0.42)
Craig, 2003	General population	IPAQ – SF, IPAQ-LF	Sitting	7 days	Spearman	IPAQ-LF: 0.28 to 0.93, IPAQ-SF: 0.18 to 0.95

First author, year	Population	Self-report measure		Reliability assessment		
		Name	Domain or type	Time between recall	Statistic used	Results
Doyle, 2018	Emirati university students	GPAQ (Arabic version)	Sitting	7 days	Spearman	0.44 (95% CI: 0.22, 0.64)
Duncan, 2019	Individuals with schizophrenia	IPAQ-SF	Sitting	4 weeks	ICC, spearman	Mean difference: 26.6 min, (95% LoA: -510.9, 564.2) and ICC = 0.41 (95% CI: 0.21, 0.59); spearman: ρ = 0.49, p < 0.001
Fjeldsoe, 2009	Women with young children	Australian Women's Activity Survey (AWAS)	Sitting (across multiple domains)	7 days	ICC	ICC = 0.42 (95% CI: 0.13, 0.64)
Fowles, 2017	University population	CSEP PASB-Q	Occupational, leisure & total sitting	7 days	correlation	work sitting r = 0.45, p<0.001; leisure sitting r = 0.66, p<0.001; total sedentary time r = 0.85, p<0.001
Gardiner, 2011	Older adults	Modified Salmon questionnaire	Watching TV, computer use, reading, socialising, transport and hobbies, and a summary measure (total sedentary time)	7 days	Spearman	Total sedentary time had acceptable test-retest reliability ρ = 0.52 (95% CI: 0.27 to 0.70)
Gennuso, 2015	Older adults	1) Yale Physical Activity Survey for Older Adults (YPAS), 2) CHAMPS	CHAMPS: SBs; YPAS: SBs	10 days	ICC	YPAS = 0.59 (P < .001), CHAMPS = 0.64 (P < .001)
Gennuso, 2016	Older adults	modified Salmon questionnaire (Gardiner)	(1) Watching TV, (2) using the computer, (3) reading, (4) socializing, (5) driving or in public transportation, (6) doing hobbies, and (7) any other activities.	1 week	ICC, Lin's concordance correlation	0.48, P<0.001
Hansen, 2014	General population	IPAQ-LF	Sitting (weekday + weekend)	2-3 WEEKS	ICC	0.66
Hekler, 2012	Older adults (65+)	CHAMPS	Sedentary time (TV, reading, socialize, passive transit, attend events)	6 months	ICC	0.56
Herrmann, 2013	General population	GPAQ, IPAQ	Sitting	GPAQ: Study 1: 10 days, study 2: 3 months	ICC	Study 1: ICC = 0.92 (95% CI: 0.78, 0.97), study 2: ICC = 0.83 (95% CI: 0.70, 0.90)
Ishii, 2018	Japanese adults	NR	Works and non-workdays sedentary time in: 1) car transport; 2) public transport; 3) at work; 4) watching TV, videos and DVDs; 5) using a computer, cell phone or tablet outside of work; 6) in leisure time.	2 weeks	ICC	workdays ICC = 0.77, p<.01, non-work days ICC = 0.53, p<.01, total ICC = 0.74, p<0.01
Jancey, 2014	Office workers	OSPAQ	Occupational sitting	7 days	ICC, t-test	0.66 (95% CI: 0.49, 0.77)

First author, year	Population	Self-report measure		Reliability assessment		
		Name	Domain or type	Time between recall	Statistic used	Results
Kohler, 2017	Healthy, community dwelling adults	cpar24	SBs	3 hours	Spearman	0.75
Larsson, 2019	Validity = office workers, reliability = seniors	SED-GIH	Sitting	Mean 5.2 days	ICC, weighted kappa	ICC = 0.86 (95% CI: 0.70, 0.90), kappa = 0.77 (95% CI: 0.68, 0.86)
Lopez-Rodriguez, 2017	Healthy elderly	EXERNET Questionnaire	Sitting	14 days	ICC	all = 0.68, men = 0.87, women = 0.65
Mader, 2006	Middle-aged women & men	IPAQ-SF	Sitting	14-21 days	Spearman	Total: 0.60, p<.05
Maher, 2017	Community-dwelling older adults	Daily diary	Sitting/lying time	1 day	ICC	0.56
Marshall, 2010	Middle-aged women & men	Marshall Sitting Questionnaire	Sitting (travel, work, TV, computer, leisure)	median = 11 days	ICC	Women: weekday work = 0.79 (95% CI: 0.73, 0.84); computer = 0.63 (95% CI: 0.52, 0.71); weekend day computer = 0.72 (95% CI: 0.64, 0.79). Men: weekday work = 0.86 (95% CI: 0.79, 0.90); TV = 0.65 (95% CI: 0.52, 0.75); computer = 0.62 (95% CI: 0.48, 0.72); weekend day TV = 0.62 (95% CI: 0.48, 0.73); computer = 0.59 (95% CI: 0.44, 0.71)
Matsuo, 2016	Workers	Workers' Sitting-and Walking-Time Questionnaire (WSWQ)	Occupational sitting, leisure sitting, non-workday sitting	7 days	ICC	Work time: time method = 0.85 (95% CI: 0.76, 0.91); % method = 0.83 (95% CI: 0.73, 0.89). Non-work time: time method = 0.49 (95% CI: 0.28, 0.66); % method = 0.71 (95% CI: 0.56, 0.81). Non-work day: time method = 0.64 (95% CI: 0.47, 0.76), % method: 0.78 (95% CI: 0.66, 0.86)
Matton, 2007	Employed/unemployed adults, retired adults	Flemish Physical Activity Computerized Questionnaire (FPACQ)	TV	2 weeks	ICC	Employed/unemployed: men: 0.93 (95% CI: 0.86, 0.97), women: 0.92 (95% CI: 0.84, 0.96). Retired: men 0.76 (95% CI: 0.49, 0.89), women 0.89(95% CI: 0.72–0.96)
Mensah, 2016	Healthy general population	Sedentary, Transportation and Activity Questionnaire (STAQ)	Total sitting, occupational sitting, transportation sitting, leisure sitting	1 months	ICC	total sitting: 0.52 (95% CI: 0.22, 0.73), work: 0.71 (95% CI: 0.49, 0.84], transport: 0.28 (95% CI: −0.06, 0.56), leisure: 0.37 (95% CI: 0.03, 0.62)
Murillo-Rabago, 2010	Healthy adults	IPAQ-SF & IPAQ-LF	Sitting	5 days	Spearman	IPAQ-SF: 0.73 (p<0.001), IPAQ-LF: 0.881 (p<0.001)
Pedersen, 2016	Desk-based workers	OSPAQ	Occupational sitting	1 week	ICC	0.44 (95% CI: 0.24 – 0.60)
Pedisic, 2014	Desk-based workers	Workplace Sitting Breaks Questionnaire (SITBRQ)	Duration and frequency of sitting breaks at work	7-14 days	Spearman	Frequency = 0.71 (95% CI: 0.59, 0.79), duration = 0.59 (95% CI: 0.45, 0.71)

First author, year	Population	Self-report measure		Reliability assessment		
		Name	Domain or type	Time between recall	Statistic used	Results
Peters, 2010	Urban adults	Past Year Physical Activity Questionnaire	SBs	1 year	ICC	total = 0.56, men = 0.61, women = 0.49
Rees-Punia, 2019	General population	7-day diary	Sitting	1 year	Spearman	Diary: 0.67 (95% CI: 0.63, 0.71)
Riviere, 2018	General population	GPAQ (French version)	Sitting	7 days	ICC	0.80 (95% CI: 0.69, 0.87)
Roman-Vinas, 2010	General population	IPAQ-LF	Sitting	3 days	Spearman	0.40, p<.01
Rosenberg, 2010	validity = overweight men & women	SBQ	SBs	2 weeks	ICC	total weekday = 0.848 (95% CI: 0.747, 0.911), total weekend day = 0.770 (95% CI: 0.626, 0.863)
Ryan, 2018	Community dwelling older adults	IPAQ-LF	Sitting	8-11 days	Spearman	0.26
Segura-Jimenez, 2013	Women with fibromyalgia	IPAQ-LF	Sitting, Sitting + travel	9 days	ICC	0.68 (95% CI: 0.58, 0.76)
Shuval, 2014	Primary care patients	Rapid Assessment Disuse Index (RADI)	Sitting (include sitting at home/work, watching TV and video/DVDs, on the computer at home/work, eating meals, etc)	12-16 days	ICC	week = 0.559 (95% CI: 0.442, 0.658), month = 0.576 (0.462, 0.672), year = 0.602 (95% CI: 0.491, 0.693)
Simpson, 2015	Healthy adults	Question 8 of the Paffenbarger Physical Activity Questionnaire	Sitting	3-6 months	ICC	0.71 (95% CI: 0.61, 0.74)
Sudholz, 2018	Workers	None	Sitting at work	7 days	ICC	0.78 (95% CI: 0.65, 0.86)
Toledo, 2017	US veterans & university employees	BeWell24 App	SBs	2 weeks	ICC	0.65 (95% CI: 0.43, 0.82)
Van Cauwenberg, 2014	Free-living older adults	NR	Sedentary time (TV, computer, reading, sedentary hobbies (e.g. handicraft, playing cards), seated conversation or listening to music, telephone, public transport, driving a car, being passenger in a car, sitting during household chores, resting, and eating	8 days	ICC	0.77 (95% CI: 0.57,0.89)
Van Der Ploeg, 2010	Workers	2006 Australian Bureau of Statistics time use survey	SB	7 days	ICC	0.55 (95% CI: 0.42, 0.66)

First author, year	Population	Self-report measure		Reliability assessment		
		Name	Domain or type	Time between recall	Statistic used	Results
Visser, 2013	older adults	LASA SB Questionnaire	Sum of SB (napping, reading, listening to music, watching TV, computer, working, hobby, on the phone, talking, transportation, church/theater	23 (8) days	ICC	0.71 (95% CI 0.57, 0.81)
Watson, 2017	Pregnant women	GPAQ	Sitting		ICC	0.05 (95% CI: -0.11, 0.22)
Whitfield, 2013	Recreational runners	Multi-context Sitting Time Questionnaire	Weekend & week day sitting across contexts	1 week	ICC	workday = 0.76 (95% CI: 0.50, 0.89), non-workday = 0.72 (95% CI: 0.42, 0.87)
Wijndaele, 2014	General population	Last 7-d sedentary behavior questionnaire (SIT-Q-7d)	Total sitting (meals, transportation, occupation, non-occupational screen time, and other ST)	1) 2 weeks, 2) 2-8 weeks	ICC	Sample 1: ICC = 0.68 (95% CI: 0.50, 0.81), sample 2: ICC = 0.53 (95% CI: 0.44, 0.62) **Note also provides domain specific ICCs

7-day SLIPA log – Sedentary and Light Intensity Physical Activity Log, CI – confidence interval, GPAQ – Global Physical Activity Questionnaire, ICC – intraclass correlation coefficient, MARCA – Multimedia Activity Recall for Children and Adolescents, MPAQ – Madras Diabetes Research Foundation Physical Activity Questionnaire, NQLS – Neighborhood Quality of Life Study, SB – sedentary behaviour, SEM – standard error of the mean, ST – sedentary time
